# Uncoupling tumor immunogenicity from cell death with platinum(IV)–antibody conjugates

**DOI:** 10.1093/nsr/nwag202

**Published:** 2026-04-02

**Authors:** Liu-Yi Liu, Wenhao Yu, Yilong Liu, Yang Yang, Qiuyang Wei, Zihan Zhao, Rong Yang, Jie P Li, Zijian Guo

**Affiliations:** State Key Laboratory of Coordination Chemistry, Chemistry and Biomedicine Innovation Center (ChemBIC), School of Chemistry and Chemical Engineering, Nanjing University, Nanjing 210023, China; State Key Laboratory of Coordination Chemistry, Chemistry and Biomedicine Innovation Center (ChemBIC), School of Chemistry and Chemical Engineering, Nanjing University, Nanjing 210023, China; State Key Laboratory of Coordination Chemistry, Chemistry and Biomedicine Innovation Center (ChemBIC), School of Chemistry and Chemical Engineering, Nanjing University, Nanjing 210023, China; State Key Laboratory of Coordination Chemistry, Chemistry and Biomedicine Innovation Center (ChemBIC), School of Chemistry and Chemical Engineering, Nanjing University, Nanjing 210023, China; State Key Laboratory of Coordination Chemistry, Chemistry and Biomedicine Innovation Center (ChemBIC), School of Chemistry and Chemical Engineering, Nanjing University, Nanjing 210023, China; Department of Urology, Nanjing Drum Tower Hospital, Affiliated Hospital of Medical School, Nanjing University, Nanjing 210008, China; Department of Urology, Nanjing Drum Tower Hospital, Affiliated Hospital of Medical School, Nanjing University, Nanjing 210008, China; State Key Laboratory of Coordination Chemistry, Chemistry and Biomedicine Innovation Center (ChemBIC), School of Chemistry and Chemical Engineering, Nanjing University, Nanjing 210023, China; State Key Laboratory of Coordination Chemistry, Chemistry and Biomedicine Innovation Center (ChemBIC), School of Chemistry and Chemical Engineering, Nanjing University, Nanjing 210023, China

**Keywords:** platinum(IV)–antibody conjugates, tumor immunogenicity, upregulation of MHC-I, chemo-immunotherapy combinations

## Abstract

Platinum drugs remain mainstays for solid tumors, but systemic toxicity and difficult dose finding in immunotherapy combinations limit their use. We engineer platinum(IV)–antibody conjugates [Pt-ADCs (platinum-based antibody–drug conjugates)] that confine a ‘metal immune effect’ to tumors while reducing off-tumor exposure. Site-specific glycoengineering installs redox-responsive Pt(IV) prodrugs at defined drug-to-antibody ratios, yielding homogeneous conjugates. Mechanistic profiling shows that cisplatin-derived, but not oxaliplatin-derived, payloads undergo efficient reductive activation in the tumor milieu; cinnamate-capped variants optimize serum stability, intratumoral release, and immunostimulatory signaling. In syngeneic models, Pt-ADCs deliver a low dose of cinnamate-capped Pt to upregulate major histocompatibility complex class I on tumor cells, expand tumor-reactive T-cell receptor clonotypes, and synergize with PD-1 blockade to suppress tumor growth, with minimal systemic toxicity. These findings position Pt-ADCs as a detoxified, immunogenic modality that uncouples immunogenic priming from high-dose cytotoxicity and offers a tractable path to rational dosing in chemo-immunotherapy combinations.

## Teaser

By uncoupling tumor immunogenicity from cell death, chemically well-defined platinum(IV)-antibody conjugates unlock strong synergy with anti-PD-1 and highlight the powerful therapeutic promise of low-dose platinum.

## INTRODUCTION

Despite the remarkable clinical success of immune checkpoint inhibitors (ICIs) in multiple malignancies, their efficacy remains limited to a subset of patients, with many tumors exhibiting primary or acquired resistance [1–3]. A major determinant of response is tumor immunogenicity, as tumors that fail to present sufficient antigens or actively suppress immune recognition often escape T cell-mediated killing [4]. Mechanistically, certain tumors downregulate major histocompatibility complex class I (MHC-I) molecules, alter antigen processing machinery, or create an immunosuppressive tumor microenvironment, collectively resulting in suboptimal responses to ICIs [5–9]. Consequently, strategies that enhance tumor immunogenicity or restore immune visibility have emerged as a critical avenue to broaden the patient population benefiting from immunotherapy [10,11]. Preclinical and clinical studies have demonstrated that increasing tumor antigen presentation or promoting tumor immunogenicity can sensitize resistant tumors to ICIs, highlighting the therapeutic potential of interventions that modulate tumor–immune interactions [12–16].

Platinum-based chemotherapies have long been employed as first-line treatments for a variety of solid tumors, achieving potent cytotoxic effects through DNA damage and apoptosis induction [17–19]. However, their lack of tumor specificity necessitates high systemic doses, leading to substantial toxicities, including immunosuppression and neurotoxicity, which restrict their further integration with immunotherapy [20,21]. Reducing the systemic burden of platinum drugs while maintaining efficacy has thus emerged as an urgent clinical need [22–25]. Beyond direct cytotoxicity, platinum agents can trigger immunogenic cell death (ICD)—a consequence of cytotoxic injury that exposes/releases damage-associated molecular patterns, such as surface calreticulin, extracellular adenosine triphosphate, and high mobility group box 1, and induces type I interferon (IFN) and inflammatory cytokines, thereby recruiting and activating dendritic cells [26–30]. These stress-driven, pro-inflammatory signals increase antigen presentation (notably MHC-I upregulation) and heighten T-cell recognition, providing a mechanistic basis for synergy with PD-1/PD-L1 blockade [31–33]. Platinum-induced DNA damage may also diversify tumor antigenicity (context-dependent), further sensitizing tumors to immune attack [34–36]. However, because ICD and immune activation arise from controlled cell injury, dose and schedule are pivotal: excessive exposure can suppress effector immunity or skew myeloid cells toward tolerogenic programs, blunting ICI benefit [37]. Clinically, dosing optimization is under active study—for example, a Bayesian adaptive randomized phase-2 trial showed that metronomic (low-dose, high-frequency) chemotherapy plus anti-PD-1 (αPD-1) is feasible and active in HER2-negative metastatic breast cancer [38]; in parallel, platinum-containing backbones with αPD-1 are being refined, including weekly low-AUC carboplatin with camrelizumab in neoadjuvant triple-negative breast cancer [39] and cisplatin/5-FU plus αPD-1 in head and neck squamous cell carcinoma [40]. Consequently, achieving precise dose modulation to maintain robust immune activation while minimizing systemic toxicity remains a critical challenge in platinum-based combination therapy development.

Based on these considerations, a key question arises: can platinum drugs be leveraged to retain their immunogenic potential while minimizing conventional cytotoxicity? By reducing the toxicity burden and effectively exploiting the ‘metal immune effect’, it may be possible to maximally enhance tumor immunogenicity and thereby improve the efficacy of ICIs. Here, we propose platinum-based antibody–drug conjugates (Pt-ADCs) as a viable solution. Pt-ADCs combine the tumor-targeting specificity of antibodies with the immunomodulatory properties of platinum prodrugs, enabling localized platinum release and effective activation of immune pathways while substantially reducing systemic toxicity. Our results show that the Pt-ADCs we constructed achieve low-dose, tumor-targeted platinum delivery. *In vivo*, because the Pt payload is released slowly in response to the tumor’s reductive milieu, it is insufficient to directly kill tumor cells. However, when combined with αPD-1 antibodies, Pt-ADCs sustain tumor cells in an MHC-I-upregulated state, thereby promoting T-cell receptor (TCR) clonal expansion and potent T cell-mediated cytotoxicity, ultimately producing marked tumor growth inhibition. This ‘detoxified yet immunogenic’ strategy could overcome current limitations of chemo-immunotherapy combinations and establish a new paradigm for leveraging platinum compounds in precision cancer immunotherapy.

## RESULTS

### Synthesis and characterization of Pt-ADCs

**Figure 1.**
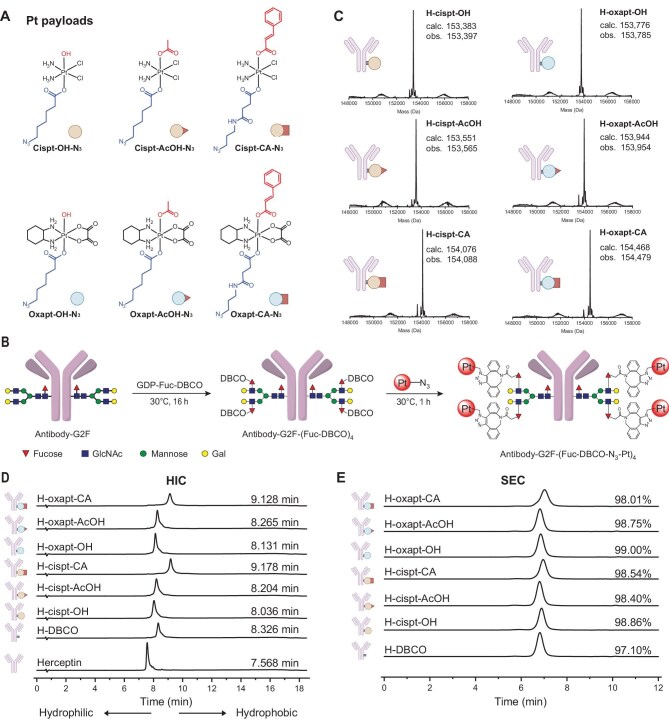
Synthesis and characterization of Pt-ADCs. (A) Chemical structures of Pt payloads for Pt-ADC construction. (B) Schematic representation of the structure and synthetic route of Pt-ADCs. (C) LC-MS analysis of Pt-ADCs. obs: observed; calc: calculated. (D) HIC profile of Pt-ADCs. (E) SEC profile of Pt-ADCs.

To develop Pt-ADCs, we designed and synthesized a series of Pt(IV) complexes derived from Pt(II) drugs including cisplatin and oxaliplatin scaffolds (Fig. 1A). One axial ligand of the Pt(IV) complex was modified with an azido group (N_3_) to enable conjugation to antibodies via click chemistry. The other axial site was modified to either fine-tune the release kinetics of the platinum payload or introduce functional moieties for potential synergistic effects. Three representative structures were designed: (i) an unmodified version bearing a hydroxyl group (OH), (ii) an acetato-substituted variant (AcOH), and (iii) a cinnamic acid-functionalized variant (CA). Anti-HER2 monoclonal antibodies, including Herceptin (commercial trastuzumab) and the bispecific antibody KN026, were employed for conjugation. Native antibodies bearing heterogeneous crystallizable fragment (Fc) glycans were glycoengineered to produce a homogeneous G2F glycan structure, introducing four LacNAc moieties per antibody. Site-specific introduction of dibenzocyclooctyne (DBCO) groups was subsequently achieved through fucosylation using fucosyltransferase and guanosine diphosphate β-L-fucose modified with DBCO (GDP-Fuc-DBCO) [41]. This process generated antibodies bearing four DBCO moieties at defined glycan sites. The azido-functionalized Pt(IV) complexes were then conjugated to the DBCO-modified antibodies via strain-promoted azide–alkyne cycloaddition to generate Pt-ADC constructs with a defined drug-to-antibody ratio (DAR) of 4 (Fig. 1B). Successful conjugation and structural integrity were confirmed by liquid chromatography-mass spectrometry (LC-MS) (Fig. 1C and Figs S1, S2).

Following conjugation, the hydrophilicity/hydrophobicity changes and aggregation state of the Pt-ADCs were assessed. Hydrophobic interaction chromatography (HIC) revealed only a modest increase in overall hydrophobicity after conjugation of the platinum payloads (Fig. 1D and Fig. S3). The extent of hydrophobicity correlated with the lipophilicity of the axial ligand of the Pt(IV) complexes, with Pt-ADCs bearing a CA group exhibiting greater hydrophobicity than their counterparts bearing OH or AcOH groups. For a given axial ligand, cisplatin-based Pt-ADCs displayed lower hydrophobicity compared to oxaliplatin-based counterparts. Size-exclusion chromatography (SEC) analysis demonstrated that over 98% of the Herceptin-based Pt-ADC population remained as monomers, indicating that payload conjugation did not promote antibody aggregation (Fig. 1E).

### Low-dose tumor-targeted platinum delivery by Pt-ADCs

Following Pt-ADC construction, we first evaluated the binding, cell uptake, and tumor-targeting properties of Pt-ADCs to understand their cellular behavior. Cell-based binding assays confirmed that the conjugation of platinum payloads did not affect the ability of the antibody to recognize and bind HER2-expressing cells (Fig. S4). Endocytosis assays further demonstrated that Pt-ADCs were efficiently internalized into CT26-HER2 cells, but not into HER2-negative CT26 control cells, indicating HER2-mediated uptake of Pt-ADCs (Fig. S5). Quantification of intracellular platinum by inductively coupled plasma mass spectrometry (ICP-MS) revealed efficient uptake of platinum, with platinum predominantly localized in the cytoplasm and to a lesser extent in the nucleus, a distribution pattern resembling that of small-molecule platinum drugs (Fig. S6). This suggests that antibody-mediated endocytosis enables platinum payloads to reach the nucleus and exert their effects.

**Figure 2.**
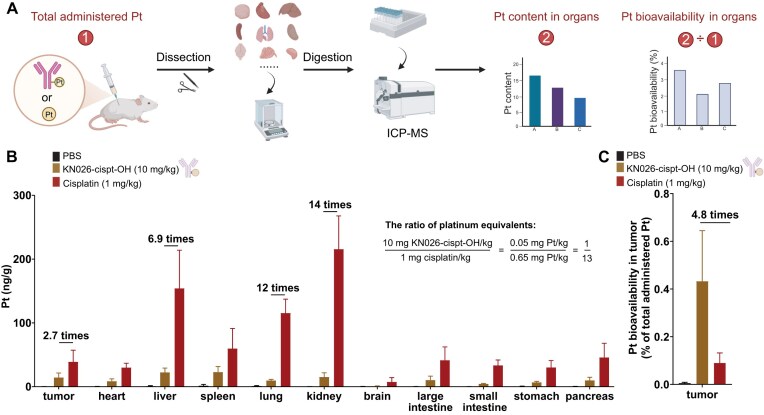
Low-dose tumor-targeted platinum delivery by Pt-ADCs. (A) Schematic of the assay showing the distribution of platinum content in mouse tumors and various organs following Pt-ADC or cisplatin administration. (B) Platinum levels in tumors and major organs of CT26-HER2 tumor-bearing mice, measured by ICP-MS, 48 h after intraperitoneal injection of Pt-ADC or intravenous injection of cisplatin. (C) Platinum content in tumors as a percentage of the total administered dose, reflecting the bioavailability of platinum in tumors.

To characterize the *in vivo* biodistribution of Pt-ADCs, platinum levels in tumors and major organs of HER2-positive tumor-bearing mice were quantified using ICP-MS (Fig. 2A). Compared with low-dose cisplatin (1 mg/kg; 0.65 mg Pt/kg), high-dose Pt-ADC administration (10 mg/kg; 0.05 mg Pt/kg; platinum content 13-fold lower than cisplatin administration) resulted in ∼2.7-fold lower absolute platinum content in tumors, indicating that only a very small amount of platinum was delivered to tumor tissue via the Pt-ADC format (Fig. 2B and Table S1). However, platinum accumulation in non-tumor organs, including the liver, kidney, and lung, was markedly reduced by 6.9-, 14-, and 12-fold, respectively, relative to cisplatin, highlighting the potential of Pt-ADCs to mitigate toxicity resulting from lack of tumor targeting. To assess tumor-specific bioavailability, the proportion of platinum in tumors relative to the total administered dose was calculated, with 0.43 ± 0.18% for Pt-ADC administration and 0.09 ± 0.04% for cisplatin administration. Even with Pt-ADCs, platinum bioavailability in tumors remains relatively low. Notably, Pt-ADCs achieved ∼4.8-fold higher platinum distribution in tumors compared with cisplatin, underscoring the enhanced tumor-targeting efficiency conferred by antibody conjugation (Fig. 2C).

We then investigated the functional impact of Pt-ADCs on tumor cells. Pt-ADCs exhibited cytotoxic effects against HER2^high^ NCI-N87 cells (a human gastric cancer cell line with high endogenous HER2 expression) comparable to those of the unconjugated antibody, indicating that the platinum payload did not significantly enhance direct tumor cell killing under these conditions. In addition, Pt-ADCs showed no cytotoxicity toward HER2^low^ A549 cells (a human non-small cell lung cancer cell line with low endogenous HER2 expression), consistent with the ‘low-dose, non-cytotoxic’ characteristics of Pt-ADC delivered platinum (Fig. S7). Additionally, in the NCI-N87 subcutaneous xenograft model in BALB/c nude mice, we evaluated the direct tumor-killing effect of Pt-ADCs (Fig. S8A). The results demonstrated that Pt-ADC exhibited tumor inhibition similarly to the unconjugated antibody, further confirming that Pt-ADCs deliver low-dose platinum to tumor cells without exerting direct cytotoxic effects (Fig. S8B). Moreover, the treatment did not affect mouse body weight, suggesting no significant systemic toxicity (Fig. S8C). Notably, even at low doses, cisplatin-based Pt-ADCs induced upregulation of MHC-I (Figs S9–S11), indicating that low-dose platinum, without significant cytotoxicity, is sufficient to enhance tumor cell antigen presentation, suggesting a potential role in promoting immunogenicity via stress-related pathways.

### Selection of Pt payloads synergizing with αPD-1

**Figure 3.**
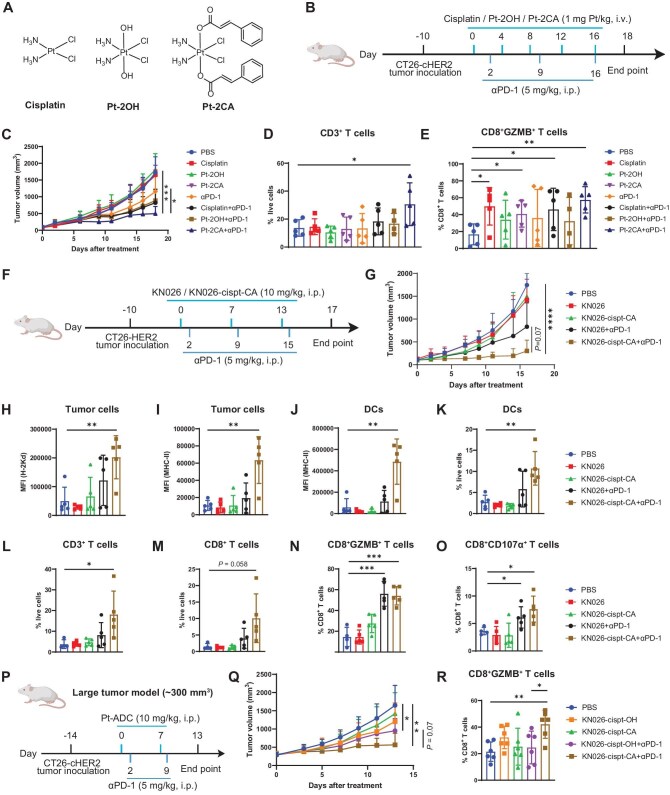
*In vivo* antitumor efficacy of Pt-ADC in combination with αPD-1 therapy. (A) Chemical structures of small-molecule platinum agents. (B) Schematic representation of the combination treatment regimen with small-molecule platinum agents and αPD-1 in the CT26-cHER2 tumor model. i.v.: intravenous; i.p.: intraperitoneal. (C) Tumor volume changes during the course of treatment described in panel (B). (D and E) Flow cytometric analysis of tumors collected at the endpoint of the treatment in panel (B). (D) Percentage of CD3^+^ T cells among live cells. (E) Percentage of CD8^+^GZMB^+^ T cells within the CD8^+^ T cell population. (F) Schematic representation of the combination treatment regimen with Pt-ADCs and αPD-1 in the CT26-HER2 tumor model. (G) Tumor volume changes during the course of treatment described in panel (F). (H–O) Flow cytometric analysis of tumors collected at the endpoint of the treatment in panel (F). (H and I) MHC-I (H-2Kd) and MHC-II (I-A/I-E) expression on CT26-HER2 tumor cells. (J) MHC-II expression on DCs. (K–M) Percentage of DCs, CD3^+^ T cells, and CD8^+^ T cells among live cells. (N and O) Percentage of CD8^+^GZMB^+^ T cells and CD8^+^CD107α^+^ T cells within CD8^+^ T cell population. (P) Schematic representation of the combination treatment regimen with Pt-ADCs and αPD-1 in the large CT26-cHER2 tumor model. (Q) Tumor volume changes during the course of treatment described in (P). (R) Flow cytometric analysis of tumors collected at the endpoint of the treatment in (P). Percentage of CD8^+^GZMB^+^ T cells within CD8^+^ T cell population. Data are mean ± SD (*n* ≥ 3). *P < 0.05, **P < 0.01, ***P < 0.001.

Because Pt-ADCs deliver intentionally low, non-cytotoxic amounts of platinum, payload selection must prioritize immunoactivity; accordingly, we screen Pt(IV) candidates that maximally potentiate immunogenic signaling and synergize with αPD-1 to improve efficacy. To investigate the relationship between Pt payload structure and immunostimulatory potential, we designed different cisplatin-derived small molecules (Pt-2OH and Pt-2CA; Fig. 3A) and evaluated their ability to activate immune responses.

Cytotoxicity assays revealed that Pt-2CA exhibited the highest cell-killing activity (Fig. S12), followed by cisplatin and then Pt-2OH, consistent with the poor solubility and limited cellular uptake of Pt-2OH (Fig. S6). Importantly, all three small molecules, when administered near their respective IC_50_ values, significantly upregulated MHC-I expression (Fig. S13). Notably, cisplatin and Pt-2CA remained effective in inducing MHC-I upregulation at doses lower than 2–3 times their respective IC_50_ values (Figs S14 and S15). Transcriptomic analysis further showed that, compared to the effects on abnormal metabolism and DNA damage response, low-dose Pt-2CA treatment markedly enhanced pathways related to antigen processing and presentation (Figs S16 and S17). Furthermore, western blot and flow cytometry analyses demonstrated that low-dose Pt-2CA treatment activates the cyclic GMP–AMP synthase (cGAS) stimulator of IFN genes (STING) signaling pathway and induces reactive oxygen species (ROS) accumulation, while markers of the endoplasmic reticulum stress pathway exhibited minimal changes (Figs S18 and S19). Collectively, these results indicate that low/sub-cytotoxic Pt-2CA exposure enhances tumor cell immunogenicity through multiple mechanisms, including MHC-I upregulation, activation of the cGAS-STING pathway, and ROS-associated stress signaling.

Subsequent *in vivo* treatment studies demonstrated that low-dose small-molecule platinum agents alone had minimal antitumor effects. In contrast, combination treatment with Pt-2CA and αPD-1 produced the most pronounced tumor inhibition: αPD-1 alone achieved 33% suppression compared to the PBS control, cisplatin plus αPD-1 achieved 52%, Pt-2OH plus αPD-1 achieved 48%, and Pt-2CA plus αPD-1 achieved 71%, indicating a clear synergistic effect between Pt-2CA and αPD-1 (Fig. 3B and C). Combination treatment with Pt-2CA and αPD-1 also increased the proportions of CD3^+^ and CD8^+^ T cells within the tumor (Fig. 3D and Fig. S20). Notably, within the CD8^+^ T cell population, the frequency of granzyme B^+^ (GZMB^+^) cells was significantly elevated (Fig. 3E), reflecting enhanced cytotoxic activity. Moreover, Pt-2CA treatment increased the proportions of CD8^+^GZMB^+^ and CD8^+^CD107α^+^ T cells, whereas cinnamic acid (CA) alone exerted minimal effects on these populations (Figs S21 and S22). Collectively, CA monotherapy is insufficient to potentiate antitumor immunity but confers synergistic immune enhancement when coordinated with platinum drug. These findings collectively identified the CA-substituted Pt payload as the optimal candidate for incorporation into Pt-ADCs for subsequent *in vivo* studies.

### Synergistic antitumor efficacy of Pt-ADC and αPD-1 combination therapy

After confirming that small-molecule platinum agents can activate tumor immunogenicity, we next evaluated the therapeutic potential of Pt-ADCs and their combination with immune checkpoint blockade. In this study, Herceptin-based Pt-ADCs were primarily used to establish proof of concept, while KN026-based Pt-ADCs, due to KN026’s superior *in vivo* therapeutic efficacy as an HER2-targeted bispecific antibody [42], were mainly employed for *in vivo* validation. CT26-HER2 (a mouse colon cancer cell line expressing exogenous human HER2, with HER2 overexpression confirmed by flow cytometric analysis; Figs S23 and S24) tumors were implanted subcutaneously into BALB/c mice, and animals were treated with KN026-cispt-CA (10 mg/kg), either alone or in combination with an αPD-1 antibody (5 mg/kg) (Fig. 3F). Throughout the treatment course, no significant changes in body weight were observed, indicating good tolerability of the regimens (Fig. S25A). While KN026-cispt-CA monotherapy exhibited limited tumor suppression, the combination therapy resulted in the most pronounced inhibition of tumor growth. In KN026-cispt-CA and αPD-1 combination group, tumor volume was reduced by 83% compared to the PBS control, by 79% compared to the KN026 group, by 80% compared to the KN026-cispt-CA group, and by 64% compared to the KN026 and αPD-1 combination group (Fig. 3G and Fig. S25B). Notably, the Pt-ADC-based regimen achieved superior tumor growth inhibition compared with the co-administration of unconjugated antibody and free platinum compound, further supporting the therapeutic advantage of the Pt-ADC format over simple platinum drug and antibody combination strategies (Fig. S26).

At the treatment endpoint, flow cytometry analysis of tumor tissues revealed that the KN026-cispt-CA and αPD-1 combination group exhibited increased expression of MHC-I (H-2Kd) and MHC-II (I-A/I-E) on tumor cells, enhancing their visibility to cytotoxic T lymphocytes (Fig. 3H and I). Moreover, MHC-II expression on dendritic cells (DCs) and tumor-associated macrophages (TAMs) was also upregulated, promoting antigen presentation and immune activation (Fig. 3J and Fig. S27A). Importantly, the ratio of M1 to M2 macrophages in the tumor microenvironment was increased, indicative of a shift toward a more immunostimulatory phenotype (Figs S27B–E and S28). Immunophenotyping further revealed elevated infiltration of DCs and T cells, including CD3^+^, CD4^+^, and CD8^+^ subsets, in tumors from the combination treatment group (Fig. 3K–M and Figs S27F and S29). Notably, within the CD8^+^ T cell population, the frequencies of GZMB^+^ and CD107α^+^ cells were significantly increased, reflecting enhanced cytotoxic activity (Fig. 3N and O and Fig. S30). Together, these results demonstrate that the combination of Pt-ADC and αPD-1 therapy effectively remodels the tumor immune microenvironment, potentiates T cell-mediated cytotoxicity, and significantly enhances antitumor efficacy. The therapeutic outcome suggests a synergistic interaction, wherein the combined effect exceeds the additive benefits of each monotherapy, exemplifying a therapeutic synergy where the whole is greater than the sum of its parts.

Further validation in a large-tumor model confirmed the superior efficacy of KN026-cispt-CA in combination with αPD-1 (Fig. 3P). KN026-cispt-CA monotherapy led to negligible tumor growth inhibition (with tumor growth reduced by 14%), whereas its combination with αPD-1 suppressed tumor growth by 66% (Fig. 3Q and Fig. S31). KN026-cispt-OH monotherapy reduced tumor growth by 27%, and its combination with αPD-1 achieved only modest synergy, with 42% inhibition. Flow cytometry analysis further showed that within the CD8^+^ T cell population, the frequency of GZMB^+^ cells was significantly elevated in the KN026-cispt-CA plus αPD-1 group (Fig. 3R), indicating enhanced cytotoxic activity. The inferior efficacy of KN026-cispt-OH plus αPD-1, compared with the KN026-cispt-CA regimen, is consistent with the weaker capacity of the unmodified Pt payload to induce immunogenicity *in vivo*.

Following five administrations of KN026-cispt-CA (10 mg/kg), serum biochemical markers were evaluated, and histological examination of major organs was conducted after hematoxylin–eosin (H&E) staining (Figs S32 and S33). KN026-cispt-CA demonstrated low systemic toxicity, while low-dose cisplatin (1 mg/kg) induced abnormal liver and renal function indicators in some mice. These results suggest that KN026-cispt-CA offers effective treatment with reduced side effects through low-dose, tumor-targeted platinum delivery.

### Reductive behavior of Pt payloads and its impact on therapeutic activity

**Figure 4.**
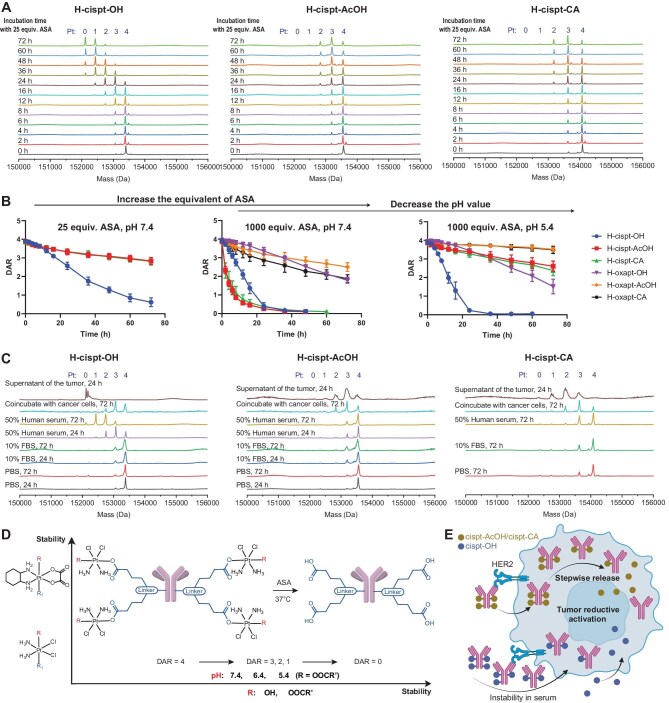
Reductive behavior of Pt-ADCs under various conditions. (A) LC-MS analysis of the stepwise reduction of Pt-ADCs in the presence of ASA (25 equiv., pH 7.4, PBS). (B) Reduction rate of Pt-ADCs under varying equivalents of ASA and different pH conditions. (C) Reduction rate of Pt-ADCs in different serum conditions, cell culture media, and tumor-mimicking environments. (D) Schematic representation and summary of structural changes during Pt-ADC reduction. (E) Mechanistic scheme illustrating the release of Pt-ADC payload in tumors.

The reduction of Pt(IV) payloads to Pt(II) species triggers the release of the Pt payloads (Fig. S34). The reduction rate of Pt(IV) prodrugs is closely related to the nature of their axial ligands [43], while the reductive behavior of Pt(IV) prodrugs impacts the local concentration and duration of the platinum drug at the tumor site, a key factor in determining the balance and bias between tumor immunogenicity and cell death. To better understand distinct immunological and therapeutic outcomes observed with different Pt-ADC structures, we systematically investigated the reductive behavior of the platinum payloads in detail using LC-MS. In the presence of ascorbic acid (ASA), LC-MS analysis identified species bearing four, three, two, one, or zero platinum payloads conjugated to the antibody. Over time, a gradual decrease in the number of conjugated platinum payloads was observed, indicating that the platinum cores underwent stepwise reduction, releasing the active platinum payload while retaining the azido-bearing axial ligand covalently linked to the antibody (Fig. 4A, B, D and Figs S35, S36, and S39). Among the different constructs, the cisplatin-based ADC with an OH axial ligand (H-cispt-OH) exhibited the most rapid release kinetics. Under physiological conditions (PBS, pH 7.4) with 25 equivalents of ASA, nearly complete platinum payload release was observed within 72 h. In contrast, the cisplatin-based ADCs bearing acetato-derived axial ligands, such as acetate (H-cispt-AcOH) and CA (H-cispt-CA), required higher equivalents of ASA to achieve comparable levels of release under the same conditions. Consistent with the similar nature of their axial substituents, the AcOH- and CA-modified Pt-ADCs displayed comparable reduction profiles. Oxaliplatin-based Pt-ADCs displayed substantially slower reduction rates. Even under 1000 equivalents of ASA and 72 h of incubation, only partial (∼50%) release of the payload was observed (Fig. 4B, D and Figs S35, S36, S39). These reduction profiles are consistent with biological evidence showing that cisplatin-based Pt-ADCs upregulate MHC-I in tumor cells, whereas oxaliplatin-based Pt-ADCs do not, owing to the greater reducibility and activation of cisplatin payloads.

To mimic the acidic milieu of lysosomes, we further evaluated payload release under low-pH conditions. Reducing the pH from 7.4 to 5.4 had little effect on the release kinetics of OH-modified Pt-ADCs. In contrast, acidic conditions significantly suppressed the release of platinum payloads containing acetato-derived axial ligands (AcOH and CA) (Fig. 4B, D and Figs S36–S39). These results suggest that axial ligand structure is a key determinant of payload release kinetics of Pt-ADCs. Importantly, the attenuated release of carboxylato-modified platinum payloads under acidic conditions may help prevent premature payload dissociation within the acidic lysosomal compartments following ADC internalization, potentially reducing payload deactivation and improving therapeutic efficacy. In addition, Pt-ADCs constructed with either Herceptin or KN026 exhibited comparable stability and reduction rates, indicating minimal dependence on the antibody scaffold (Fig. S40). Crucially, this redox-responsive release mechanism is mechanistically distinct from traditional ADC linker systems, such as acid-labile hydrazones or protease-cleavable dipeptides. While conventional linkers rely on lysosomal acidity or enzymatic activity for payload liberation, Pt-ADCs exploit the intracellular reducing environment, particularly the abundance of reducing agents like ascorbate and glutathione, to trigger payload release.

We next assessed the stability of cisplatin-based ADCs under physiologically relevant conditions, including serum-containing buffers, cell culture media, and tumor-mimicking environments (Fig. 4C and E). In PBS with 10% fetal bovine serum (FBS), H-cispt-OH, H-cispt-AcOH, and H-cispt-CA all remained stable over 72 h. However, upon increasing the serum concentration to 50% human serum, partial release of H-cispt-OH was observed, with DARs of 1.6 after 72 h, while H-cispt-AcOH and H-cispt-CA maintained stability under the same conditions. In *in vitro* cell culture media (10% FBS in RPMI 1640 medium with CT26 cell culture), H-cispt-OH, H-cispt-AcOH, and H-cispt-CA only showed a small portion of release after 72 h, with DARs of 3.0, 2.8, and 2.7, respectively, consistent with their lack of *in vitro* cytotoxicity. Remarkably, when incubated in supernatant (not lysates) collected from *in vivo* CT26 tumors, which simulates the biochemical conditions of the tumor microenvironment, H-cispt-OH underwent complete release within 24 h, while H-cispt-AcOH and H-cispt-CA also exhibited moderate payload release, with a DAR of 2.6 and 2.5, respectively. These results highlight the selective activation of Pt-ADCs, driven by the reductive nature of the tumor microenvironment. Furthermore, the enhanced serum stability of Pt-ADCs bearing carboxylato-modified axial ligands suggests reduced premature payload release during systemic circulation, potentially minimizing systemic toxicity and improving therapeutic index. Overall, cispt-OH-based ADC exhibited markedly faster Pt payload release than KN026-cispt-CA, which explains its modest antitumor activity as monotherapy. However, its combination with PD-1 blockade was less effective than cispt-CA-based ADC combination, consistent with the abovementioned observed differences in tumor immunogenicity induction. Based on its rapid endocytosis and gradual release behavior, the majority of cisplatin-CA-based ADC is endocytosed by tumor cells, releasing the platinum payload intracellularly, while a portion of the platinum payload is released in the tumor microenvironment. This gradual release rate maintains platinum concentration at levels that do not directly kill tumor cells, yet enhance tumor cell immunogenicity, explaining the stronger synergy with PD-1 blockade. These findings further underscore the structural advantage of cispt-CA-based ADC in achieving both stability and tumor-specific activation.

### Tumor microenvironment reprogramming by Pt-ADC and αPD-1 combination therapy

**Figure 5.**
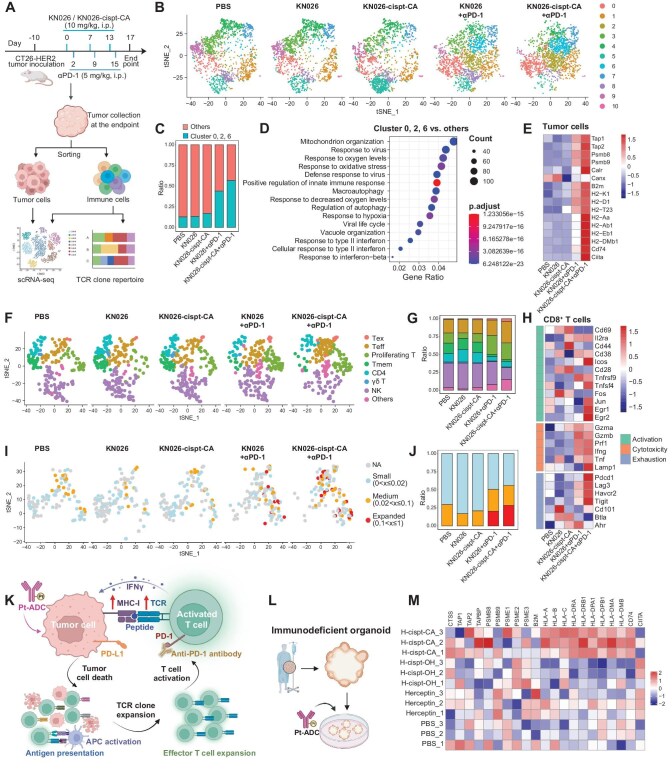
Remodeling of the tumor microenvironment by Pt-ADCs. (A) Schematic of the combination treatment regimen in the CT26-HER2 tumor model. Tumor cells and CD45^+^ tumor-infiltrating immune cells were isolated from tumors of treated mice (*n* = 5 per group) by flow cytometry, followed by scRNA-seq analysis. (B) t-SNE plots showing sorted tumor cells collected at the endpoint of the treatment in panel (A). (C) Proportions of the cluster 0, 2, and 6 within the tumor cell population. (D) GO terms for the differentially expressed genes of clusters 0, 2, and 6 compared with other clusters, indicating that the tumor cell subpopulations increased after combination therapy are associated with enhanced immunogenicity. (E) Heatmap showing the expression of selected genes in tumor cells across treatment groups. (F) t-SNE plots showing re-clustered T and NK cell subsets. (G) Proportions of each subset within T and NK cell subsets. (H) Heatmap showing the expression of representative genes in CD8^+^ T cell subsets across different treatment groups. (I) t-SNE plots showing CD8^+^ T cells colored by TCR clonality. (J) Distribution of TCR clonality levels within the CD8^+^ T cell population, indicating that the combination therapy group enhances TCR clonal expansion. (K) Schematic illustration of the proposed therapeutic mechanism underlying the combination of Pt-ADC with PD-1 blockade. (L) Schematic of the HER2-positive immunodeficient PDO model used for Pt-ADC testing (300 μg/mL, 84 h). (M) Heatmap of selected gene expression profiles across treatment groups of Patient 1, as described in panel (L).

To elucidate how KN026-cispt-CA mediates antitumor activity via low-dose platinum release, we performed single-cell RNA sequencing (scRNA-seq) to profile both tumor and immune cell populations within the tumor microenvironment (Fig. 5A). At the treatment endpoint, tumor cells were isolated from CT26-HER2 tumors for scRNA-seq analysis. T-distributed stochastic neighbor embedding (t-SNE) dimensionality reduction revealed an expansion of specific tumor cell subpopulations (Clusters 0, 2, and 6) in the KN026-cispt-CA plus αPD-1 group (Fig. 5B, C and Fig. S41). These subpopulations were enriched for gene signatures associated with immune response and IFN signaling (Fig. 5D). Moreover, the KN026-cispt-CA plus αPD-1 group markedly increased MHC expression on tumor cells (Fig. 5E), indicating that Pt-ADC facilitates tumor immunogenicity and enhances antigen presentation. This suggests that sustained low-dose platinum release from KN026-cispt-CA reprograms tumor cells to become more immunologically visible, thereby showing the potential to potentiate T cell-mediated cytotoxicity.

We next analyzed immune cells within the tumor microenvironment. At the treatment endpoint, CD45^+^ immune cells were isolated from CT26-HER2 tumors for scRNA-seq analysis. Quantification of major immune lineages within the CD45^+^ compartment revealed a higher proportion of DCs in the KN026-cispt-CA plus αPD-1 combination group (Fig. S42A and B). Transcriptomic profiling of DCs showed that the KN026-cispt-CA plus αPD-1 treatment robustly upregulated gene signatures related to antigen processing and presentation, IFN response, chemokine signaling, and T cell priming, while genes associated with immunosuppression were downregulated compared with all other treatment groups (Fig. S42C). These results indicate that DCs in the combination group adopt a more immunostimulatory phenotype, enhancing their capacity to prime effector T cell responses. Similarly, macrophage profiling revealed that KN026-cispt-CA combined with αPD-1 induced upregulation of gene signatures associated with antigen presentation, IFN signaling, chemokine production, and effector function, whereas immunosuppressive and M2-like gene programs were downregulated relative to other treatments (Fig. S42D). This shift suggests a reprogramming of TAMs toward a pro-inflammatory, antitumor phenotype, supporting the observed enhancement of antitumor immunity. Notably, a neutrophil subpopulation exhibiting high tumor-promoting T3-type scores was significantly reduced in the combination group (Fig. S42E), and T3-associated gene expression was decreased following combination therapy (Fig. S42F) [44]. These findings suggest that KN026-cispt-CA, through antibody-mediated tumor targeting, does not impair immune-supportive cells, thereby minimizing immune toxicity. Notably, KN026-cispt-CA enhances the immunogenicity of tumor cells, effectively activating the immune system and ultimately reprogramming the tumor microenvironment from an immunosuppressive to an immunostimulatory state.

Re-clustering analysis of T cell and NK cell subsets revealed that KN026-cispt-CA plus αPD-1 treatment increased the proportion of effector T cell populations (Fig. 5F and G). This suggests that the combination therapy reshapes the T cell landscape toward a more cytotoxic phenotype. Comparative analysis of CD8^+^ T cells across treatment groups showed upregulation of genes associated with activation and cytotoxic function in the KN026-cispt-CA plus αPD-1 group (Fig. 5H), indicating enhanced CD8^+^ T cell-mediated killing. Notably, exhaustion-associated genes were also upregulated, which suggests that sustained antigen stimulation in the combination group drives both effector differentiation and concomitant exhaustion, indicative of a robust yet persistent T cell response. TCR clonotype analysis further demonstrated the most pronounced clonal expansion of CD8^+^ T cells in the KN026-cispt-CA plus αPD-1 group (Fig. 5I and J). This indicates that the combination therapy not only activates CD8^+^ T cells but also promotes selective expansion of tumor-reactive clones, thereby reinforcing antitumor immunity. Gene ontology (GO) enrichment analysis supported these observations. Relative to the KN026 monotherapy group, the KN026-cispt-CA monotherapy group significantly affected pathways related to cell cycle and proliferation (Fig. S43A), consistent with the known effects of platinum drugs on tumor cell division. Importantly, compared with the KN026 plus αPD-1 group, the KN026-cispt-CA plus αPD-1 group strongly impacted pathways associated with T cell activation, differentiation, and effector function (Fig. S43B), in line with the enhanced antitumor responses observed *in vivo*. These results suggest that KN026-cispt-CA maintains tumor cells in an MHC-upregulated state, facilitating efficient antigen presentation and recognition by T cells. When combined with αPD-1, this approach further alleviates checkpoint-mediated inhibition and sustains T cell effector activity, ultimately promoting TCR clonal expansion and robust T cell-mediated cytotoxicity (Fig. 5K and Fig. S44).

### Pt-ADC activity in patient-derived tumor models

We next assessed the activity of Pt-ADCs in HER2-positive patient-derived organoids (PDOs). In four immunodeficient PDO models (Fig. 5L), KN026-cispt-CA (or H-cispt-CA) elicited little direct killing, with PDO viability remaining above 50% (Fig. S45A–E), consistent with our earlier observation that KN026-cispt-CA exerts its primary effects through mechanisms other than direct tumor cell killing. In addition, KN026-cispt-CA (or H-cispt-CA) was less cytotoxic than KN026-cispt-OH (or H-cispt-OH), in line with the *in vivo* monotherapy results. Notably, Pt-ADCs enhanced tumor cell immunogenicity across all PDOs, as evidenced by upregulation of antigen processing and presentation pathways (Fig. 5M and Fig. S45F–H), indicating that even low-dose platinum can effectively promote MHC upregulation in PDO models. GO enrichment further revealed that KN026-cispt-CA (or H-cispt-CA) more robustly induced pathways related to protein misfolding, MHC upregulation, and IFN responses compared with KN026-cispt-OH (or H-cispt-OH) (Fig. S45I–K). These findings suggest that KN026-cispt-CA more effectively enhances tumor immunogenicity, in agreement with its superior therapeutic performance in animal models. These findings further suggest that, within a strategy that relies on low-dose platinum to stimulate tumor immunogenicity without direct cytotoxicity, incorporating functional ligands that synergistically enhance tumor immunogenicity can provide additional benefits for subsequent therapeutic efficacy.

In immunocompetent organoids, treatment with KN026-cispt-CA, either alone or in combination with αPD-1, partially suppressed organoid proliferation (Fig. S46). The limited efficacy observed in immunocompetent organoids may be attributed to its distinct mechanism of action. The mechanism of KN026-cispt-CA, which involves antibody-mediated targeting and internalization into tumor cells, followed by the gradual, low-dose release of the platinum payload to upregulate MHC-I on tumor cells and expand tumor-reactive TCR clonotypes, unfolds on a slower timescale than that of small-molecule platinum drugs. In organoid models, however, immune cells typically persist for only a few days, which may not be long enough to mount a full effector response [45]. By contrast, *in vivo*, immune cells can persist for over several weeks, enabling a more sustained immune response. *In vivo*, Pt-ADC treatment induces more robust antitumor activity, likely due to the recruitment of T cells from draining lymph nodes into the tumor microenvironment, which facilitates sustained T cell-mediated cytotoxicity [46,47]. Together, these results indicate that Pt-ADCs exert antitumor effects primarily through low-dose delivery combined with potent immunostimulatory activity rather than direct cytotoxicity. This unique mechanism not only remodels the tumor immune microenvironment and enhances responsiveness to immune checkpoint blockade, but also minimizes platinum-induced immunotoxicity and other systemic toxicities.

## DISCUSSION

We report, for the first time, the development of ADCs featuring quantitative cisplatin-based payloads and provide a detailed investigation of their reductive behavior. The design of Pt-ADCs necessitates careful consideration of platinum’s coordination chemistry. Traditional Pt(II) agents, such as cisplatin derivatives, possess labile Pt(II)–Cl bonds that are prone to premature hydrolysis and deactivation during bioconjugation or systemic circulation. To address this limitation, we adopted a Pt(IV) prodrug approach. Compared to Pt(II)–Cl bonds, Pt(IV)–Cl bonds exhibit significantly enhanced kinetic stability, ensuring the integrity of the conjugate during systemic exposure [48]. Crucially, Pt(IV) complexes are redox-responsive and can be reduced to active Pt(II) species under the reducing conditions characteristic of the tumor microenvironment. This enables the controlled release of active platinum drugs without the need for additional cleavable linkers. Furthermore, the axial positions on the Pt(IV) center offer unique chemical handles for functionalization. By installing a bioactive moiety in the second axial site, dual-functional payloads can be constructed to achieve synergistic therapeutic effects.

This redox-activated strategy offers several notable features over conventional ADC platforms. First, payload release is confined to the reductive tumor microenvironment, thereby minimizing systemic toxicity. Second, the axial ligand architecture allows programmable modulation of release kinetics through rational ligand design. Third, the elimination of exogenous linker chemistry simplifies the molecular structure, potentially improving synthetic efficiency, conjugate stability, and batch-to-batch consistency. Fourth, the use of clinically validated platinum drugs, such as cisplatin and oxaliplatin, provides a pharmacological foundation with established safety and efficacy profiles. Finally, the easily modifiable nature of the axial ligand of Pt(IV) enables the development of multifunctional payloads, supporting co-delivery strategies that combine cytotoxicity with immunomodulation or targeted signaling disruption.

The immunogenic effects of platinum drugs are closely linked to cellular stress, and excessive dosing may paradoxically impair immune function, underscoring the need for precise dose modulation to balance cytotoxicity and immunostimulation. Unlike conventional small-molecule platinum drugs, Pt-ADCs achieve low-dose, targeted delivery through antibody conjugation, thereby mitigating the systemic toxicity associated with the non-selective distribution of small molecules. In contrast to previously reported cytotoxic Pt-ADCs [24,25], which aim for direct cytotoxicity for antitumor effects, our immunogenic Pt-ADCs introduce intentional low-dose, low-cytotoxic immunogenic priming. This low-dose delivery does not induce direct cytotoxicity toward tumor cells, but effectively enhances tumor immunogenicity, ultimately improving the efficacy of ICIs. Compared with KN026-cispt-OH (or H-cispt-OH), KN026-cispt-CA (or H-cispt-CA) exhibits a slower reduction rate, resulting in gradual and more sustained low-dose platinum exposure, which maintains tumor cells in a state of persistent MHC upregulation. This creates a prolonged temporal window for T cell recognition and facilitates deeper intratumoral drug penetration. In addition, the axial ligand of the platinum payload not only allows precise tuning of release kinetics but also provides a platform to incorporate functional ligands that further enhance tumor immunogenicity. Taken together, precise modulation of platinum dosage and release kinetics sustains tumor cells in an MHC-upregulated state, thereby promoting TCR clonal expansion and potent T cell-mediated cytotoxicity. This mechanism is fundamentally distinct from the traditional paradigm of direct tumor cell killing. This helps explain why, while KN026-cispt-CA enhances tumor immunogenicity in immunodeficient organoids, its limited effect in immunocompetent organoids is likely due to its slower process in promoting tumor-reactive T-cell expansion, exceeding the immune cell maintenance in immunocompetent organoids. This strategy represents a conceptual advance by effectively harnessing the ‘metal immune effect’ of platinum drugs while eliminating their high-toxicity burden. Such a ‘detoxified yet immunogenic’ approach holds the potential to overcome the long-standing bottleneck of conventional chemo-immunotherapy and to establish new avenues for integrating platinum compounds into cancer immunotherapy. Moreover, it underscores the opportunity for *in vivo* redistribution and functional reprogramming of platinum agents, paving the way for innovative therapeutic strategies in precision oncology.

Our current work is based on a HER2-expressing model, relying on antibody internalization and target expression levels. Whether antibody internalization rate or target expression level influences Pt-ADC functionality remains to be further explored. In addition, future studies are needed to evaluate Pt-ADCs directed against diverse tumor cell surface antigens, with optimization of platinum payload release kinetics tailored to target expression levels and internalization rates, thereby maximizing tumor immunogenic activation and therapeutic efficacy.

Collectively, the Pt-ADC platform represents a structurally streamlined yet mechanistically versatile class of ADCs, offering broad potential for further optimization. Its modular design offers new chemical space for innovation in payload selection and conjugation chemistry, expanding the toolkit for novel ADC development. Importantly, the ‘detoxified yet immunogenic’ approach enabled by ADC construction, which uncouples tumor immunogenicity from cell death, provides a novel avenue for reimagining platinum drug development and for rationally integrating these agents with ICIs. In this context, Pt-ADCs not only redefine the therapeutic landscape of platinum-based drugs but also open new directions for the design of combination regimens in the era of precision oncology.

## MATERIALS AND METHODS

Detailed materials and methods can be found in the online supplementary file.

## Supplementary Material

nwag202_Supplemental_File

## References

[1] Sharpe AH, Pauken KE. The diverse functions of the PD1 inhibitory pathway. Nat Rev Immunol 2018; 18**:** 153–67. 10.1038/nri.2017.108 28990585

[2] Bagchi S, Yuan R, Engleman EG. Immune checkpoint inhibitors for the treatment of cancer: clinical impact and mechanisms of response and resistance. Annu Rev Pathol 2021; 16**:** 223–49. 10.1146/annurev-pathol-042020-042741 33197221

[3] Alsaafeen BH, Ali BR, Elkord E. Resistance mechanisms to immune checkpoint inhibitors: updated insights. Mol Cancer 2025; 24**:** 20. 10.1186/s12943-024-02212-7 39815294 PMC11734352

[4] Galassi C, Chan TA, Vitale I et al. The hallmarks of cancer immune evasion. Cancer Cell 2024; 42**:** 1825–63. 10.1016/j.ccell.2024.09.010 39393356

[5] McGranahan N, Rosenthal R, Hiley CT et al. Allele-specific HLA loss and immune escape in lung cancer evolution. Cell 2017; 171**:** 1259–71. 10.1016/j.cell.2017.10.001 29107330 PMC5720478

[6] Yamamoto K, Venida A, Yano J et al. Autophagy promotes immune evasion of pancreatic cancer by degrading MHC-I. Nature 2020; 581**:** 100–5. 10.1038/s41586-020-2229-5 32376951 PMC7296553

[7] Dersh D, Hollý J, Yewdell JW. A few good peptides: MHC class I-based cancer immunosurveillance and immunoevasion. Nat Rev Immunol 2021; 21**:** 116–28. 10.1038/s41577-020-0390-6 32820267

[8] Jhunjhunwala S, Hammer C, Delamarre L. Antigen presentation in cancer: insights into tumour immunogenicity and immune evasion. Nat Rev Cancer 2021; 21**:** 298–312. 10.1038/s41568-021-00339-z 33750922

[9] Chen X, Lu Q, Zhou H et al. A membrane-associated MHC-I inhibitory axis for cancer immune evasion. Cell 2023; 186**:** 3903–20. 10.1016/j.cell.2023.07.016 37557169 PMC10961051

[10] Galluzzi L, Humeau J, Buqué A et al. Immunostimulation with chemotherapy in the era of immune checkpoint inhibitors. Nat Rev Clin Oncol 2020; 17**:** 725–41. 10.1038/s41571-020-0413-z 32760014

[11] Mellman I, Chen DS, Powles T et al. The cancer-immunity cycle: indication, genotype, and immunotype. Immunity 2023; 56**:** 2188–205. 10.1016/j.immuni.2023.09.011 37820582

[12] Rodig SJ, Gusenleitner D, Jackson DG et al. MHC proteins confer differential sensitivity to CTLA-4 and PD-1 blockade in untreated metastatic melanoma. Sci Transl Med 2018; 10**:** eaar3342. 10.1126/scitranslmed.aar3342 30021886

[13] Gu SS, Zhang W, Wang X et al. Therapeutically increasing MHC-I expression potentiates immune checkpoint blockade. Cancer Discov 2021; 11**:** 1524–41. 10.1158/2159-8290.CD-20-0812 33589424 PMC8543117

[14] Ferrari V, Lo Cascio A, Melacarne A et al. Sensitizing cancer cells to immune checkpoint inhibitors by microbiota-mediated upregulation of HLA class I. Cancer Cell 2023; 41**:** 1717–30. 10.1016/j.ccell.2023.08.014 37738976

[15] Yang K, Halima A, Chan TA. Antigen presentation in cancer—mechanisms and clinical implications for immunotherapy. Nat Rev Clin Oncol 2023; 20**:** 604–23. 10.1038/s41571-023-00789-4 37328642

[16] Yu Q, Dong Y, Wang X et al. Pharmacological induction of MHC-I expression in tumor cells revitalizes T cell antitumor immunity. JCI Insight 2024; 9**:** e177788. 10.1172/jci.insight.177788 39106105 PMC11385079

[17] Kelland L . The resurgence of platinum-based cancer chemotherapy. Nat Rev Cancer 2007; 7**:** 573–84. 10.1038/nrc2167 17625587

[18] Johnstone TC, Suntharalingam K, Lippard SJ. The next generation of platinum drugs: targeted Pt(II) agents, nanoparticle delivery, and Pt(IV) prodrugs. Chem Rev 2016; 116**:** 3436–86. 10.1021/acs.chemrev.5b00597 26865551 PMC4792284

[19] Rottenberg S, Disler C, Perego P. The rediscovery of platinum-based cancer therapy. Nat Rev Cancer 2021; 21**:** 37–50. 10.1038/s41568-020-00308-y 33128031

[20] Wang X, Guo Z. Targeting and delivery of platinum-based anticancer drugs. Chem Soc Rev 2013; 42**:** 202–24. 10.1039/C2CS35259A 23042411

[21] Peng K, Liang B-B, Liu W et al. What blocks more anticancer platinum complexes from experiment to clinic: major problems and potential strategies from drug design perspectives. Coord Chem Rev 2021; 449**:** 214210. 10.1016/j.ccr.2021.214210

[22] Huang R, Sun Y, Zhang X-y et al. Biological evaluation of a novel Herceptin–platinum (II) conjugate for efficient and cancer cell specific delivery. Biomed Pharmacother 2015; 73**:** 116–22. 10.1016/j.biopha.2015.05.013 26211591

[23] Fu Q, Zhang S, Shen S et al. Radiotherapy-triggered reduction of platinum-based chemotherapeutic prodrugs in tumours. Nat Biomed Eng 2024; 8**:** 1425–35. 10.1038/s41551-024-01239-x 39025943

[24] Yin X, Zhuang Y, Song H et al. Antibody–platinum (IV) prodrugs conjugates for targeted treatment of cutaneous squamous cell carcinoma. J Pharm Anal 2024; 14**:** 389–400. 10.1016/j.jpha.2023.11.002 38618248 PMC11010626

[25] Huang T, Huang W-Q, Huang G-F et al. Pincer-type Pt(II)-NHC antibody–drug conjugate for HER-2-targeted chemoimmunotherapy. Adv Healthc Mater 2025; 14**:** 2403449. 10.1002/adhm.202403449 39950551 PMC11973945

[26] Tesniere A, Schlemmer F, Boige V et al. Immunogenic death of colon cancer cells treated with oxaliplatin. Oncogene 2010; 29**:** 482–91. 10.1038/onc.2009.356 19881547

[27] Tham MJR, Babak MV, Ang WH. PlatinER: a highly potent anticancer platinum(II) complex that induces endoplasmic reticulum stress driven immunogenic cell death. Angew Chem Int Ed 2020; 59**:** 19070–8. 10.1002/anie.202008604 32716112

[28] Deng Z, Li H, Chen S et al. Near-infrared-activated anticancer platinum(IV) complexes directly photooxidize biomolecules in an oxygen-independent manner. Nat Chem 2023; 15**:** 930–9. 10.1038/s41557-023-01242-w 37353602

[29] Yang T, Zhang S, Yuan H et al. Platinum-based TREM2 inhibitor suppresses tumors by remodeling the immunosuppressive microenvironment. Angew Chem Int Ed 2023; 62**:** e202213337. 10.1002/anie.202213337 36259513

[30] Liu L-Y, Ma T-Z, Zeng Y-L et al. Organic-platinum hybrids for covalent binding of G-quadruplexes: structural basis and application to cancer immunotherapy. Angew Chem Int Ed 2023; 62**:** e202305645. 10.1002/anie.202305645 37464955

[31] Garassino MC, Gadgeel S, Speranza G et al. Pembrolizumab plus pemetrexed and platinum in nonsquamous non–small-cell lung cancer: 5-year outcomes from the phase 3 KEYNOTE-189 study. J Clin Oncol 2023; 41**:** 1992–8. 10.1200/JCO.22.01989 36809080 PMC10082311

[32] Akinboro O, Drezner N, Amatya A et al. US Food and Drug Administration approval summary: nivolumab plus platinum-doublet chemotherapy for the neoadjuvant treatment of patients with resectable non-small-cell lung cancer. J Clin Oncol 2023; 41**:** 3249–59. 10.1200/JCO.22.02509 37141544 PMC10256356

[33] Reck M, Ciuleanu T-E, Schenker M et al. Five-year outcomes with first-line nivolumab plus ipilimumab with 2 cycles of chemotherapy versus 4 cycles of chemotherapy alone in patients with metastatic non-small cell lung cancer in the randomized CheckMate 9LA trial. Eur J Cancer 2024; 211**:** 114296. 10.1016/j.ejca.2024.114296 39270380

[34] Grimaldi A, Cammarata I, Martire C et al. Combination of chemotherapy and PD-1 blockade induces T cell responses to tumor non-mutated neoantigens. Commun Biol 2020; 3**:** 85. 10.1038/s42003-020-0811-x 32099064 PMC7042341

[35] Anker J, Pal SK, Kim-Schulze S et al. Antitumor immunity as the basis for durable disease-free treatment-free survival in patients with metastatic urothelial cancer. J ImmunoTher Cancer 2023; 11**:** e007613. 10.1136/jitc-2023-007613 37607770 PMC10445357

[36] Xue Q, Yu W, Li JP et al. Revealing the nature of Pt-based immunotherapy through the lens of neoantigens in cancer. Sci Bull 2024; 69**:** 2314–8. 10.1016/j.scib.2024.04.032 38670854

[37] Galluzzi L, Guilbaud E, Schmidt D et al. Targeting immunogenic cell stress and death for cancer therapy. Nat Rev Drug Discov 2024; 23**:** 445–60. 10.1038/s41573-024-00920-9 38622310 PMC11153000

[38] Mo H, Yu Y, Sun X et al. Metronomic chemotherapy plus anti-PD-1 in metastatic breast cancer: a Bayesian adaptive randomized phase 2 trial. Nat Med 2024; 30**:** 2528–39. 10.1038/s41591-024-03088-2 38969879

[39] Chen L, Li H, Zhang H et al. Camrelizumab vs placebo in combination with chemotherapy as neoadjuvant treatment in patients with early or locally advanced triple-negative breast cancer: the CamRelief randomized clinical trial. JAMA 2025; 333**:** 673–81. 10.1001/jama.2024.23560 39671272 PMC11862970

[40] Burtness B, Harrington KJ, Greil R et al. Pembrolizumab alone or with chemotherapy versus cetuximab with chemotherapy for recurrent or metastatic squamous cell carcinoma of the head and neck (KEYNOTE-048): a randomised, open-label, phase 3 study. The Lancet 2019; 394**:** 1915–28. 10.1016/S0140-6736(19)32591-7 31679945

[41] Yang Y, Song Z, Tian T et al. Trimming crystallizable fragment (Fc) glycans enables the direct enzymatic transfer of biomacromolecules to antibodies as therapeutics. Angew Chem Int Ed 2023; 62**:** e202308174. 10.1002/anie.202308174 37438983

[42] Zhang J, Ji D, Cai L et al. First-in-human HER2-targeted bispecific antibody KN026 for the treatment of patients with HER2-positive metastatic breast cancer: results from a phase I study. Clin Cancer Res 2022; 28**:** 618–28. 10.1158/1078-0432.CCR-21-2827 34844975

[43] Xu Z, Tang WK, Zhou Q et al. On the hydrolytic stability of unsymmetric platinum(iv) anticancer prodrugs containing axial halogens. Inorg Chem Front 2021; 8**:** 3794–802. 10.1039/D1QI00208B

[44] Ng MSF, Kwok I, Tan L et al. Deterministic reprogramming of neutrophils within tumors. Science 2024; 383**:** eadf6493. 10.1126/science.adf6493 38207030 PMC11087151

[45] Zhao Z, Zhang S, Jiang N et al. Patient-derived immunocompetent tumor organoids: a platform for chemotherapy evaluation in the context of T-cell recognition. Angew Chem Int Ed 2024; 63**:** e202317613. 10.1002/anie.202317613 38195970

[46] Huang Q, Wu X, Wang Z et al. The primordial differentiation of tumor-specific memory CD8^+^ T cells as bona fide responders to PD-1/PD-L1 blockade in draining lymph nodes. Cell 2022; 185**:** 4049–66. 10.1016/j.cell.2022.09.020 36208623

[47] Wijesinghe SKM, Rausch L, Gabriel SS et al. Lymph-node-derived stem-like but not tumor-tissue-resident CD8^+^ T cells fuel anticancer immunity. Nat Immunol 2025; 26**:** 1367–83. 10.1038/s41590-025-02219-2 40730900

[48] Xu Z, Wang Z, Deng Z et al. Recent advances in the synthesis, stability, and activation of platinum(IV) anticancer prodrugs. Coord Chem Rev 2021; 442**:** 213991. 10.1016/j.ccr.2021.213991

