# Supplementary material for: Uncoupling tumor immunogenicity from cell death with platinum(IV)–antibody conjugates
Source: Natl Sci Rev. 2026 Apr 2;13(10):nwag202. doi: 10.1093/nsr/nwag202 (PMC13234940; doi:10.1093/nsr/nwag202)
Supplement: nwag202_Supplemental_File [file nwag202_supplemental_file.pdf]

Supplementary information

Uncoupling tumor immunogenicity from cell death with platinum(IV)-antibody conjugates

Liu-Yi Liu[1,†], Wenhao Yu[1,†], Yilong Liu[1], Yang Yang[1], Qiuyang Wei[1], Zihan Zhao[2], Rong Yang[2], Jie P. Li[1,*], Zijian Guo[1,*]

[1]State Key Laboratory of Coordination Chemistry, Chemistry and Biomedicine Innovation Center (ChemBIC), School of Chemistry and Chemical Engineering, Nanjing University, Nanjing 210023, China
[2]Department of Urology, Nanjing Drum Tower Hospital, Affiliated Hospital of Medical School, Nanjing University, Nanjing 210008, China
*Corresponding authors. E-mails: jieli@nju.edu.cn; zguo@nju.edu.cn
†Equally contributed to this work

Table of Contents

Supplementary Methods 3
Supplementary Figures 9
Supplementary Tables 32
Chemical Synthesis and Characterization 33
Chemical Synthesis 33
NMR and MS spectra 38
Supplementary References 47

Supplementary Methods

LC-MS analysis of antibodies and Pt-ADCs

Liquid chromatography-mass spectrometry (LC-MS) was performed using a Xevo G2-S TOF mass spectrometer (Waters) coupled to an Acquity UPLC system (Waters) equipped with a Protein BEH C4 column (1.7 μm, 2.1 × 50 mm). The mobile phases consisted of water with 0.1% formic acid (solvent A) and acetonitrile with 0.1% formic acid (solvent B), delivered at a flow rate of 0.5 mL min^{-1}. The elution was carried out with the following gradient: 95% solvent A (isocratic, 2 min), 95–10% solvent A (linear, 4 min), 10% solvent A (isocratic, 1 min), 10–95% solvent A (linear, 1 min), and 95% solvent A (isocratic, 2 min). Main chromatographic peaks were selected for integration and subsequent ion series analysis.

Reductive behavior of Pt-ADCs

Pt-ADCs were incubated at 37 °C in PBS (pH 7.4) containing varying equivalents of ascorbic acid (ASA; 25, 100, or 1,000 equiv.) for different time intervals. Samples were analyzed by LC-MS to assess release behavior. To investigate pH-dependent stability, Pt-ADCs were also incubated in PBS (adjusted to pH 6.4 or 5.4 using HCl) containing 1,000 equiv. of ASA, followed by LC-MS analysis. To evaluate reduction under biologically relevant conditions, Pt-ADCs were incubated at 37 °C in the following media: PBS containing 10% fetal bovine serum (FBS), PBS containing 50% human serum, in vitro cell culture media (10% FBS in RPMI 1640 medium with CT26 cell culture), and supernatant collected from in vivo CT26 tumors. The tumor supernatant was collected by triple centrifugation at 450 × *g* to remove cells and debris. LC-MS was used to monitor Pt-ADC integrity over time in all conditions.

To characterize the species of Pt payload after reduction, samples were prepared by reacting Cispt-CA-N_3 (25 mM) with 5 equiv. of ASA in a 5:1 d_6-DMSO:D_2O solution. The time course of the reaction was monitored by ^{195}Pt-NMR after incubating the samples at 37 °C for 0, 3, 6, 12, and 24 h in the dark.

Cell culture

Cell lines were all purchased from The Chinese National Infrastructure of Cell Line Resource unless otherwise specified. CT26-HER2 cells were constructed by lentiviral infection to express HER2 on CT26 cells. CT26-cHER2 cells were constructed by lentiviral infection to express cHER2 on CT26 cells. NCI-N87, A549, CT26 and their derived-cells were maintained in RPMI-1640 medium (Gibco BRL) supplemented with 10% fetal bovine serum (FBS; Gibco BRL), 100 μg/mL streptomycin (Gibco BRL), and 100 U/mL penicillin (Gibco BRL). SK-OV-3 were maintained in DMEM medium (Gibco BRL) supplemented with 10% fetal bovine serum (FBS; Gibco BRL), 100 μg/mL streptomycin (Gibco BRL), and 100 U/mL penicillin (Gibco BRL). Cultures were incubated at 37 °C in a humidified atmosphere containing 5% CO_2 and 95% air.

Cell-binding assay

Pt-ADCs or trastuzumab (0.1 mg/mL) was incubated with HER2-positive SK-OV-3 cells at 37 °C for 30 min. After incubation, unbound antibodies were removed by washing, and the cells were stained with PE-conjugated anti-human IgG Fc (BioLegend) at 4 °C for 30 min. Samples were analyzed by flow cytometry, and antibody binding was analyzed by calculating the fold change in median fluorescence intensity (MFI) of PE-conjugated anti-human IgG Fc staining relative to

control.

Antibody internalization assay

Antibody or Pt-ADC was incubated with CT26-HER2 or CT26 cells for either 5 h or 24 h. Internalization was then assessed using a pHrodo™ Antibody Labeling Kits (Invitrogen™) according to the manufacturer's instructions. The pHrodo dye is non-fluorescent at neutral extracellular pH and becomes brightly fluorescent only in the acidic environment of endosomes or lysosomes. Fluorescence intensity was measured by flow cytometry to quantify antibody internalization.

Cell viability assay

The cytotoxicity of Pt-ADCs or small-molecule platinum agents against various cell lines was assessed using the Cell Counting Kit-8 (CCK-8) assay. Cells were seeded in 96-well plates and allowed to adhere overnight before treatment with serial dilutions of Pt-ADCs or small-molecule platinum agents. For Pt-ADCs, after 95 h of incubation, 10% CCK-8 reagent was added to each well, followed by a 1 h incubation. For small-molecule platinum agents, after 71 h of incubation, 10% CCK-8 reagent was added to each well, followed by a 1 h incubation. Absorbance at 450 nm was then measured using a microplate reader. All experiments were performed in triplicate.

Effect on MHC-I expression

A549 and SK-OV-3 cells were seeded into 96-well plates and incubated overnight. Pt-ADCs at varying concentrations were added to the culture medium and incubated with the cells for 96 h. Cells were then harvested and stained on ice for 30 min with FITC-conjugated anti-human HLA-A,B,C antibody (BioLegend), followed by three washes with FACS buffer. MHC-I expression was analyzed by flow cytometry.

For small-molecule platinum agents, CT26-HER2 cells were seeded into 96-well plates and incubated overnight. Small-molecule platinum agents at indicated concentration were added to the culture medium and incubated with the cells for 48 h. Cells were then harvested and stained on ice for 30 min with PE-conjugated anti-mouse H-2Kd (BioLegend), followed by three washes with FACS buffer. MHC-I expression was analyzed by flow cytometry. Additionally, to analyze the impact of small-molecule platinum agents on antigen processing and presentation at the transcriptomic level, CT26-HER2 cells were treated with small-molecule platinum agents (0.2 μM) for 48 h. After treatment, the cells were collected, washed three times with PBS, lysed, and processed for mRNA extraction, followed by bulk RNA sequencing.

Western blot

A549 cells were seeded in 6-well plates and allowed to adhere for 24 h, followed by treatment with cisplatin (0.2 μM) or Pt-2CA (0.2 μM) for 48 h. Cells were then harvested, washed three times with ice-cold PBS, and lysed on ice for 30 min in RIPA buffer supplemented with PMSF (100 μg/mL). Lysates were clarified by centrifugation at 4 °C, and protein concentrations were determined using a BCA assay. Equal amounts of protein were mixed with loading buffer, boiled at 100 °C for 10 min to ensure complete denaturation, and subjected to SDS–PAGE. Immunoblotting was subsequently performed according to the manufacturer's instructions.

ROS detection

Intracellular ROS levels were quantified using the DCFH-DA assay. A549 cells were seeded in 96-well plates and allowed to adhere overnight. Cells were then treated with cisplatin (0.2 μM) or Pt-2CA (0.2 μM) for 48 h. Following treatment, cells were washed twice with PBS and incubated with 10 μM DCFH-DA in serum-free RPMI 1640 medium for 30 min at 37 °C in the dark. After two additional washes with PBS to remove excess probe, fluorescence intensity was measured by flow cytometry using the FITC channel.

Quantification of platinum by ICP-MS

In vitro analysis: To assess the intracellular distribution of platinum following Pt-ADC and small-molecule platinum agent treatment, CT26-HER2 cells were incubated for 48 h with Pt-ADCs (400 μg/mL) or small-molecule platinum agents (0.5 μM). After incubation, nuclear and cytoplasmic fractions were separated using a commercial kit (C500009, Sangon Biotech) according to the manufacturer's instructions. Each fraction was then digested thoroughly with concentrated nitric acid, hydrochloric acid, and hydrogen peroxide, brought to a final volume of 2 mL, and analyzed by inductively coupled plasma mass spectrometry (ICP-MS) to determine the absolute platinum content.

In vivo analysis: To examine the biodistribution of platinum after Pt-ADC and cisplatin treatment in mice, CT26-HER2 cells were subcutaneously inoculated into the right flank of female BALB/c mice. When tumors reached approximately 300 mm^3, mice were treated with KN026-cispt-OH (10 mg/kg, intraperitoneally) or cisplatin (1 mg/kg, intravenously). After treatment for 48 h, tumors and major organs were harvested, weighed, and digested with concentrated nitric acid, hydrochloric acid, and hydrogen peroxide. Digests were adjusted to a final volume of 2 mL, and platinum content was quantified using ICP-MS.

In vivo antitumor assay

BALB/c female mice (6-8 weeks old) and BALB/c nude female mice (6-8 weeks old) were obtained from GemPharmatech (Nanjing, Jiangsu). All mice were specific pathogen-free (SPF) and were bred and maintained under standard conditions. The animal use protocol was approved by the Animal Ethics and Welfare Committee (IACUC-2412021; IACUC-2410008).

For antitumor assay treated by small-molecule platinum agents, CT26-cHER2 cells were subcutaneously inoculated into the right flank of BALB/c female mice on day -10. Depending on the treatment group, mice were administered small-molecule platinum agents (1 mg Pt/kg) via intravenous injection on days 0, 4, 8, 12 and 16. In the combination treatment group, αPD-1 (5 mg/kg, Nofazinlimab) was administered intraperitoneally on days 2, 9, and 16. On day 18, tumors were harvested for immune flow cytometry analysis.

To compare the immunomodulatory effects of cinnamic acid (CA) and Pt-2CA, CT26-HER2 cells were subcutaneously implanted into the right flank of BALB/c female mice on day -10. Mice received intravenous administration on days 0 and 3 (0.5 mg CA/kg or 1 mg Pt-2CA/kg, with equivalent cinnamic acid doses in both groups). Tumors were subsequently harvested and analyzed by flow cytometry to assess tumor-infiltrating immune cell populations.

For antitumor assay treated by Pt-ADCs, CT26-HER2 cells were subcutaneously inoculated into the right flank of BALB/c female mice on day -10. Depending on the treatment group, mice were administered either KN026 or KN026-cispt-CA (10 mg/kg) via intraperitoneal injection on

days 0, 7, and 13. In the combination treatment group, αPD-1 (5 mg/kg, Nofazinlimab) was administered intraperitoneally on days 2, 9, and 15. On day 17, tumors were harvested for immune flow cytometry analysis. Additionally, following this treatment of CT26-HER2 tumors on day 17, live tumor cells and immune cells were sorted based on Zombie Aqua™ and PE-CD45 staining. These cells were then subjected to single-cell RNA sequencing. Immune cells were further analyzed using TCR sequencing.

For large tumor model treated by Pt-ADCs, CT26-HER2 cells were subcutaneously inoculated into the right flank of BALB/c female mice on day -14. When tumors reached approximately 300 mm^3, treatments were administered according to the designated experimental groups. Depending on the treatment group, mice were administered either KN026-cispt-OH or KN026-cispt-CA (10 mg/kg) via intraperitoneal injection on days 0 and 7. In the combination treatment group, αPD-1 (5 mg/kg, Nofazinlimab) was administered intraperitoneally on days 2 and 9. On day 13, tumors were harvested for immune flow cytometry analysis.

To evaluate the therapeutic advantages of the Pt-ADC delivery strategy, CT26-HER2 cells were implanted subcutaneously into BALB/c female mice. Depending on the treatment group, mice received either KN026 (10 mg/kg, intraperitoneal) combined with Pt-2CA (0.15 mg/kg, intravenous), or KN026-cispt-CA (10 mg/kg, intraperitoneal) on days 0 and 7. In the combination therapy groups, αPD-1 (5 mg/kg, Nofazinlimab) was administered intraperitoneally on days 2 and 9. Tumors were collected on day 11 for subsequent immune flow cytometric analysis.

For the NCI-N87 human-derived gastric cancer cell subcutaneous xenograft model treated by small-molecule platinum agents or Pt-ADCs, NCI-N87 cells were subcutaneously inoculated into the right dorsal flank of BALB/c nude female mice. When tumors reached approximately 170 mm^3, treatments were administered according to the designated experimental groups. Mice were treated with either Herceptin or H-cispt-OH (10 mg/kg) via intraperitoneal injection on days 0, 7, and 14, or received cisplatin (1 mg/kg or 0.078 mg/kg) via intravenous injection on days 0, 7, and 14, depending on the experimental group.

After the initiation of treatment, the tumor size in mice was measured regularly. Tumor volume (mm^3) = 1/2 × (a × b^2), where 'a' represents the longest diameter and 'b' represents the shortest diameter.

To evaluate the systemic toxicity of Pt-ADC, mice were administered KN026-cispt-CA (10 mg/kg) via intraperitoneal injection or cisplatin (1 mg/kg) via intravenous injection on days 0, 5, 10, 15, and 20. On day 25, the major organs of the mice were collected for pathological analysis, and serum samples were collected to assess biochemical markers.

Effect on HER2-positive PDOs

The study related to human clinical samples was approved by the Ethics Committee of Nanjing Drum Tower Hospital in accordance with the Declaration of Helsinki (2024-884-02, 2024-884-03). All patients provided informed consent. HER2-positive immunodeficient patient-derived organoids (PDOs) were exposed to Pt-ADCs to evaluate their cytotoxic and transcriptional responses. PDOs were treated with Pt-ADCs at a concentration of 300 μg/mL for 84 h. Post-treatment, PDO viability was assessed. The cells were collected, washed three times with PBS, lysed, and processed for mRNA extraction, followed by bulk RNA sequencing. For HER2-positive immunocompetent PDO model, tumor samples were collected and cultured to generate tumor-like cell clusters retaining immune components[1-2]. After Pt-ADC (300 μg/mL) and anti-

PD-1 (10 μg/mL, Nofazinlimab) treatment for 7 days, tumor-like cell clusters viability was measured.

Flow cytometric analysis

Flow cytometry was performed using a BD Aria III. The Zombie Fixable Viability Kit was used to distinguish live cells from dead cells. All antibodies for flow cytometry, along with the mouse Fc-blocker (TruStain fcX™ anti-mouse CD16/32) and Zombie Aqua™, were sourced from BioLegend. Data were analyzed using FlowJo v10.8.1. In all flow cytometric analyses, MFI refers to median fluorescence intensity.

Bulk RNA-seq analysis

RNA-seq libraries were constructed using AccuraCode® HTP One Step RNAseq Kit (Singleron Biotechnologies, Nanjing, China), according to the manufacturer's protocol. RNA-seq libraries were generated via reverse transcription and PCR amplification. The libraries were quality-checked and sequenced using the Illumina NovaSeq 6000 S4 PE150 platform. Raw sequencing reads were processed using AccuraCode pipeline (v1.2.0) with default parameters. DEGs were analyzed using the DESeq2 R package. For PDO samples, GO enrichment analysis was conducted on the upregulated genes in the specified group compared to another, using the org.Hs.eg.db annotation database.

Single-cell RNA-seq and TCR-seq analysis

Single-cell RNA-seq libraries were constructed using the GEXSCOPE Single-Cell RNA Library Kit (Singleron Biotechnologies, Nanjing, China), following the manufacturer's protocol. After quality control, individual libraries were diluted and pooled for sequencing on an Illumina NovaSeq 6000 S4 PE150 platform. Raw sequencing reads were processed using the CeleScope pipeline with default parameters. Single-cell TCR-seq libraries were constructed using the sCircle®Single Cell Full Length Immuno-TCR Library Kit (Singleron Biotechnologies, Nanjing, China), following the manufacturer's protocol. After filtering out cells with low-quality gene expression profiles, unsupervised clustering and cell annotation were performed on the single-cell RNA sequencing data. Subpopulations of interest were then selected for further analysis. For the neutrophil subpopulations, scoring was performed using the AddModuleScore function based on gene sets for T1, T2, and T3 neutrophil types as reported[3]. Unsupervised clustering and cell annotation were re-applied to the T cell and NK cell subpopulations. For T cells, the transcriptome data were integrated with the corresponding T-cell receptor (TCR) repertoire to analyze the clonal distribution of TCRs, using scRepertoire package. GO enrichment analysis was performed on the upregulated genes in the specified group relative to another, using the clusterProfiler package and the org.Mm.eg.db annotation database.

Raw sequencing data uploaded and deposited into the CNGB Sequence Archive (CNSA) of the China National GeneBank Database (CNGBdb) with accession number CNSA: CNP0009076.

Statistical analysis

Statistical analyses were conducted using GraphPad Prism software (version 8.0.2). Statistics were determined using unpaired Student's t-test, one-way ANOVA with Tukey's multiple

comparisons test, and two-way ANOVA with Tukey's multiple comparisons test. Data are presented as mean ± standard deviation. *$P < 0.05$; ** $P < 0.01$; *** $P < 0.001$; **** $P < 0.0001$.

Supplementary Figures

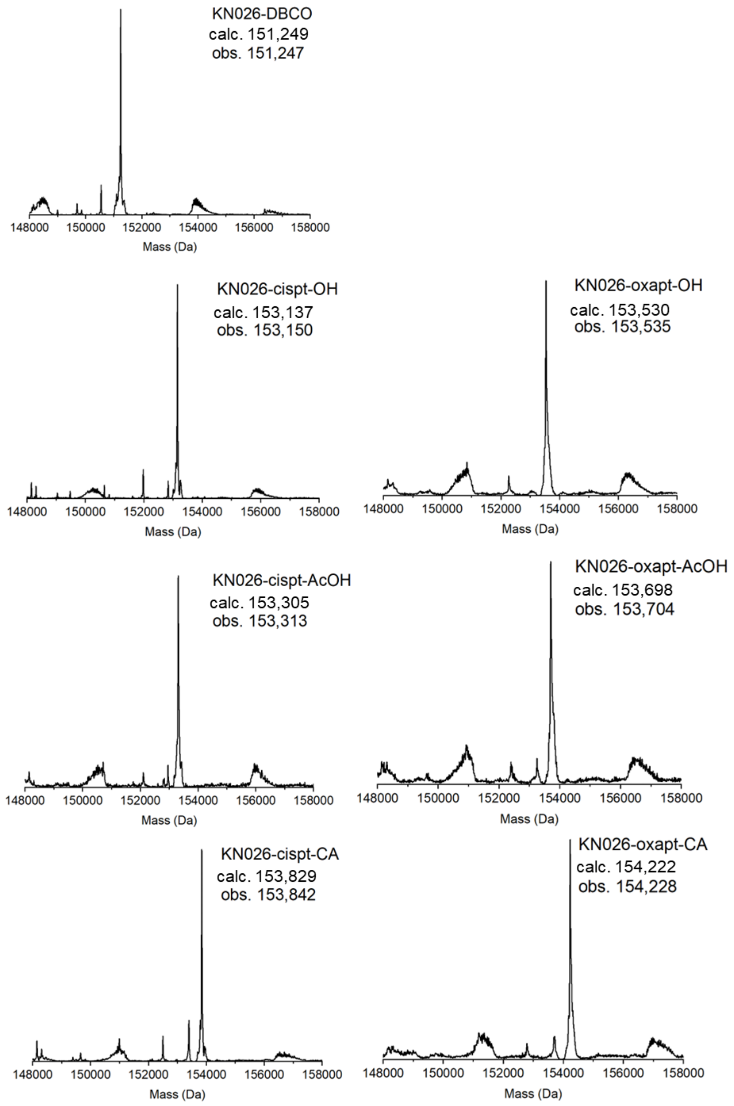

Figure S1. LC-MS analysis of KN026-based Pt-ADCs.

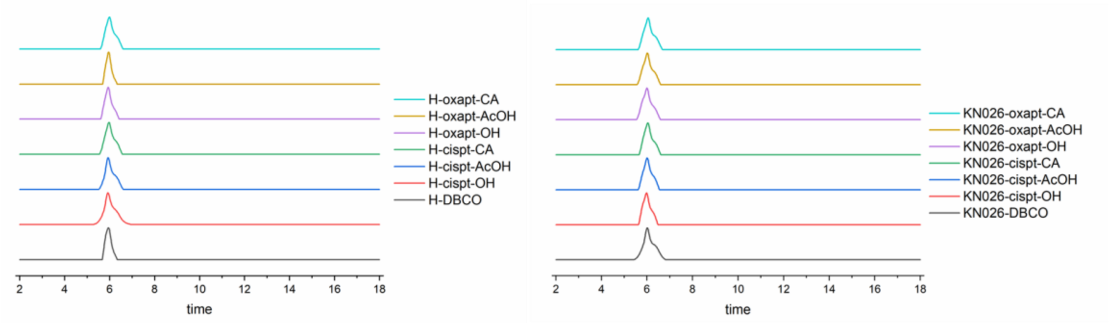

Figure S2. Corresponding LC trace for the LC-MS analysis of Pt-ADCs.

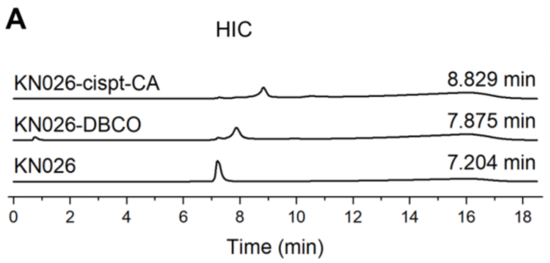

Figure S3. (A) HIC profile and (B) SEC profile of KN026-cispt-CA. SEC analysis demonstrated that, compared with KN026-DBCO, conjugation of the platinum payload did not appreciably alter the aggregation state of the antibody.

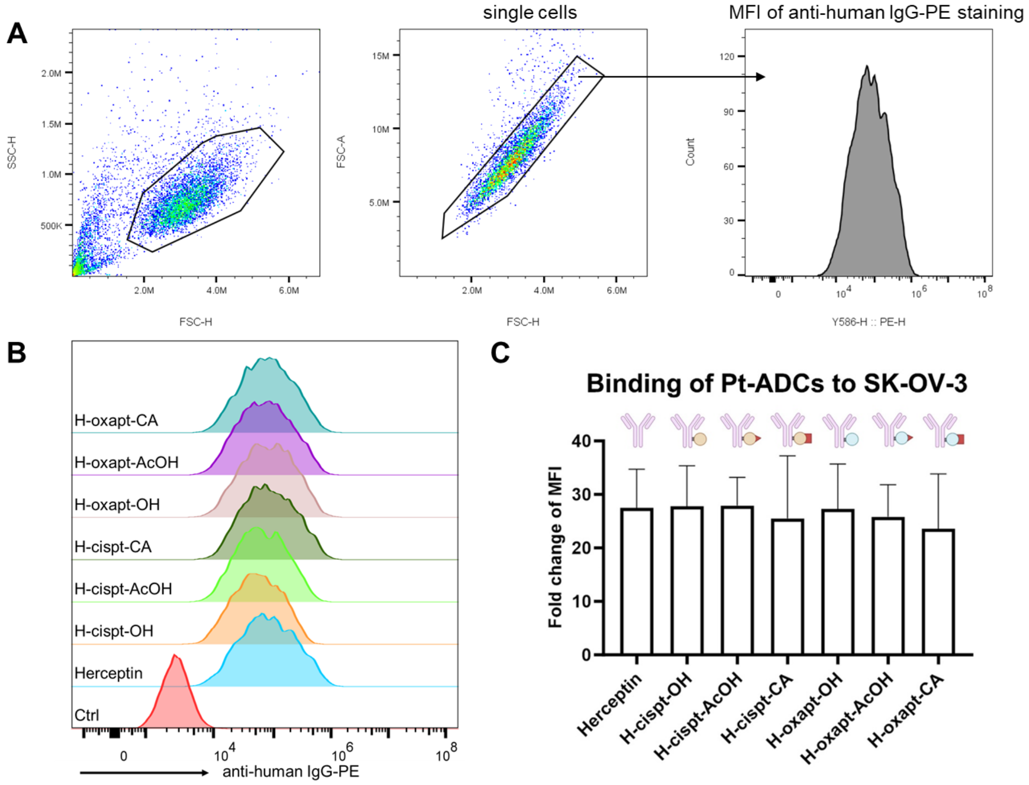

Figure S4. (A) Flow cytometric gating strategy for analyzing antibody binding to the cell surface. (B) Median fluorescence intensity (MFI) and (C) Fold change in MFI of anti-human IgG-PE staining in SK-OV-3 cells after 0.5-h incubation with Herceptin or Pt-ADCs, indicating antibody binding to the cell surface. Data are mean ± s.d. (n ⩾ 3).

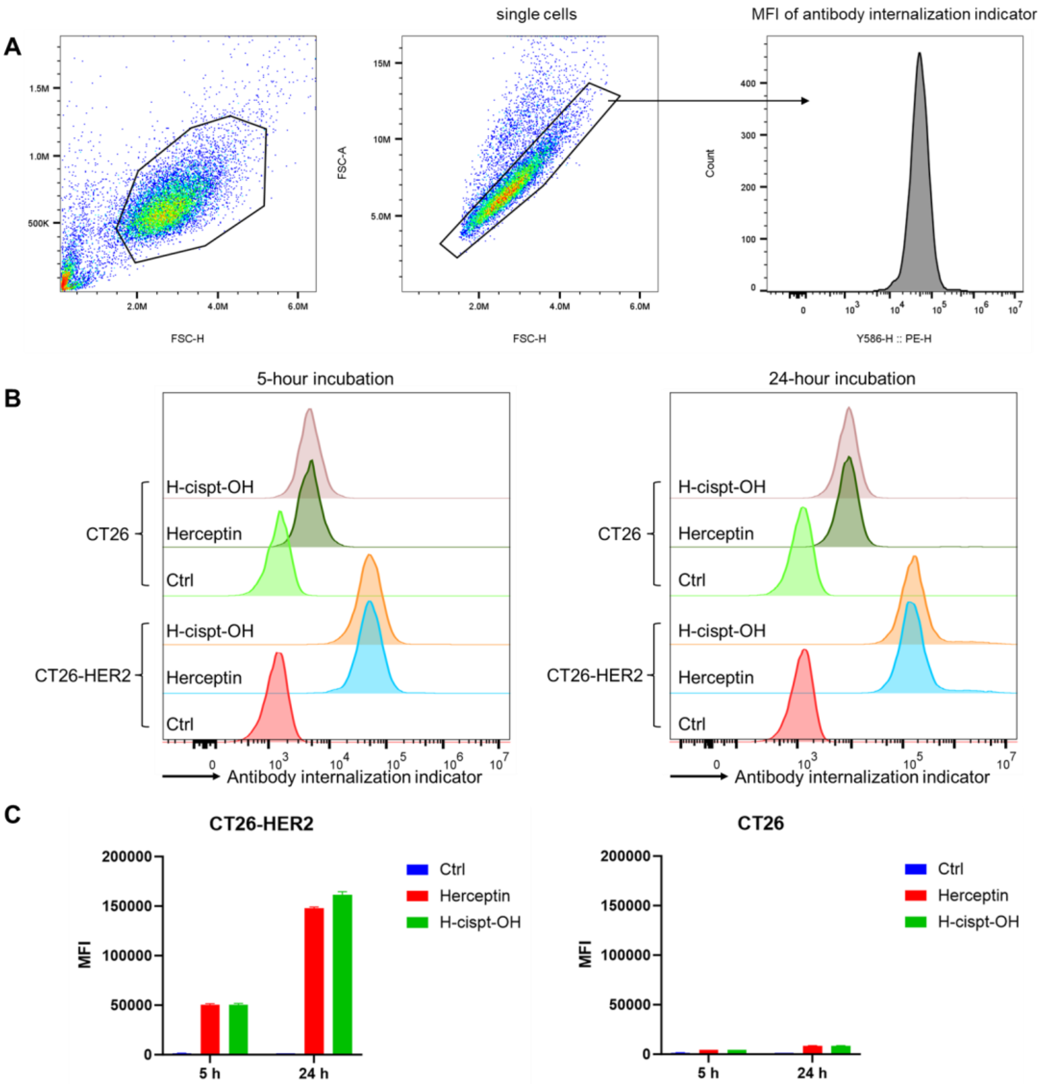

Figure S5. (A) Flow cytometric gating strategy for analyzing antibody internalization. (B-C) MFI of antibody internalization indicator in CT26-HER2 and CT26 cells after 5-h or 24-h incubation with Herceptin or Pt-ADCs, indicating Pt-ADC internalization. Compared with 5 h of incubation, MFI values further increased at 24 h, indicating sustained internalization and arguing against rapid efflux of the conjugates.

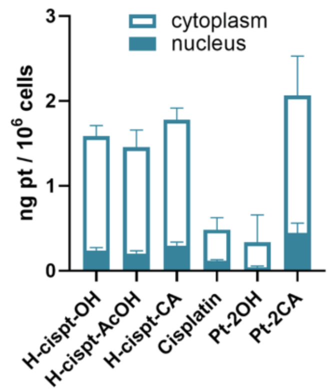

Figure S6. Intracellular platinum levels in CT26-HER2 cells measured by ICP-MS after 48 h incubation with Pt-ADCs (400 μg/mL) and small-molecule platinum agents (0.5 μM).

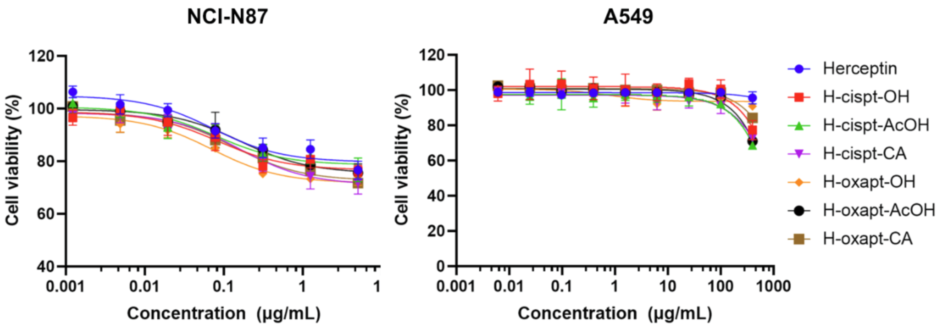

Figure S7. Cytotoxicity of Pt-ADCs against HER2^{high} NCI-N87 cells and HER2^{low} A549 cells for 96 h.

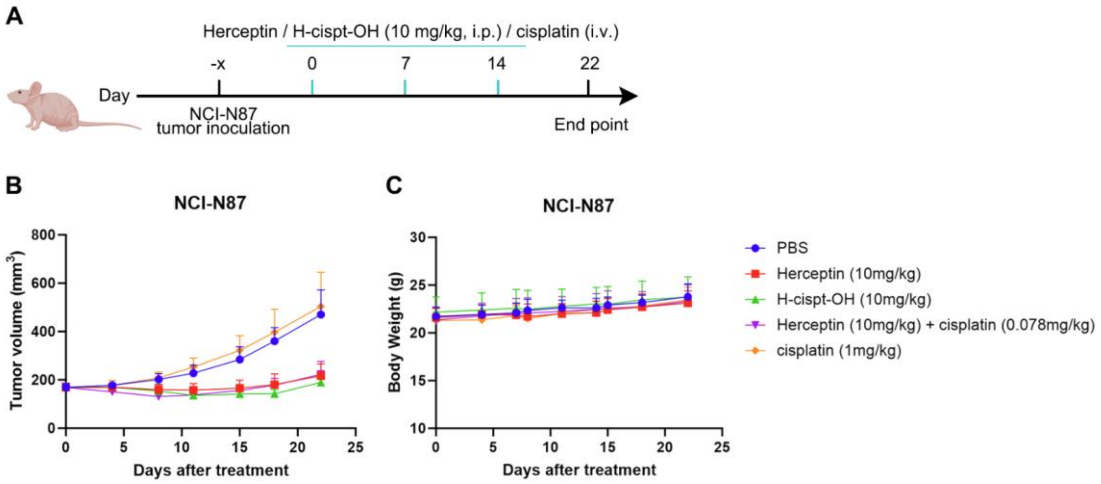

Figure S8. (A) Schematic representation of the treatment regimen using Pt-ADC or cisplatin in the NCI-N87 human-derived gastric cancer cell subcutaneous xenograft model in BALB/c nude female mice. (B) Tumor volume changes during the course of treatment. (C) Body weight changes during the course of treatment. The platinum content in the cisplatin (0.078 mg/kg) dose is equivalent to that in the H-cispt-OH (10 mg/kg) dose.

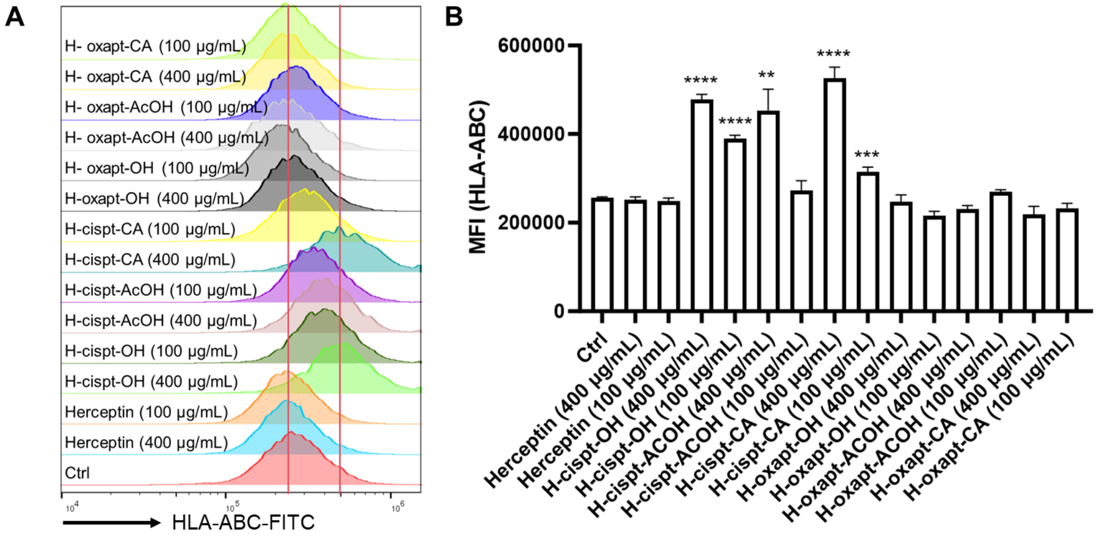

Figure S9. (A-B) Flow cytometric analysis of MHC-I (HLA-ABC) expression induction in A549 cells following Pt-ADC treatment for 96 h. Data are mean ± s.d. (n ⩾ 3). *P < 0.05; ** P < 0.01; *** P < 0.001; **** P < 0.0001, compare to control.

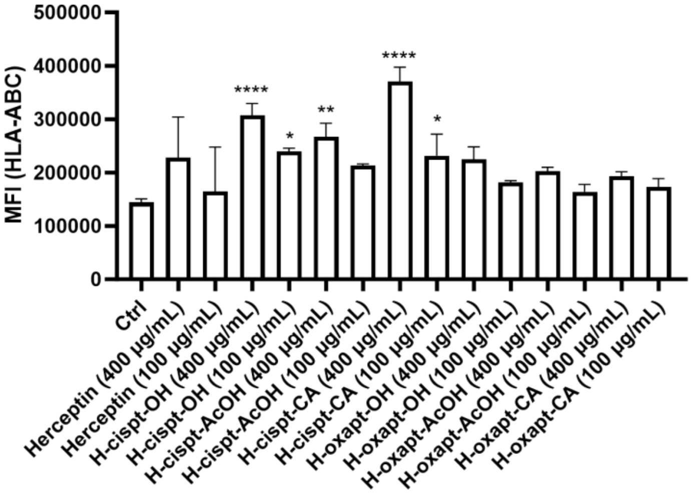

Figure S10. Flow cytometric analysis of MHC-I (HLA-ABC) expression induction in SK-OV-3 cells following Pt-ADC treatment for 96 h. Data are mean ± s.d. (n ⩾ 3). *P < 0.05; ** P < 0.01; *** P < 0.001; **** P < 0.0001, compare to control.

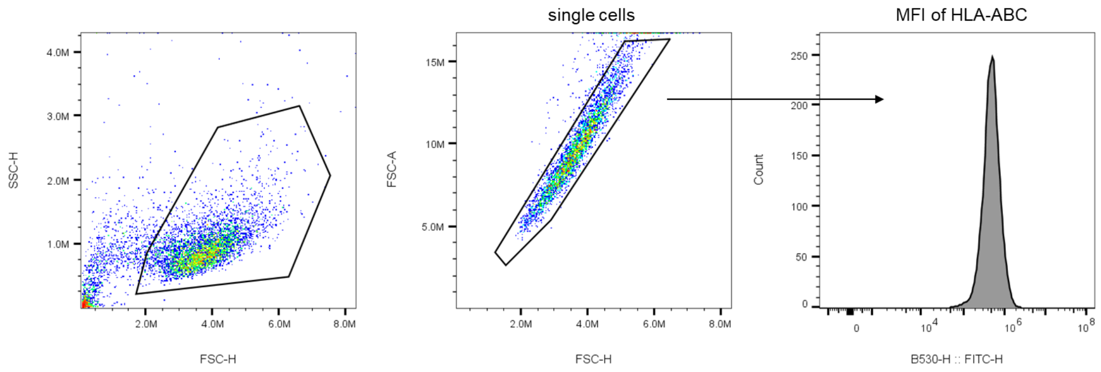

Figure S11. Flow cytometric gating strategy for analyzing MHC-I (HLA-ABC) expression in Figure S9-S10.

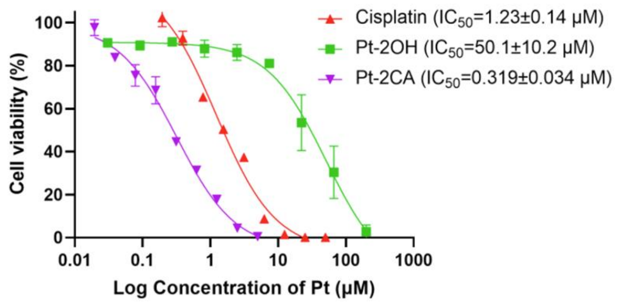

Figure S12. Viability of CT26-HER2 cells after 72 h treatment with small-molecule platinum drugs.

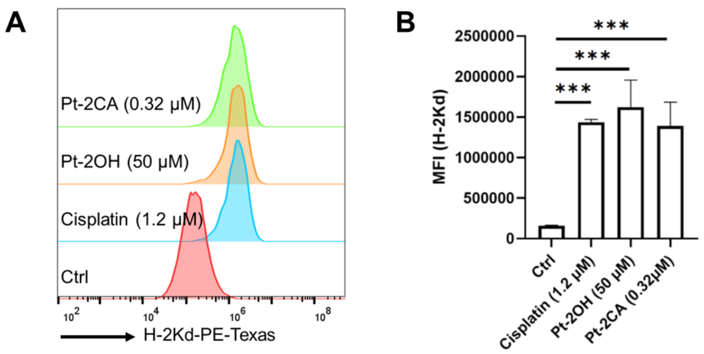

Figure S13. (A-B) Flow cytometric analysis of MHC-I (H-2Kd) expression induction in CT26-HER2 cells after 48 h treatment with small-molecule platinum drugs. Data are mean ± s.d. (n ⩾ 3). *$P < 0.05$; ** $P < 0.01$; *** $P < 0.001$; **** $P < 0.0001$, compare to control.

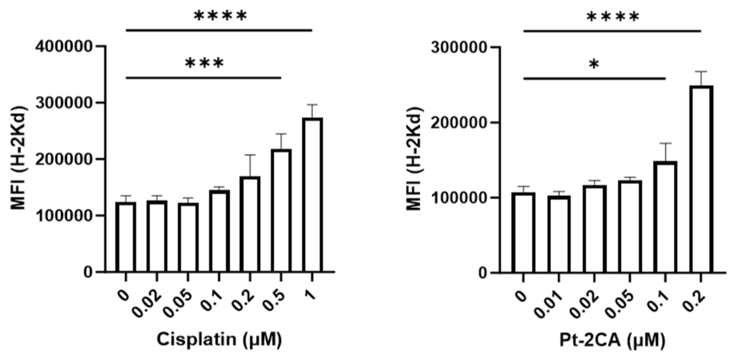

Figure S14. Flow cytometric analysis of MHC-I (H-2Kd) expression induction in CT26-HER2 cells after 48 h treatment with cisplatin and Pt-2CA at different concentrations. Data are mean ± s.d. (n ⩾ 3). **P* < 0.05; ** *P* < 0.01; *** *P* < 0.001; **** *P* < 0.0001, compare to control.

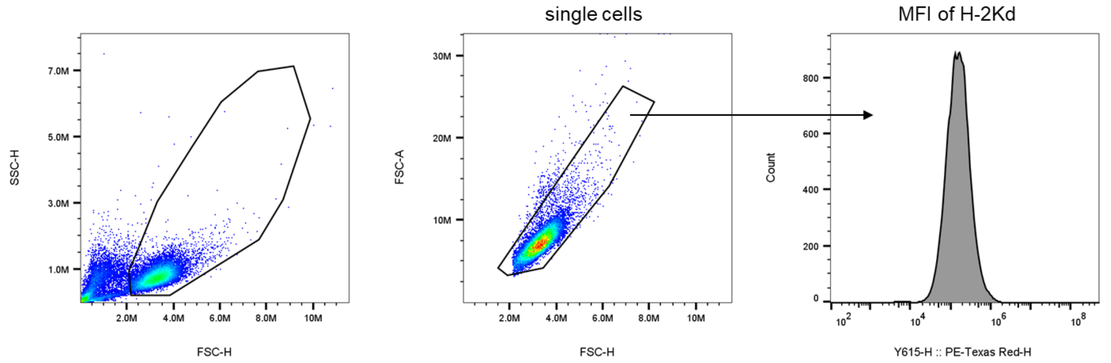

Figure S15. Flow cytometric gating strategy for analyzing MHC-I (H-2Kd) expression in Figure S13-S14.

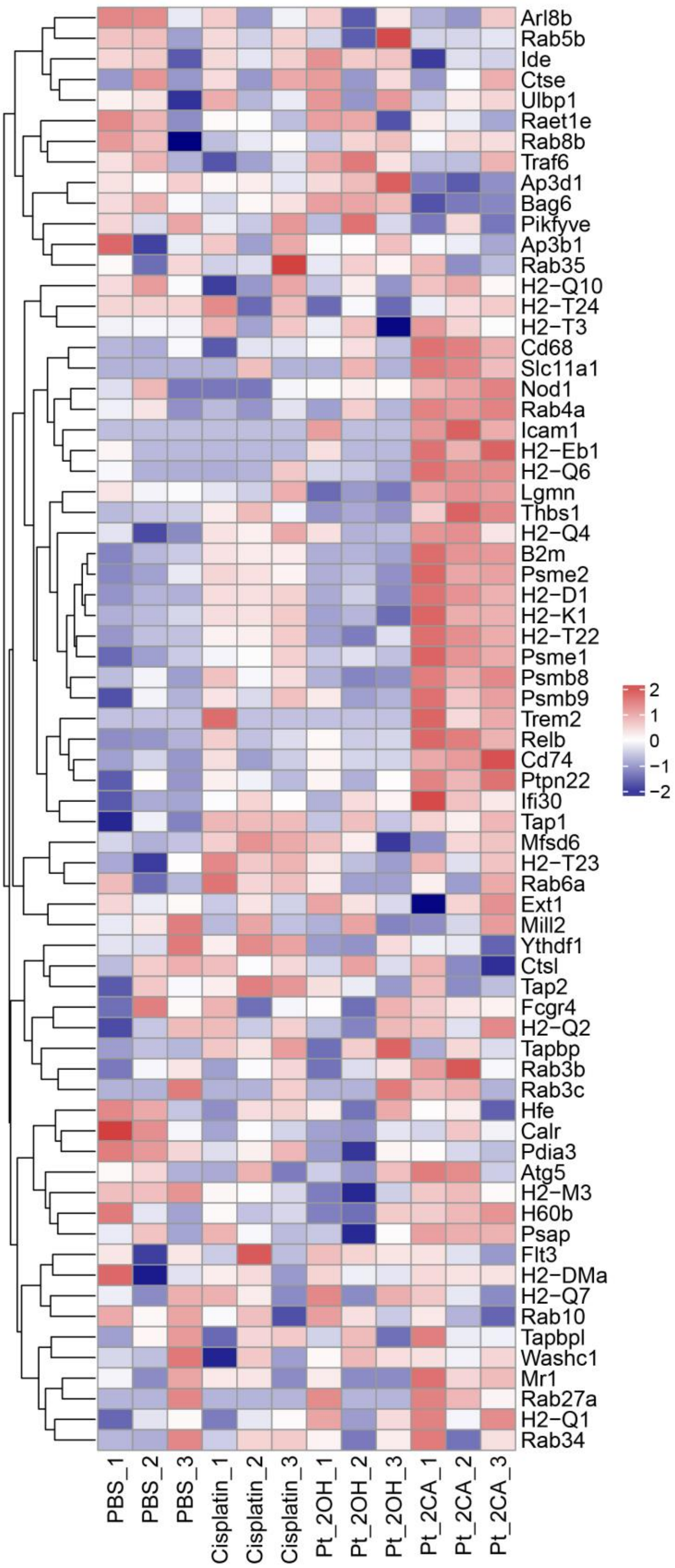

Figure S16. Heatmap showing expression of selected genes after cisplatin (0.2 μM), Pt-2OH (0.2 μM) and Pt-2CA (0.2 μM) treatment for 48 h.

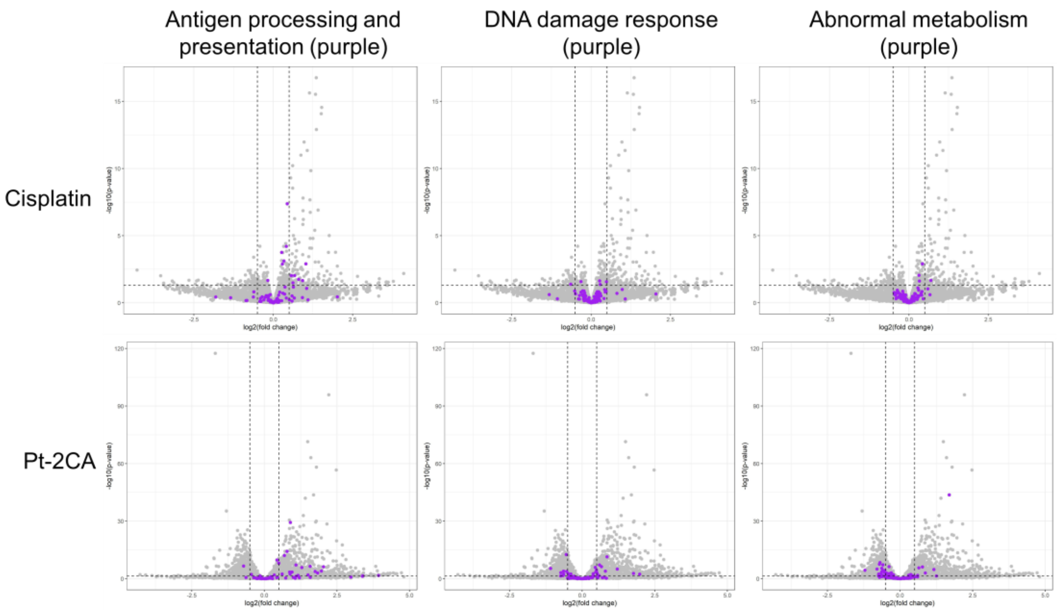

Figure S17. Volcano plot showing differential gene expression of various gene sets after 48 h treatment with cisplatin (0.2 μM) and Pt-2CA (0.2 μM). Compared to their effects on abnormal metabolism and DNA damage response, low-dose platinum drugs (especially Pt-2CA) had a more pronounced impact on antigen processing and presentation.

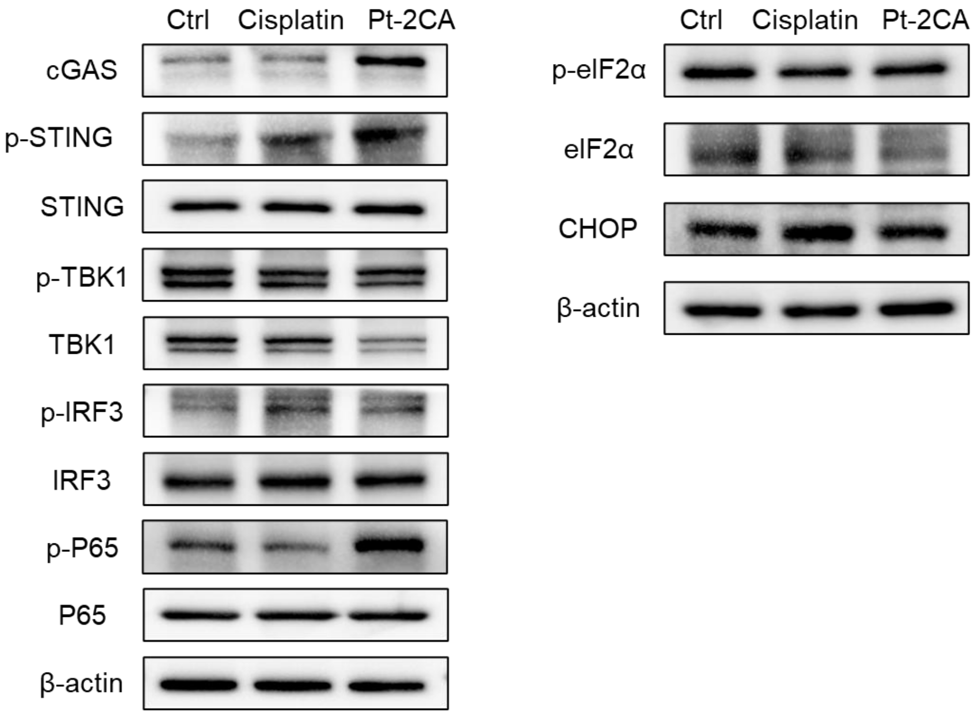

Figure S18. Western blot analysis of key components of the cGAS-STING pathway and the endoplasmic reticulum (ER) stress pathway in A549 cells treated with cisplatin (0.2 μM) or Pt-2CA (0.2 μM) for 48 h.

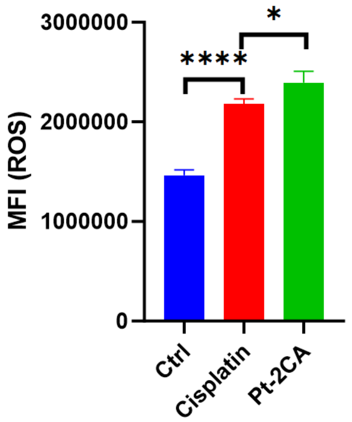

Figure S19. Intracellular reactive oxygen species (ROS) production in A549 cells treated with cisplatin (0.2 μM) or Pt-2CA (0.2 μM) for 48 h.

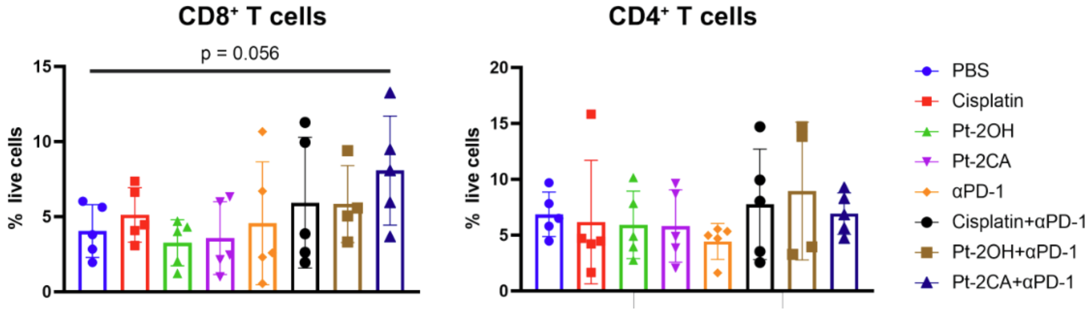

Figure S20. Flow cytometric analysis of $CD8^+$ T cells and $CD4^+$ T cells among live cells related to the tumor model of Figure 3B. Data are mean ± s.d. (n ⩾ 3).

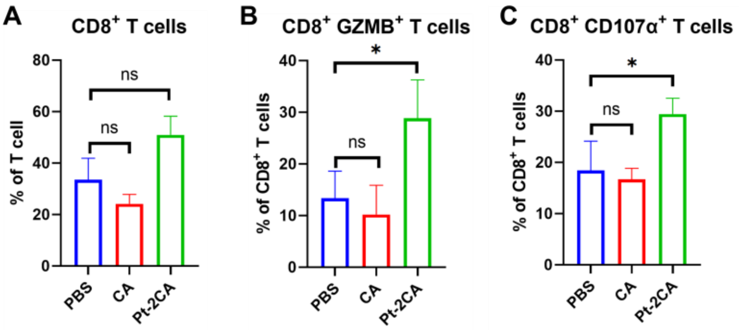

Figure S21. Flow cytometric analysis of the CT26-HER2 tumor model on day 6 following intravenous administration on day 0 and day 3 (0.5 mg CA/kg or 1 mg Pt-2CA/kg, with equivalent CA doses for CA and Pt-2CA treatments). (A) Percentage of $CD8^+$ T cells among T cells, (B-C) Percentage of $CD8^+GZMB^+$ T cells and $CD8^+CD107\alpha^+$ T cells within $CD8^+$ T cell population. CA: Cinnamic Acid. Data are mean ± s.d. (n ⩾ 3). *p < 0.05, **p < 0.01, ***p < 0.001.

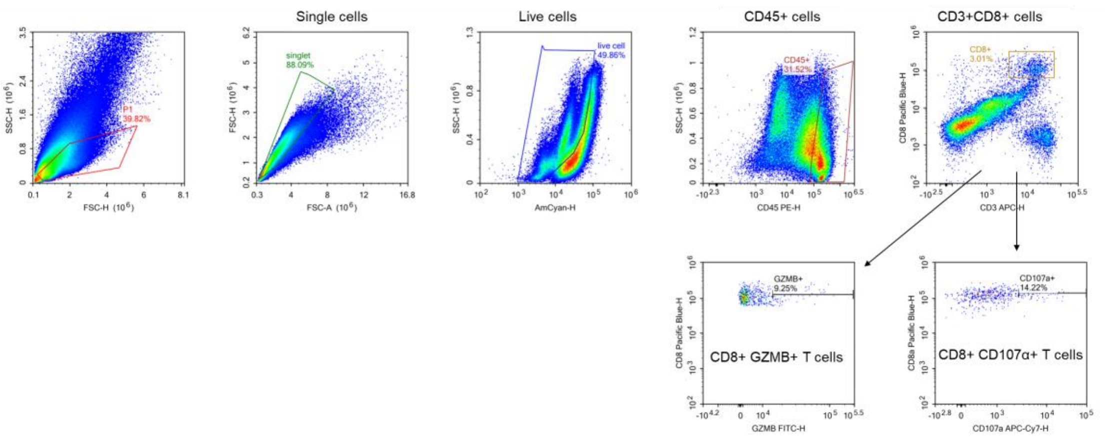

Figure S22. Flow cytometric gating strategy for the analysis of CD8⁺ T cells, CD8⁺GZMB⁺ T cells and CD8⁺CD107α⁺ T cells in Figure S21.

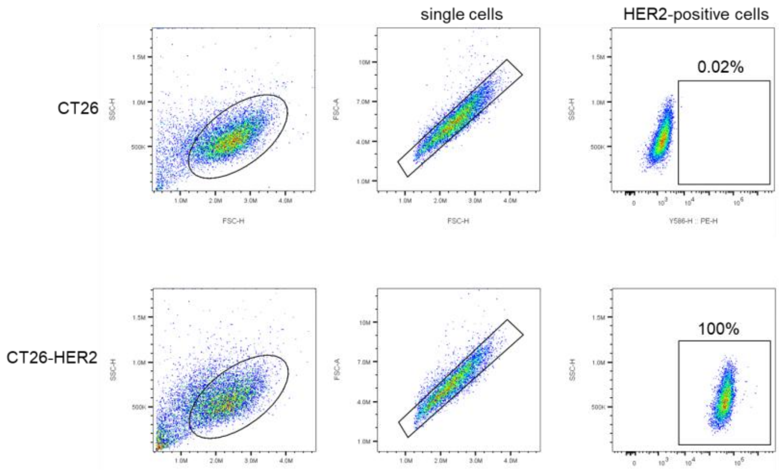

Figure S23. Flow cytometric analysis of HER2 overexpression in the CT26-HER2 cell line compared to the parental CT26 cell line, with staining using Herceptin-CY3.

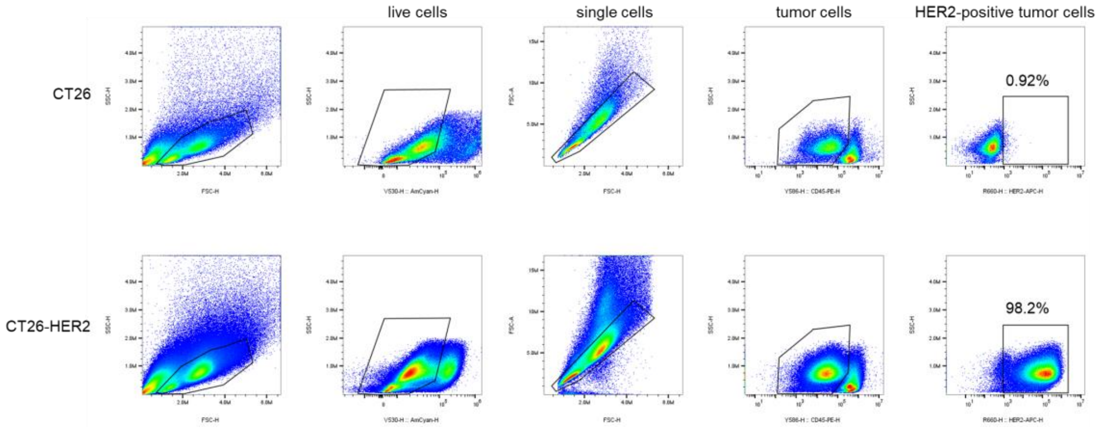

Figure S24. Flow cytometric analysis of HER2 overexpression in CT26-HER2 tumors compared to CT26 tumors.

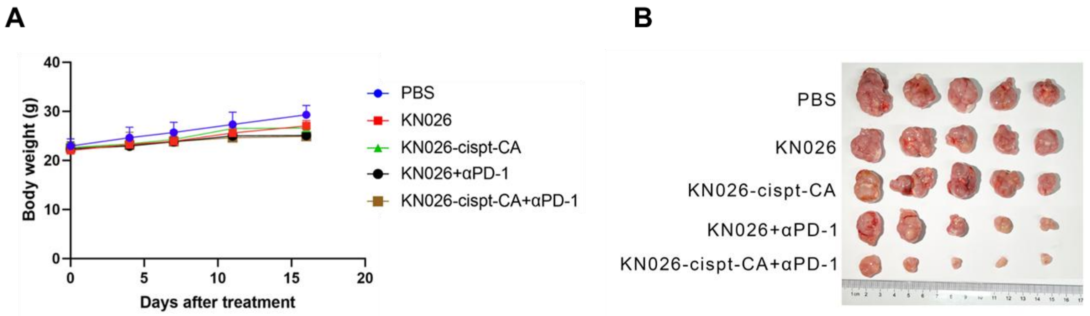

Figure S25. (A) Body weight changes during the course of treatment described in Figure 3F. (B) Representative image of tumors at the end point, related to the tumor model of Figure 3F.

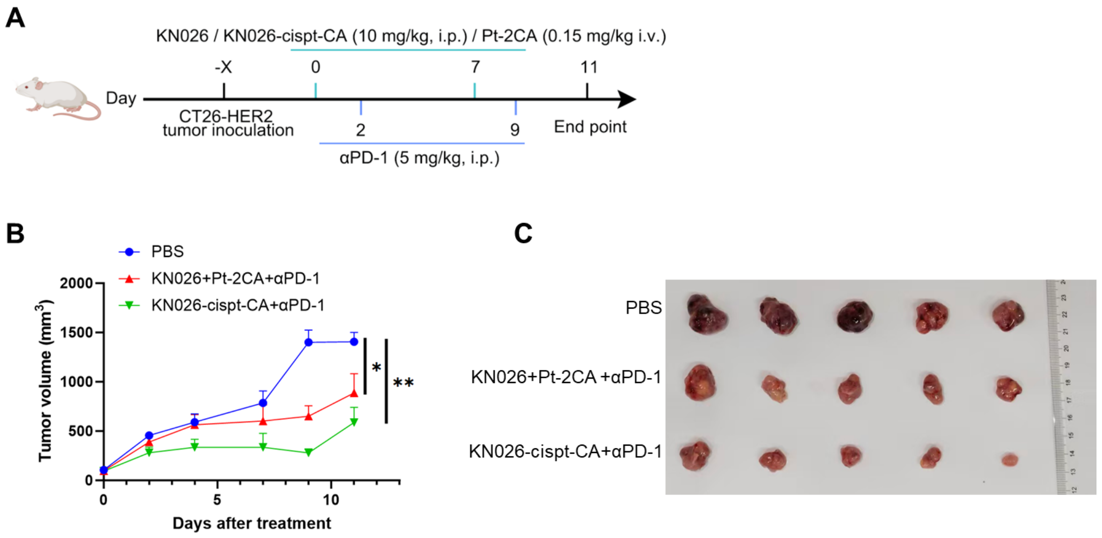

Figure S26. (A) Schematic illustration of the treatment regimen using Pt-ADC or an unconjugated antibody combined with free small-molecule platinum compound, each administered in combination with anti-PD-1 therapy, in the CT26-HER2 tumor model. (B) Tumor volume changes over the course of treatment. (C) Representative images of tumors at the experimental endpoint. The platinum content in the small-molecule platinum compound Pt-2CA (0.15 mg/kg) dose is equivalent to that in Pt-ADC (10 mg/kg) dose. Pt-ADC treatment resulted in approximately 58% tumor growth inhibition, whereas the corresponding unconjugated antibody plus free platinum compound achieved approximately 37% inhibition.

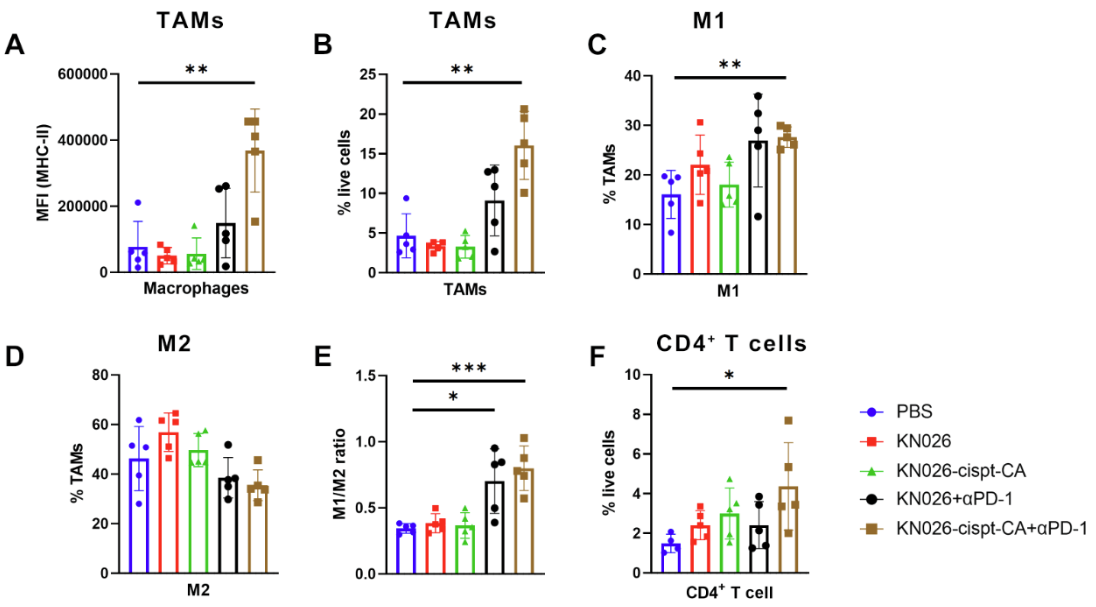

Figure S27. Flow cytometric analysis related to the tumor model of Figure 3F. (A) MHC-II (I-A/I-E) expression on tumor-associated macrophages (TAMs), (B) Percentage of TAMs among live cells, (C) M1 macrophage subsets within TAM population, (D) M2 macrophage subsets within TAM population, (E) The ratio of M1 to M2, (F) Percentage of $CD4^+$ T cells among live cells. Data are mean ± s.d. (n ⩾ 3). *p < 0.05, **p < 0.01, ***p < 0.001.

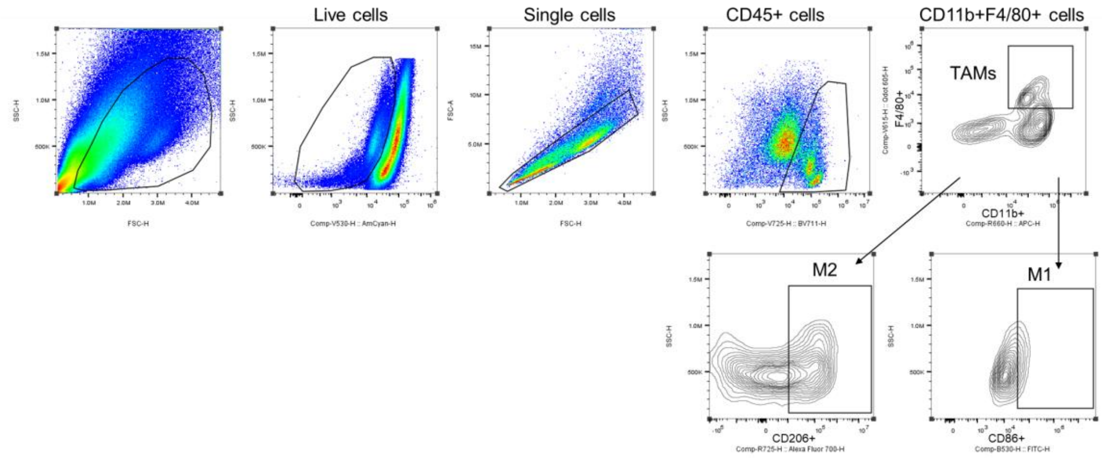

Figure S28. Flow cytometric gating strategy for the analysis of TAMs, M1 and M2 macrophage subsets in Figure S27.

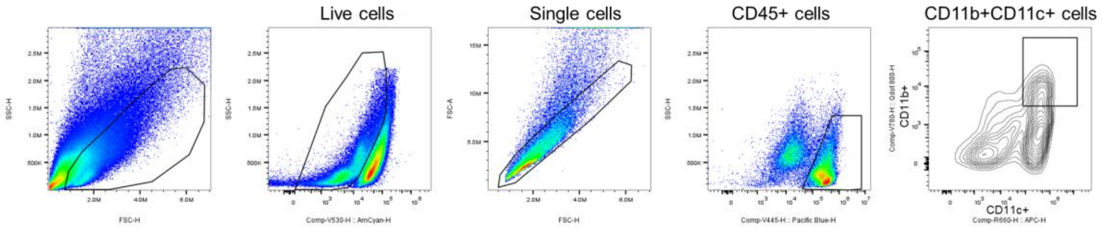

Figure S29. Flow cytometric gating strategy for the analysis of DCs in Figure 3.

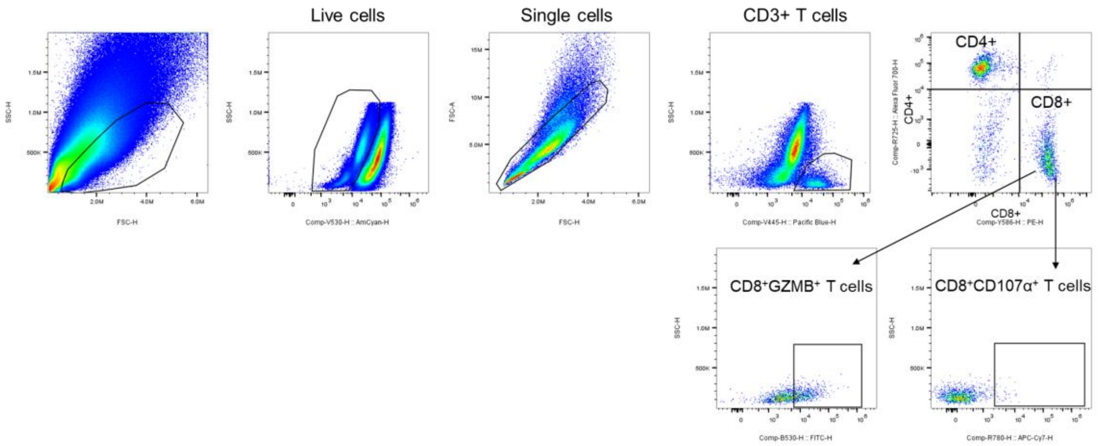

Figure S30. Flow cytometric gating strategy for the analysis of $CD3^+$ T cells, $CD4^+$ T cells, $CD8^+$ T cells, $CD8^+GZMB^+$ T cells and $CD8^+CD107\alpha^+$ T cells in Figure 3 and Figure S27.

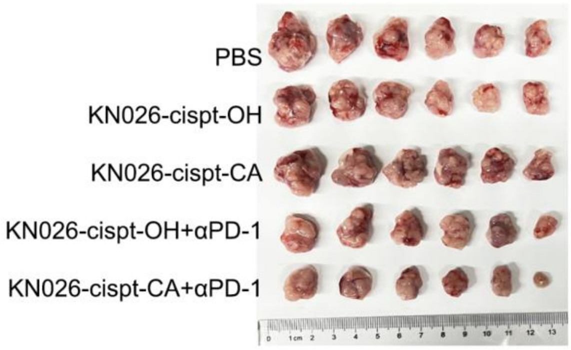

Figure S31. Representative image of tumors at the end point, related to the tumor model of Figure 3P.

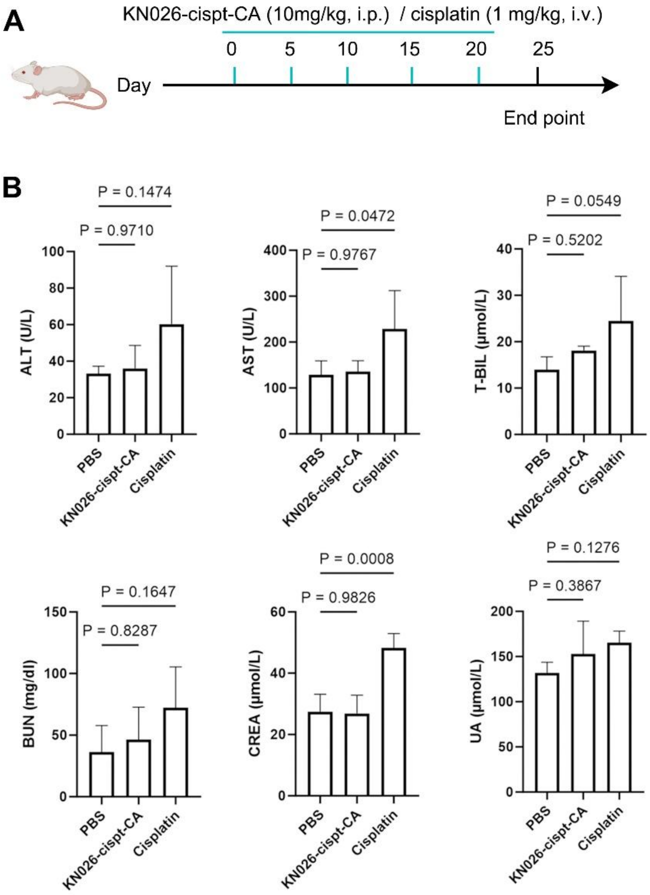

Figure S32. (A) Schematic representation of the treatment regimen in female BALB/c mice. (B) Evaluation of liver and kidney function through serum biochemical markers. Liver function markers: Alanine aminotransferase (ALT), Aspartate aminotransferase (AST), Total bilirubin (T-BIL); kidney function markers: Blood urea nitrogen (BUN), Creatinine (CREA), Uric acid (UA).

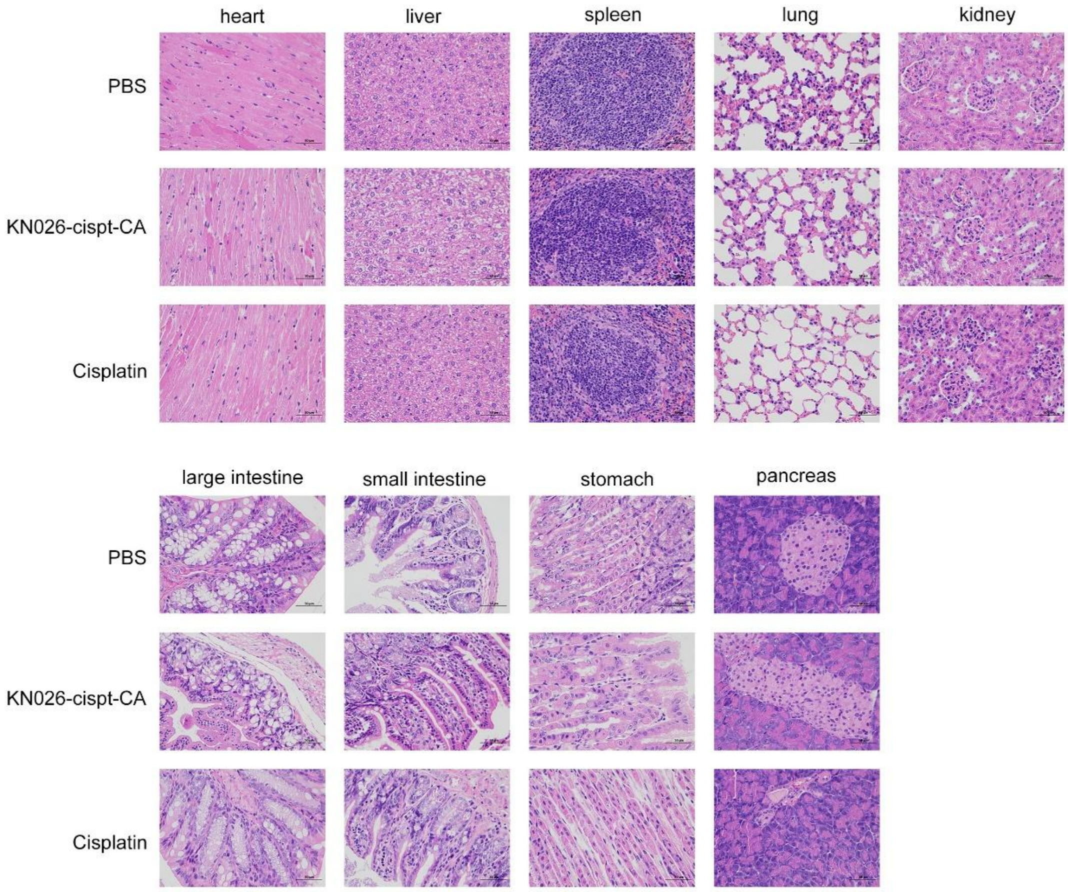

Figure S33. Histological examination of mouse organs after staining with hematoxylin-eosin (H&E) at the endpoint of the treatment in Figure S32A.

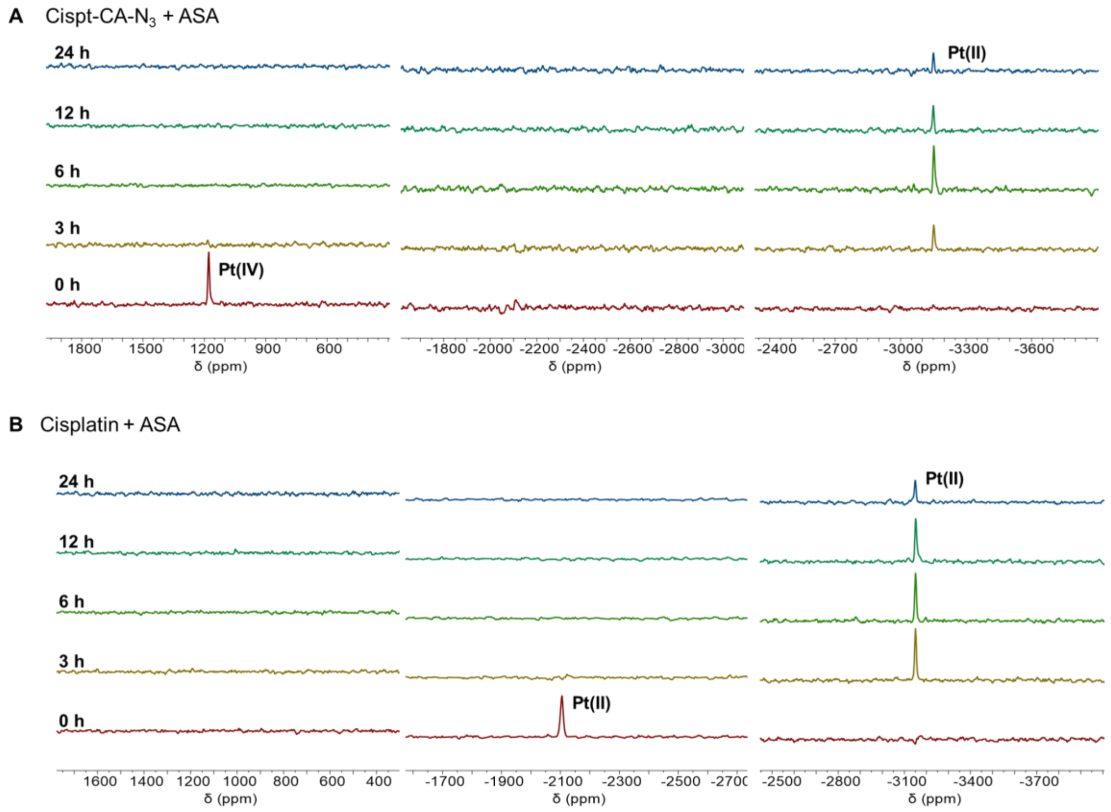

Figure S34. ^{195}Pt-NMR spectra for the reaction of (A) Cispt-CA-N3 or (B) cisplatin with ASA at 37 °C in the dark.

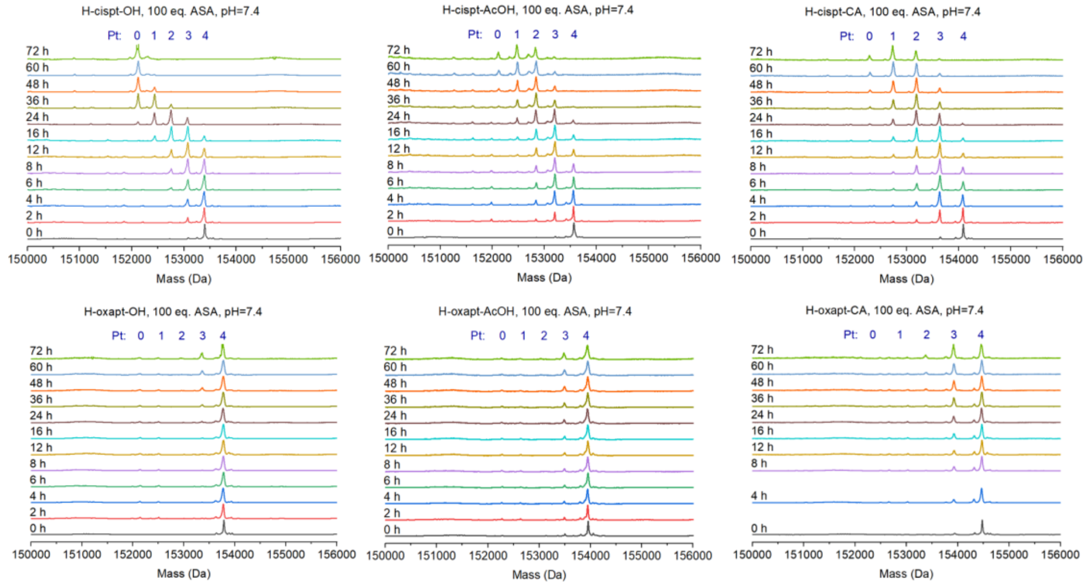

Figure S35. LC-MS analysis of the stepwise release of Pt-ADCs in the presence of 100 equivalent ASA (PBS, pH 7.4).

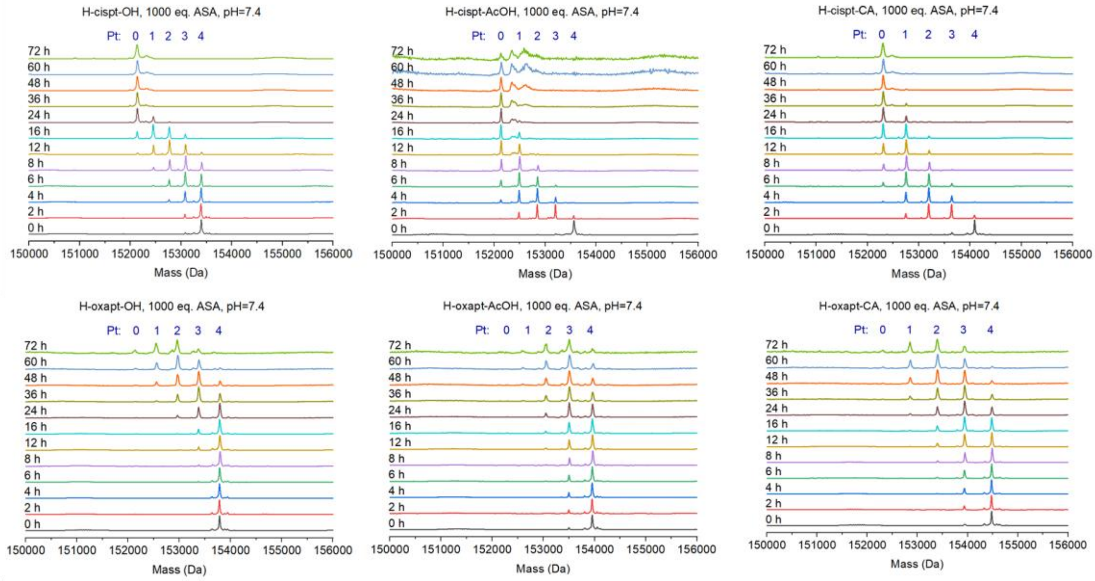

Figure S36. LC-MS analysis of the stepwise release of Pt-ADCs in the presence of 1000 equivalent ASA (PBS, pH 7.4).

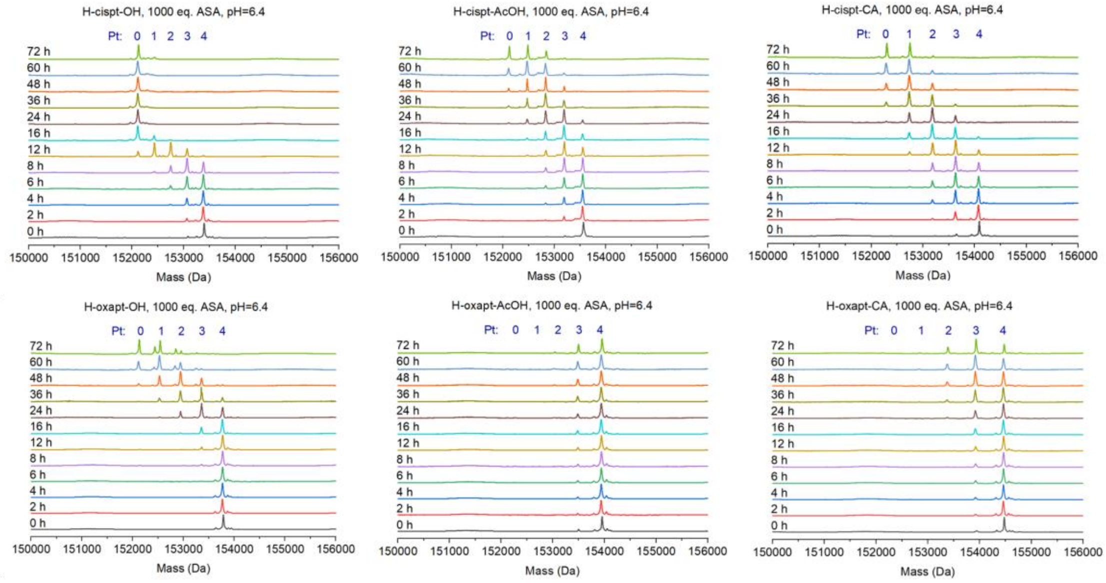

Figure S37. LC-MS analysis of the stepwise release of Pt-ADCs in the presence of 1000 equivalent ASA (PBS, pH 6.4).

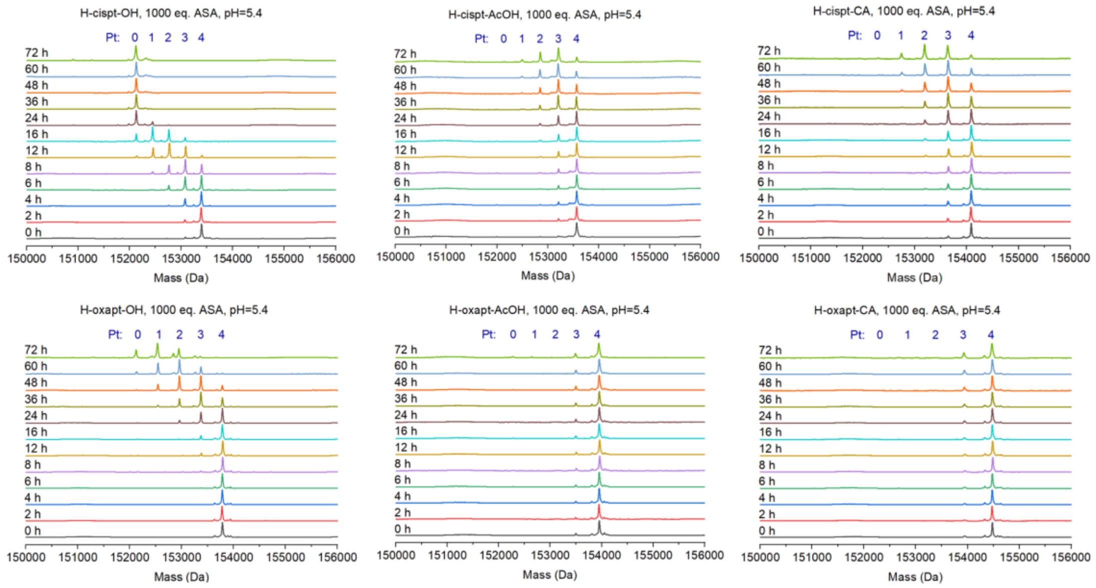

Figure S38. LC-MS analysis of the stepwise release of Pt-ADCs in the presence of 1000 equivalent ASA (PBS, pH 5.4).

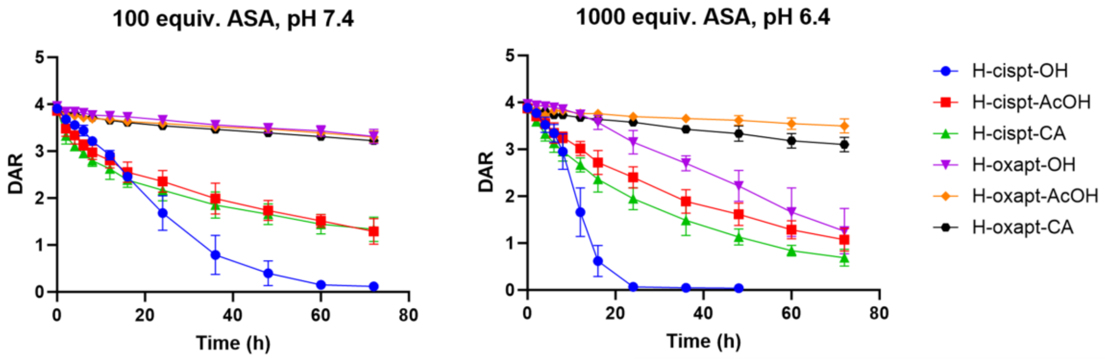

Figure S39. Reduction rate of Pt-ADCs under varying equivalents of ASA and different pH conditions.

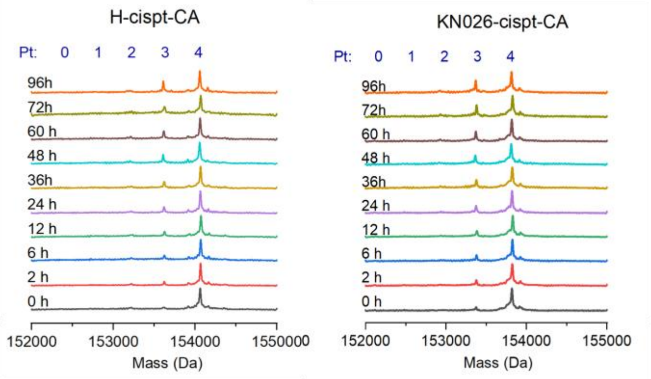

Figure S40. Comparison of the stability and reduction rate between H-cispt-CA and KN026-cispt-CA by LC-MS analysis in the presence of ASA (PBS, pH 7.4). The results indicate that H-cispt-CA and KN026-cispt-CA exhibit comparable stability and reduction rates, suggesting little dependence on the conjugated antibody.

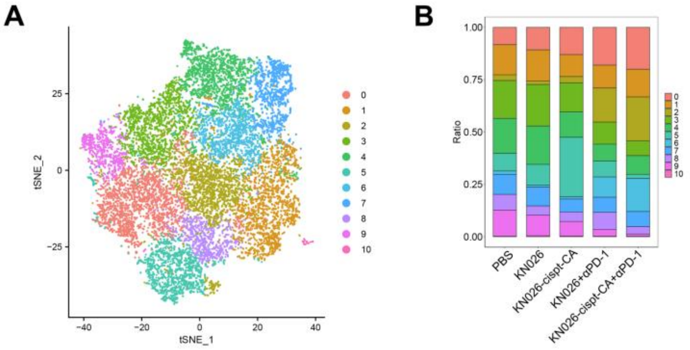

Figure S41. (A) t-SNE plots showing sorted tumor cells collected at the endpoint of the treatment in Figure 5A. (B) Proportions of different tumor cell subpopulations.

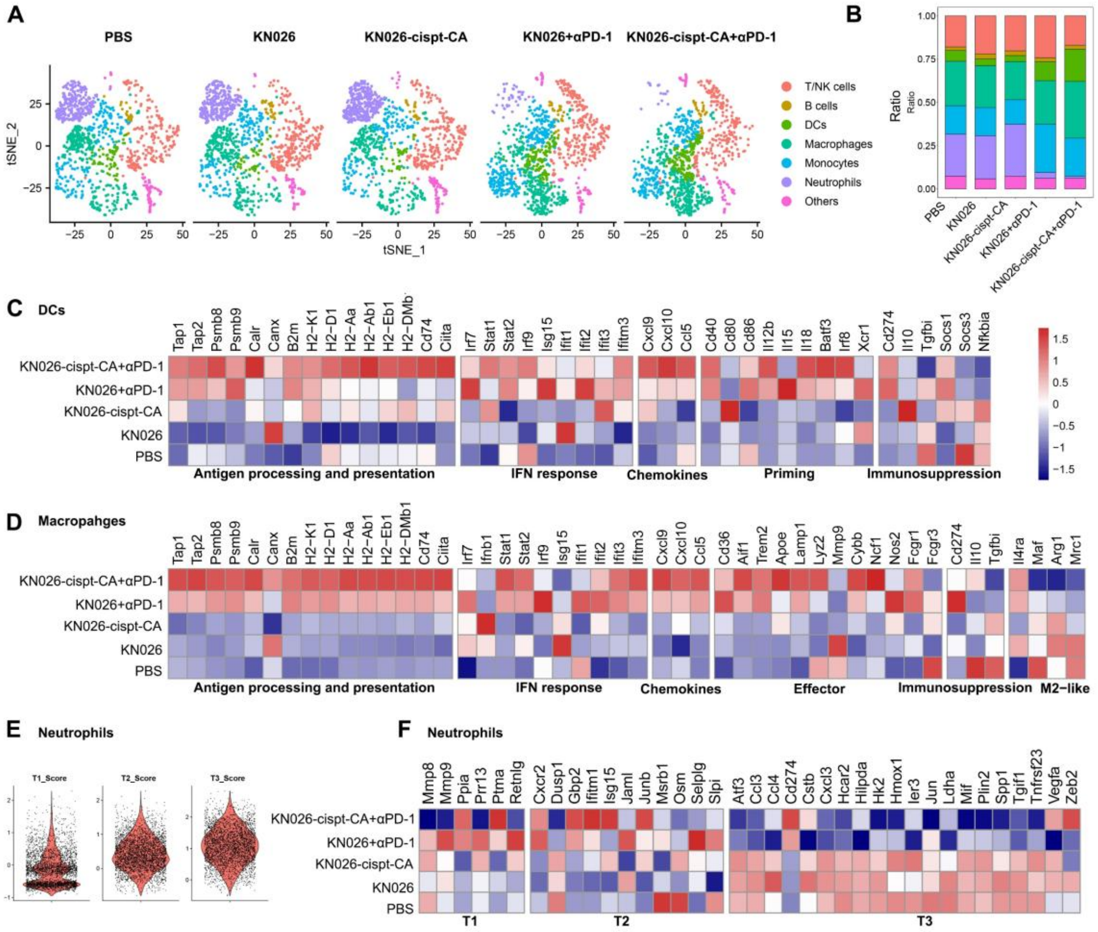

Figure S42. (A) t-SNE plots showing sorted CD45⁺ tumor-infiltrating immune cells collected at the endpoint of the treatment in Figure 5A. (B) Proportions of the major immune cell lineages within the CD45⁺ population. (C) Heatmap showing the expression of selected genes in dendritic cells (DCs) across treatment groups. (D) Heatmap showing the expression of selected genes in macrophages across treatment groups. (E) Violin plots displaying T1-, T2-, and T3-type scores in neutrophil subpopulations. (F) Heatmap showing the expression of selected genes in neutrophils across treatment groups.

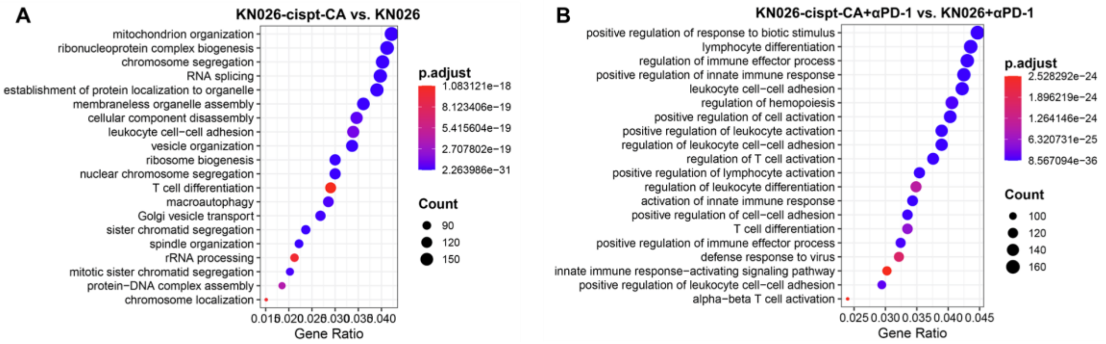

Figure S43. GO terms enriched in differentially expressed genes of CD8$^+$ T cell subsets (A) in the KN026-cispt-CA group compared to the KN026 group, and (B) in the KN026-cispt-CA+αPD-1 group compared to the KN026+αPD-1 group, under the conditions shown in Figure 5A.

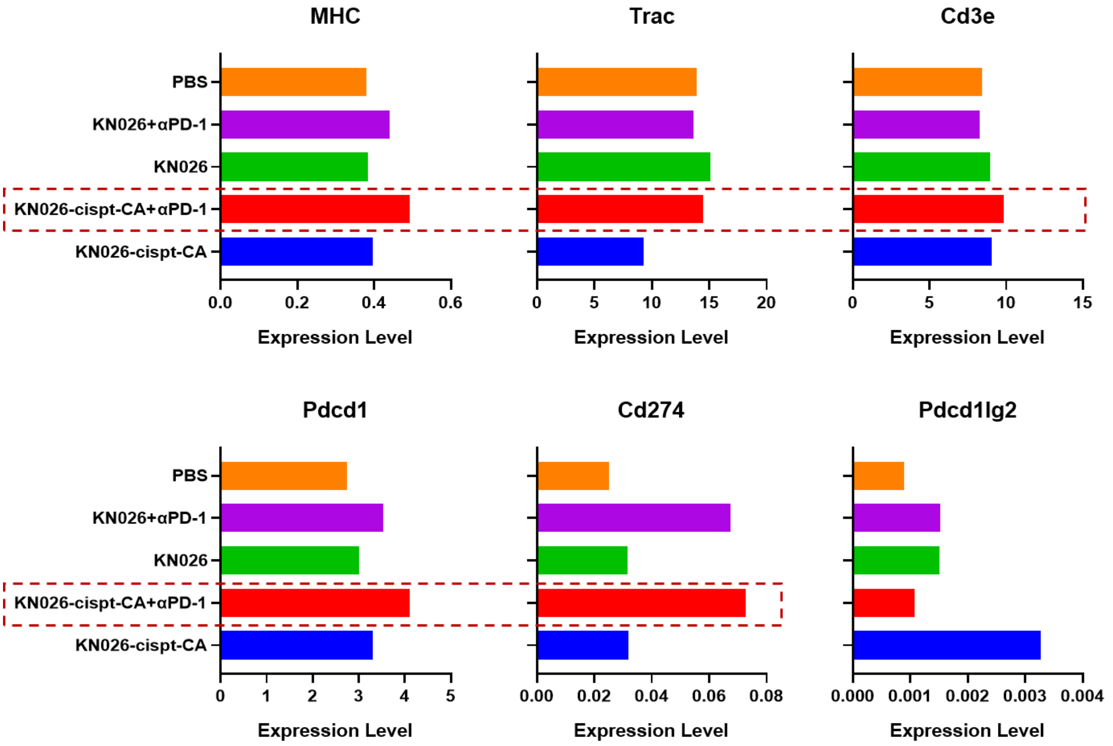

Figure S44. Analysis of the expression of MHC-TCR and PD1-PD-L1 ligand-receptor pairs in the scRNA-seq data from the treatment shown in Figure 5A, indicating that in the KN026-cispt-CA plus anti-PD-1 treatment group, both the MHC-TCR and PD1-PD-L1 ligand-receptor pairs showed enhanced expression.

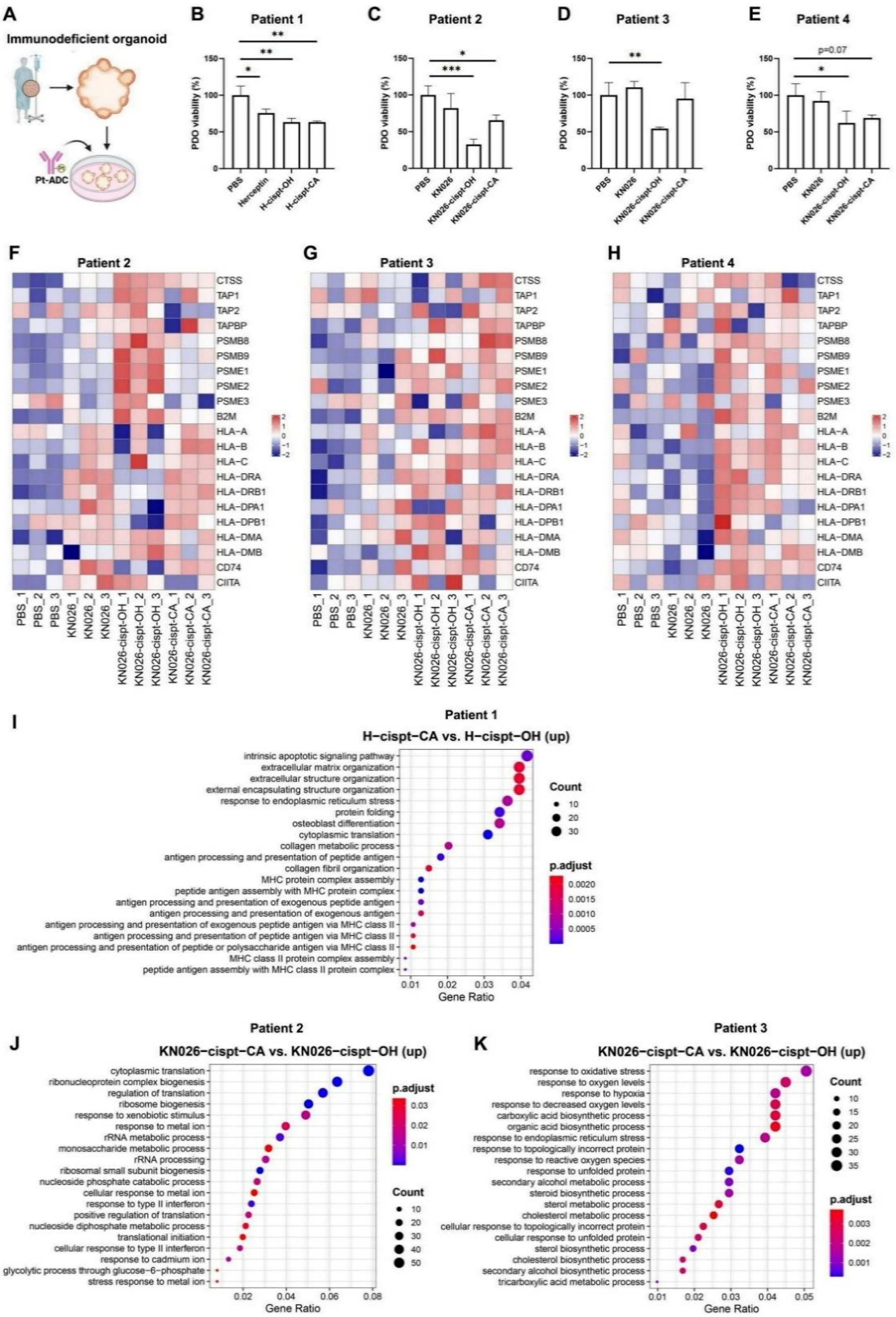

Figure S45. (A) Schematic of the HER2-positive immunodeficient patient-derived organoid (PDO) for Pt-ADC testing. (B-E) Tumor growth inhibition in immunodeficient PDOs following Pt-ADC (300 μg/mL) treatment for 84 h. (F-H) Heatmap showing expression of selected genes across treatment groups. (I-K) GO terms enriched in differentially expressed genes of PDO in the Cispt-CA based ADC group compared to the Cispt-OH based ADC group. Data are mean ± s.d. (n ⩾ 3). *p < 0.05, **p < 0.01, ***p < 0.001.

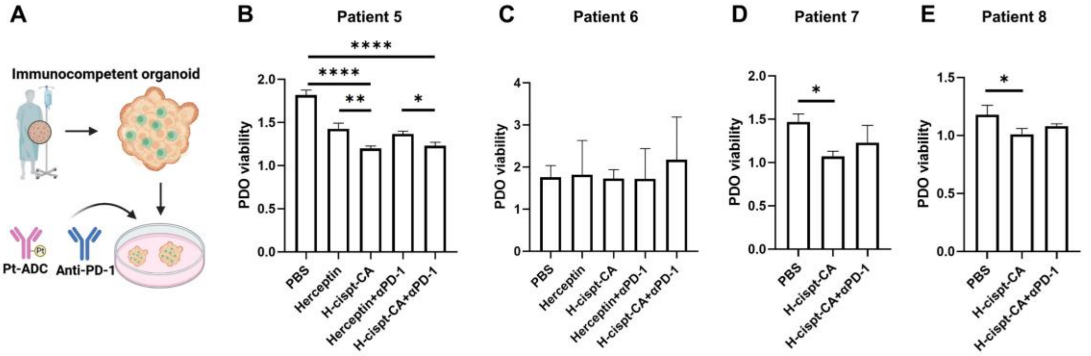

Figure S46. (A) Schematic of the HER2-positive immunocompetent PDO for Pt-ADC testing. Tumor specimens were collected and cultured to generate tumor-like cell clusters retaining autologous immune components. (B-E) PDO viability in immunocompetent PDOs following Pt-ADC (300 μg/mL) and anti-PD-1 (10 μg/mL) treatment for 7 days. Data are mean ± s.d. (n ⩾ 3). *p < 0.05, **p < 0.01, ***p < 0.001.

Supplementary Tables

Table S1. Platinum distribution in mouse tumors and various organs after administration of Pt-ADC or cisplatin (48 h) [a].

Organ	PBS	KN026-cispt-OH (10mg/kg)		Cisplatin (1.0 mg/kg)		$R_{C1/C2}$ [d]	$R_{B2/B1}$ [e]
	Pt content (ng/g) [b]	Pt content (ng/g) [b]	Pt bioavailability (%) [c]	Pt content (ng/g) [b]	Pt bioavailability (%) [c]		
tumor	0.18 ± 0.09	14.4 ± 6.1	0.43 ± 0.18	39.0 ± 15.8	0.09 ± 0.04	2.7	4.8
heart	0.38 ± 0.09	8.66 ± 3.14	0.08 ± 0.03	30.0 ±5.9	0.02 ± 0.01	3.5	3.6
liver	0.85 ± 0.64	22.5 ± 5.8	1.47 ± 0.35	154 ± 52	0.80 ± 0.27	6.9	1.8
spleen	1.58 ± 1.61	22.9 ± 7.5	0.20 ± 0.06	59.7 ± 27.4	0.04 ± 0.02	2.6	4.8
lung	1.10 ± 0.46	9.70 ± 1.53	0.15 ± 0.02	115 ± 19	0.16 ± 0.03	12	1.0
kidney	0.26 ± 0.03	15.2 ± 5.8	0.39 ± 0.15	215 ± 45	0.43 ± 0.09	14	0.9
brain	0.33 ± 0.11	0.78 ± 0.37	0.02 ± 0.01	7.40 ± 5.94	0.02 ± 0.02	9.5	0.8
large intestine	0.27 ± 0.08	10.4 ± 5.1	0.24 ± 0.12	41.5 ± 17.1	0.08 ± 0.03	4.0	3.2
small intestine	0.18 ± 0.02	4.46 ± 0.56	0.43 ± 0.05	33.4 ± 6.9	0.26 ± 0.05	7.5	1.7
stomach	0.52 ± 0.35	6.82 ± 1.25	0.09 ± 0.01	30.1 ± 8.9	0.03 ± 0.01	4.4	2.8
pancreas	0.43 ± 0.09	9.98 ± 3.88	0.76 ± 0.30	45.8 ± 18.2	0.28 ± 0.11	4.6	2.7

[a] In CT26-HER2 tumor-bearing mice, 48 h after intraperitoneal injection of KN026-cispt-OH (10mg/kg) or intravenous injection of cisplatin (1.0 mg/kg), followed by measurement using ICP-MS.

[b] Pt content (ng) in tumor/organ (g). The PBS group represents the background value of the assay.

[c] Pt content in tumor/organ as a percentage of the total administered dose, indicating the bioavailability of Pt in the tumor/organ.

[d] Ratio of Pt content in the cisplatin treatment group to the KN026-cispt-OH treatment group, reflecting how many times higher the platinum content in tumor/organ is in the cisplatin treatment compared to the KN026-cispt-OH treatment.

[e] Ratio of Pt bioavailability in the KN026-cispt-OH treatment group to the cisplatin treatment group, indicating how many times greater the platinum bioavailability is in the KN026-cispt-OH treatment compared to the cisplatin treatment.

Chemical Synthesis and Characterization

Chemical Synthesis

Materials and measurements. All materials were purchased from commercial suppliers and used as received unless otherwise specified. NMR spectra were acquired on a Bruker Advance 500 MHz or 600 MHz spectrometer (Germany), with shifts referenced to the internal solvent signals. Mass spectrometric analyses were performed on a Waters SQD2 single quadrupole mass spectrometer equipped with an electrospray ionization (ESI) source. Oxoplatin (Pt-2OH) was synthesized by oxidizing cisplatin with 30% H_2O_2, following a previously reported method[4]. Pt-2CA was synthesized by coupling with cinnamic acid, following a previously reported method[4-5].

Synthesis of Cispt-OH-N_3. To a suspension of oxoplatin (0.3 mmol) in DMSO (5 mL), Ligand 1 (0.35 mmol) was added. The reaction mixture was stirred at 60 °C for 12 h. Excess diethyl ether (100 mL) was added to remove DMSO and precipitate the product. The resulting crude solid was purified by HPLC to yield a light yellow solid (Yield: 38%). ^{1}H NMR (600 MHz, DMSO-d_6) δ 5.95 (td, J = 52.5, 52.1, 25.9 Hz, 6H), 3.32 – 3.30 (m, 2H), 2.17 (t, J = 7.4 Hz, 2H), 1.51 (dp, J = 27.0, 7.3 Hz, 4H), 1.33 (qd, J = 8.8, 6.4 Hz, 2H). ^{13}C NMR (151 MHz, DMSO) δ 183.54, 51.08, 34.85, 29.29, 26.33, 25.60. ESI-MS (ACN) m/z: calculated for [Cispt-OH-N_3 - H]$^-$ 471.03; Found 471.81.

Synthesis of Cispt-AcOH-N_3. Cispt-OH-N_3 (0.3 mmol) and acetic anhydride (0.6 mmol) were added to DMF (5 mL) and stirred at 60 °C for 12 h. After completion, DMF was removed under reduced pressure. Acetone (5 mL) and diethyl ether (40 mL) were then added to precipitate the product, affording a light yellow solid (Yield: 72%). ^{1}H NMR (600 MHz, DMSO-d_6) δ 6.67 – 6.37 (m, 6H), 3.31 (t, J = 6.9 Hz, 3H), 2.23 (t, J = 7.4 Hz, 2H), 1.91 (s, 3H), 1.55 – 1.46 (m, 4H), 1.36 – 1.31 (m, 2H). ^{13}C NMR (151 MHz, DMSO) δ 182.96, 177.30, 52.71, 37.35, 29.29, 26.17, 25.41, 23.34. ESI-MS (ACN) m/z: calculated for [Cispt-AcOH-N_3 - H]$^-$ 513.04; Found 513.94.

Synthesis of Cispt-CA-N_3. To a suspension of oxoplatin (0.3 mmol) in DMSO (5 mL), Ligand 2 (0.35 mmol) was added, and the mixture was stirred at 60 °C for 12 h. Excess diethyl ether (100 mL) was added to remove DMSO. The resulting solid was extracted with diethyl ether and dried under vacuum to obtain a light yellow solid (Complex 1; Yield: 42%). Complex 1 (0.3 mmol) and succinic anhydride (0.6 mmol) were dissolved in DMF (5 mL) and stirred at 60 °C for 12 h. After solvent removal under reduced pressure, acetone (5 mL) and diethyl ether (40 mL) were added to precipitate the product. A light yellow solid was obtained (Complex 2; Yield: 75%). Complex 2 (0.2 mmol), NHS (0.6 mmol), and EDC (0.6 mmol) were dissolved in DMF (10 mL) and stirred in an ice bath for 0.5 h. And then 3-azidopropan-1-amine (0.3 mmol) were added and stirred at room temperature for 8 h. After reaction completion, the solvent was removed under reduced pressure, and acetone (5 mL) and diethyl ether (40 mL) were added to precipitate the crude product. The final compound was purified by HPLC to yield a light yellow solid (Yield: 35%). ^{1}H NMR (600 MHz, DMSO-d_6) δ 7.89 (t, J = 5.7 Hz, 1H), 7.69 – 7.58 (m, 2H), 7.42 – 7.38 (m, 3H), 6.55 (d, J = 15.9 Hz, 7H), 3.36 (t, J = 6.8 Hz, 2H), 3.11 (d, J = 5.7 Hz, 2H),

2.47 (d, J = 7.5 Hz, 2H), 2.30 (t, J = 7.4 Hz, 2H), 1.66 (t, J = 6.8 Hz, 2H). ^{13}C NMR (151 MHz, DMSO) δ 179.93, 175.25, 169.90, 140.71, 135.69, 130.73, 129.39, 127.59, 123.30, 47.72, 36.35, 31.91, 31.84, 28.87. ESI-MS (ACN) m/z: calculated for [Cispt-CA-N_3 - H]$^-$ 645.07; Found 645.06.

Synthesis of Oxapt-OH-N_3. The synthesis followed the same procedure as for Cispt-OH-N_3, except that oxaliplatin-based complex was used in place of cisplatin-based complex. A white solid was obtained (Yield: 40%). ^1H NMR (500 MHz, DMSO-d_6) δ 7.88 (d, J = 750.3 Hz, 4H), 3.29 (t, J = 6.9 Hz, 2H), 2.47 (d, J = 11.7 Hz, 2H), 2.19 (h, J = 7.8 Hz, 2H), 2.12 – 2.03 (m, 2H), 1.52 – 1.23 (m, 10H), 1.11 (q, J = 11.4 Hz, 2H). ^{13}C NMR (126 MHz, DMSO) δ 182.62, 163.73, 62.03, 60.47, 51.92, 37.06, 31.32, 31.22, 28.51, 26.71, 25.57, 24.09. ESI-MS (ACN) m/z: calculated for [Oxapt-OH-N_3 - H]$^-$ 569.13; Found 569.00.

Synthesis of Oxapt-AcOH-N_3. The synthesis followed the same procedure as for Cispt-AcOH-N_3, except that oxaliplatin-based complex was used in place of cisplatin-based complex. A white solid was obtained (Yield: 68%). ^1H NMR (600 MHz, DMSO-d_6) δ 8.34 (d, J = 52.5 Hz, 4H), 3.29 (t, J = 6.9 Hz, 2H), 2.55 (s, 2H), 2.29 – 2.23 (m, 2H), 2.15 – 2.08 (m, 2H), 1.96 (s, 3H), 1.54 – 1.44 (m, 8H), 1.27 (t, J = 7.8 Hz, 2H), 1.17 – 1.10 (m, 2H). ^{13}C NMR (151 MHz, DMSO) δ 181.51, 179.01, 163.80, 61.71, 61.39, 50.96, 36.11, 27.94, 26.07, 25.35, 23.97, 23.44. ESI-MS (ACN) m/z: calculated for [Oxapt-AcOH-N_3 - H]$^-$ 611.14; Found 611.12.

Synthesis of Oxapt-CA-N_3. The synthesis followed the same procedure as for Cispt-CA-N_3, except that oxaliplatin-based complex was used in place of cisplatin-based complex. A white solid was obtained (Yield: 40%). ^1H NMR (600 MHz, DMSO-d_6) δ 8.30 (d, J = 42.8 Hz, 4H), 7.92 (t, J = 5.7 Hz, 1H), 7.64 (dd, J = 7.5, 2.2 Hz, 2H), 7.45 (d, J = 15.9 Hz, 1H), 7.40 (p, J = 2.2 Hz, 2H), 6.55 (d, J = 15.9 Hz, 1H), 3.35 (d, J = 6.8 Hz, 2H), 3.10 (d, J = 6.7 Hz, 2H), 2.54 (q, J = 7.9 Hz, 2H), 2.32 – 2.27 (m, 2H), 2.16 – 2.11 (m, 2H), 1.65 (d, J = 6.8 Hz, 2H), 1.55 – 1.41 (m, 6H), 1.22 – 1.16 (m, 2H). ^{13}C NMR (151 MHz, DMSO) δ 181.17, 174.29, 171.72, 163.94, 142.68, 134.71, 130.43, 129.38, 128.49, 122.28, 61.48, 61.27, 48.90, 36.34, 31.38, 28.89, 24.07, 23.87, 23.43. ESI-MS (ACN) m/z: calculated for [Oxapt-CA-N_3 - H]$^-$ 742.18; Found 742.31.

Synthesis of Antibody-G2F-(Fuc-DBCO)$_4$. GDP-Fuc-DBCO was synthesized as previously described[6]. Trastuzumab or KN026 (5 mg/mL) was incubated with UDP-Galactose (2 mM), bovine β-1,4-GalT1(Y289L) (0.3 mg/mL), GDP-Fuc-DBCO (2 mM) and α 1,3 FucT-2 HR (0.5 mg/mL) in 25 mM Tris-HCl buffer (pH 7.5) with 10 mM $MgCl_2$ and 5 mM $MnCl_2$ at 30 °C for 16 h. The modified antibody was purified with protein A resin and confirmed by UPLC-TOF/MS analysis.

Synthesis of Antibody-G2F-(Fuc-DBCO-N_3-Pt)$_4$ (Pt-ADC). Antibody-G2F-(Fuc-DBCO)$_4$ (2 mg/mL) was incubated with azide-functionalized platinum complexes (Pt-N_3, 268 uM; including Cispt-OH-N_3, Cispt-AcOH-N_3, Cispt-CA-N_3, Oxapt-OH-N_3, Oxapt-AcOH-N_3, or Oxapt-CA-N_3) in PBS at 30 °C for 1 h. Due to the excess Pt-N_3, more than 99% of the DBCO groups on the antibody underwent conjugation. The resulting Pt-ADCs were purified using 100 kDa molecular

weight cutoff ultrafiltration and characterized by UPLC-TOF/MS analysis. The purified yield was greater than 80%. Hydrophobic interaction chromatography (HIC) and size-exclusion chromatography (SEC) analyses were performed by Glyco-therapy Biotechnology Co., Ltd.

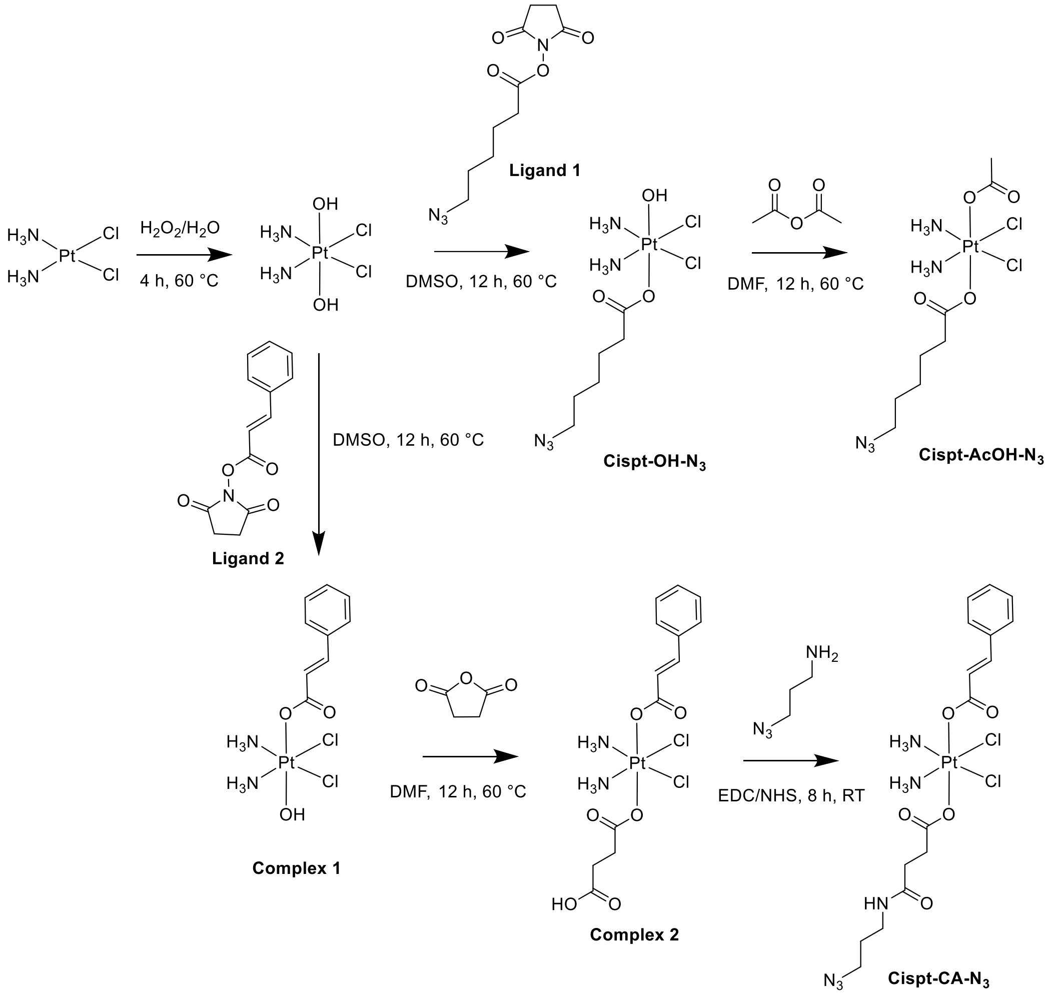

Scheme S1. The synthetic routes of Cispt-OH-N_3, Cispt-AcOH-N_3 and Cispt-CA-N_3.

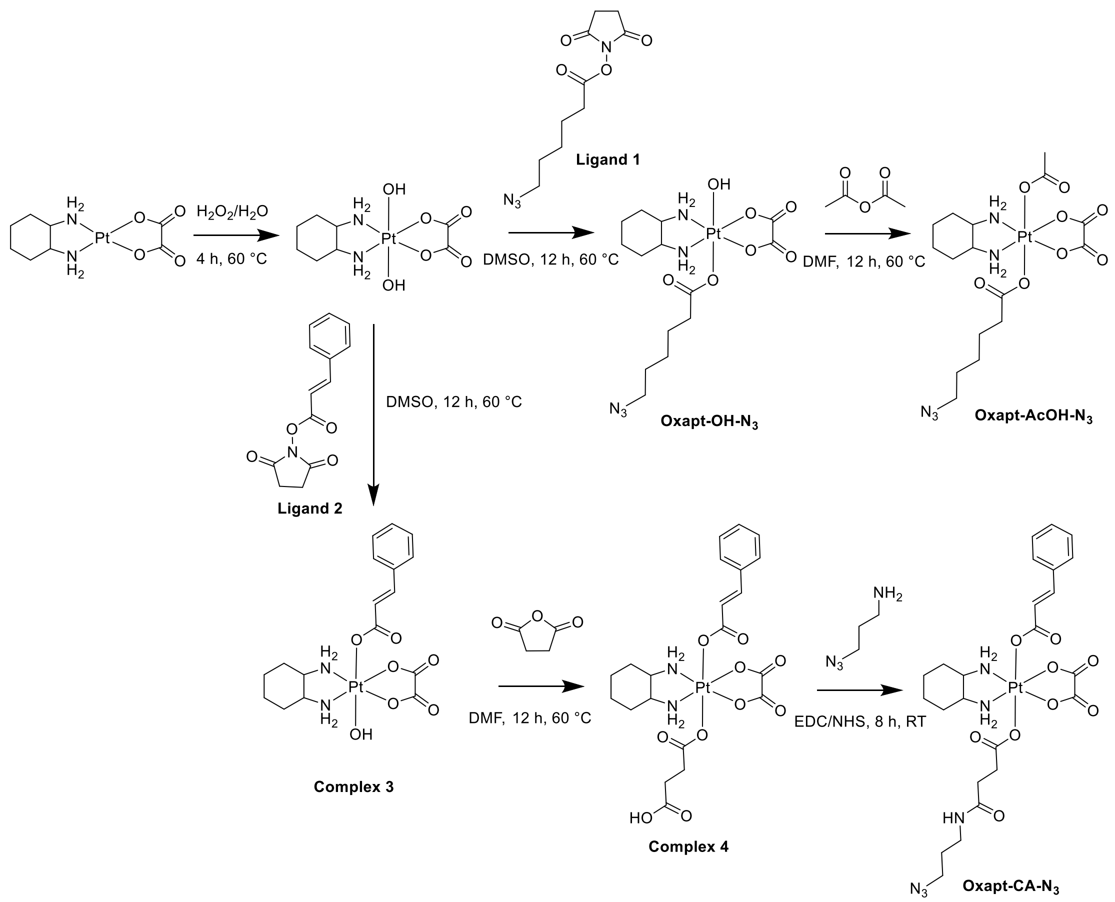

Scheme S2. The synthetic routes of Oxapt-OH-N_3, Oxapt-AcOH-N_3 and Oxapt-CA-N_3.

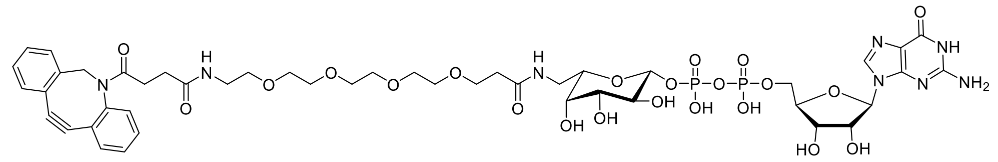

Scheme S3. The structure of GDP-Fuc-DBCO.

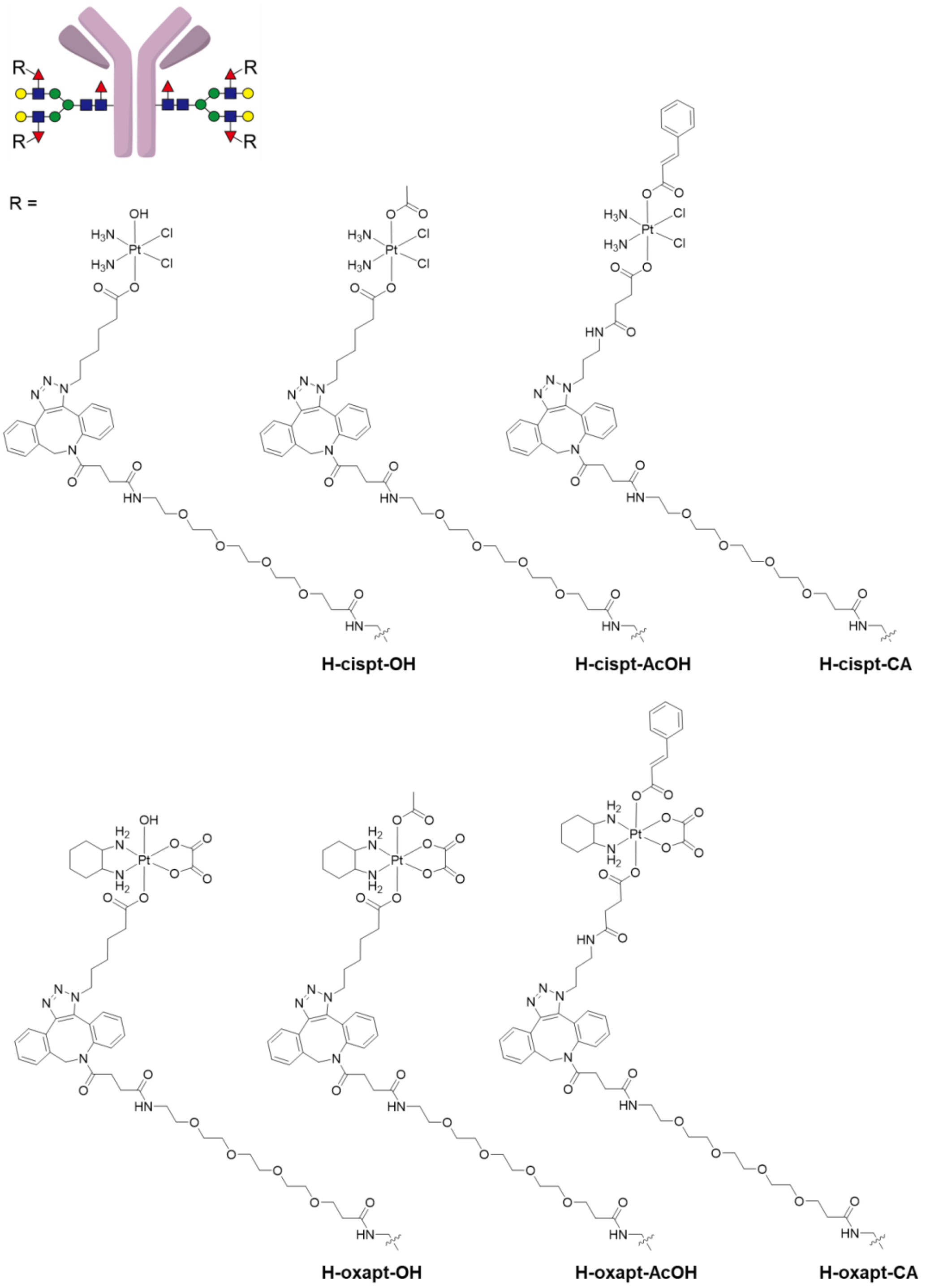

Scheme S4. The structure of Pt-ADCs.

NMR and MS spectra

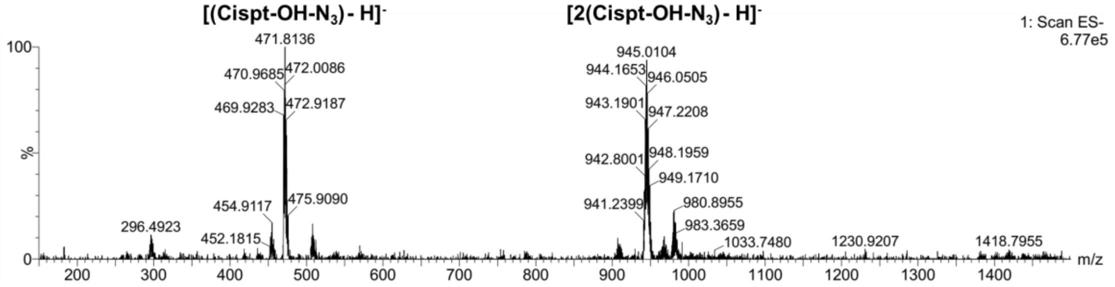

Figure S47. Mass spectrum of Cispt-OH-N_3.

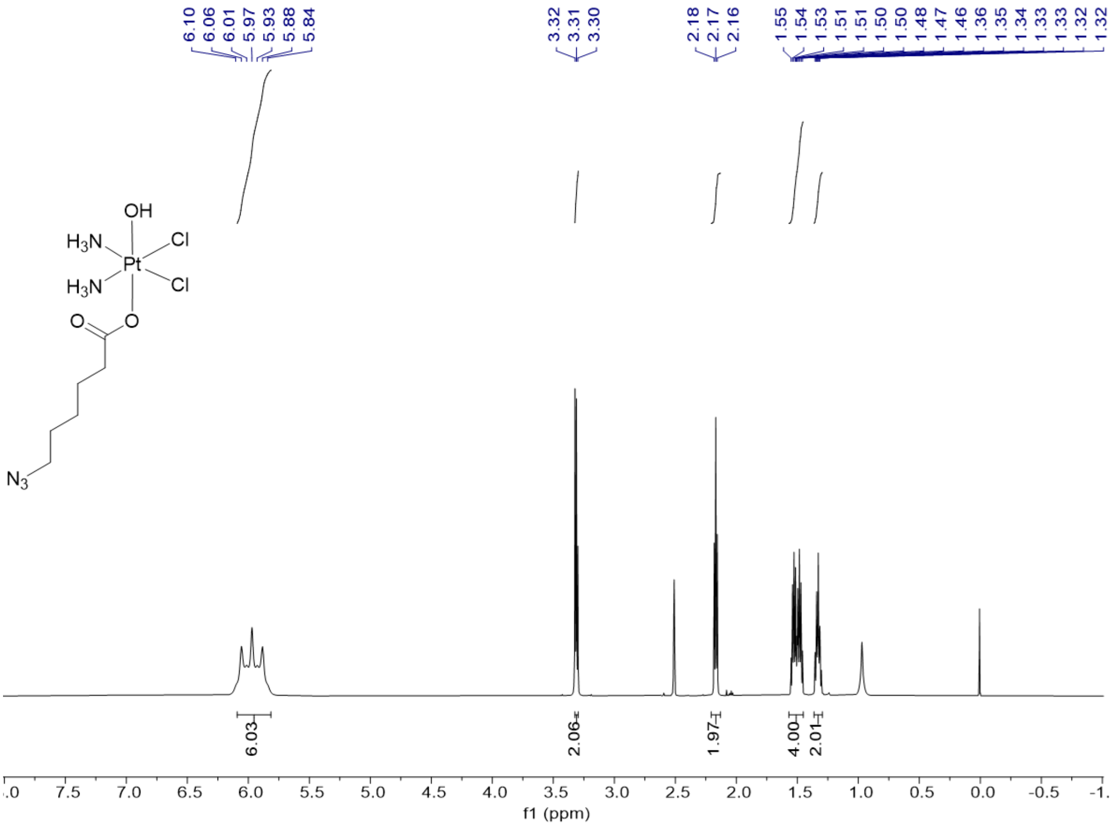

Figure S48. ^{1}H NMR spectrum of Cispt-OH-N_3 in DMSO-d_6.

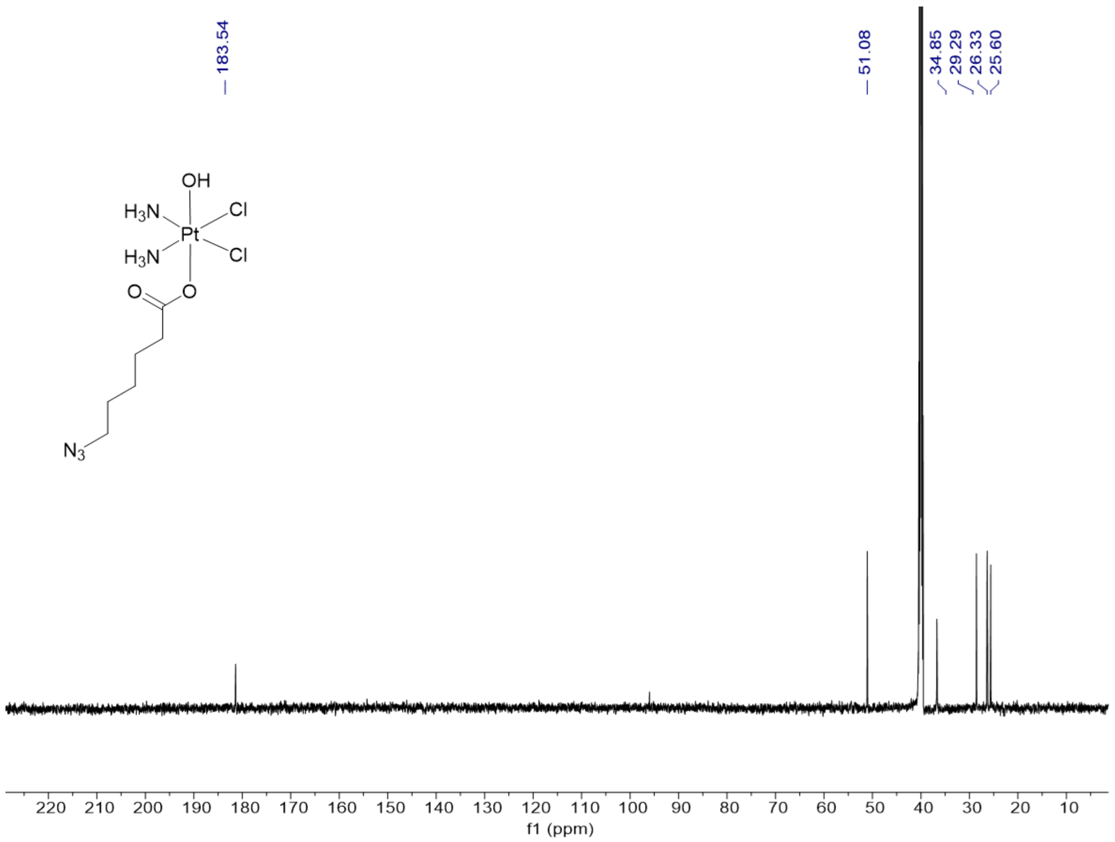

Figure S49. ^{13}C NMR spectrum of Cispt-OH-N_3 in DMSO-d_6.

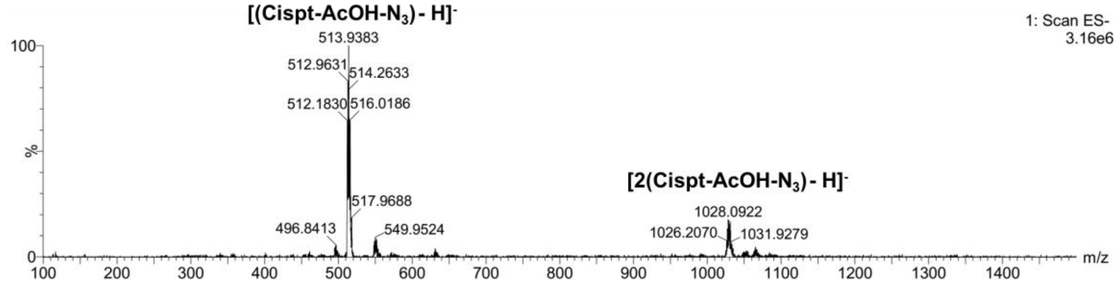

Figure S50. Mass spectrum of Cispt-AcOH-N_3.

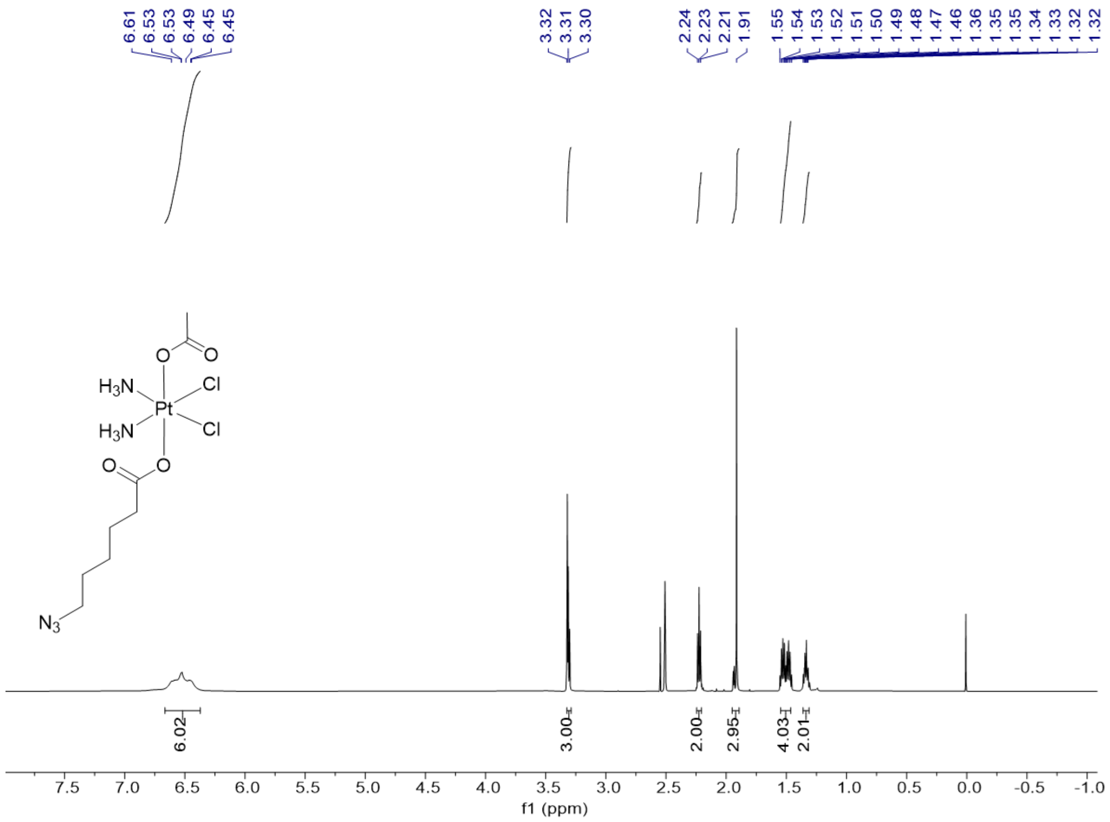

Figure S51. ^{1}H NMR spectrum of Cispt-AcOH-N_3 in DMSO-d_6.

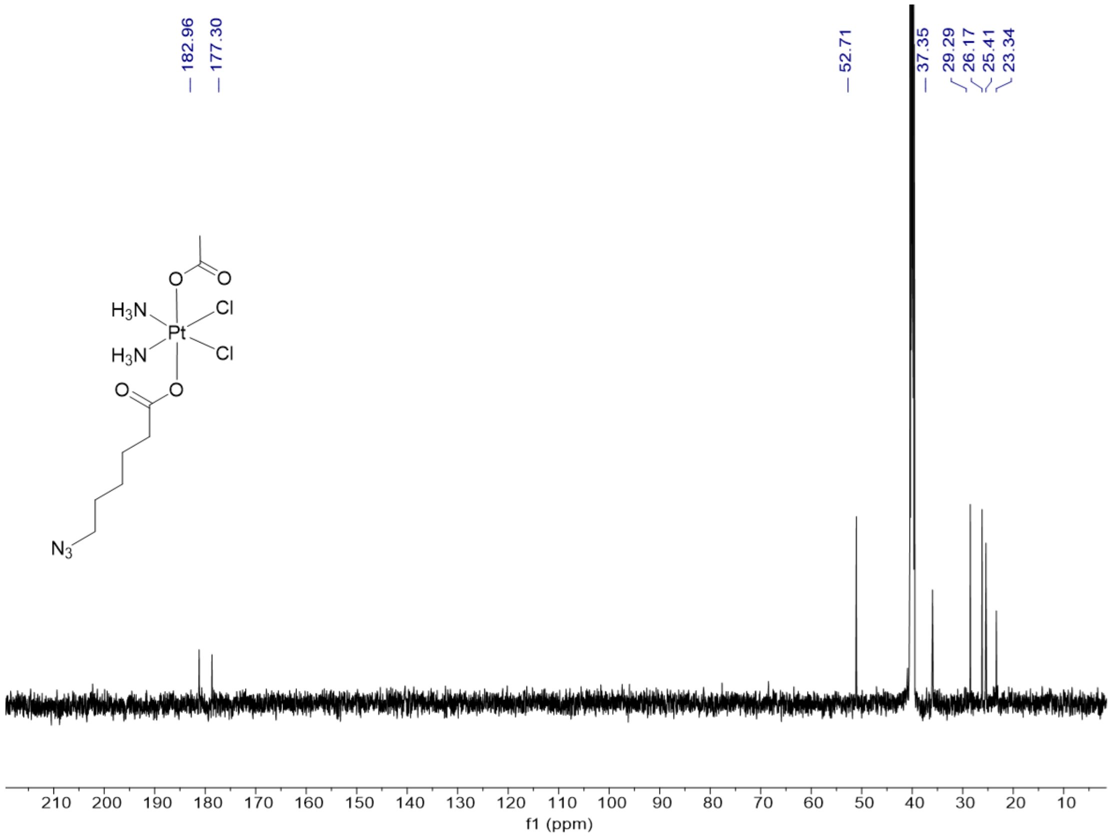

Figure S52. ^{13}C NMR spectrum of Cispt-AcOH-N_3 in DMSO-d_6.

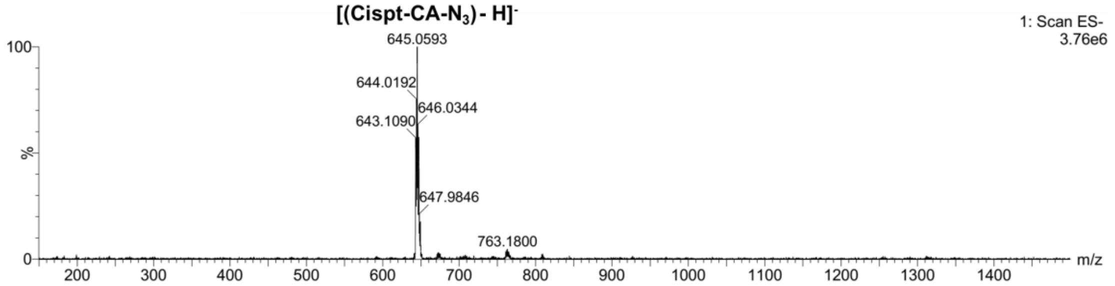

Figure S53. Mass spectrum of Cispt-CA-N_3.

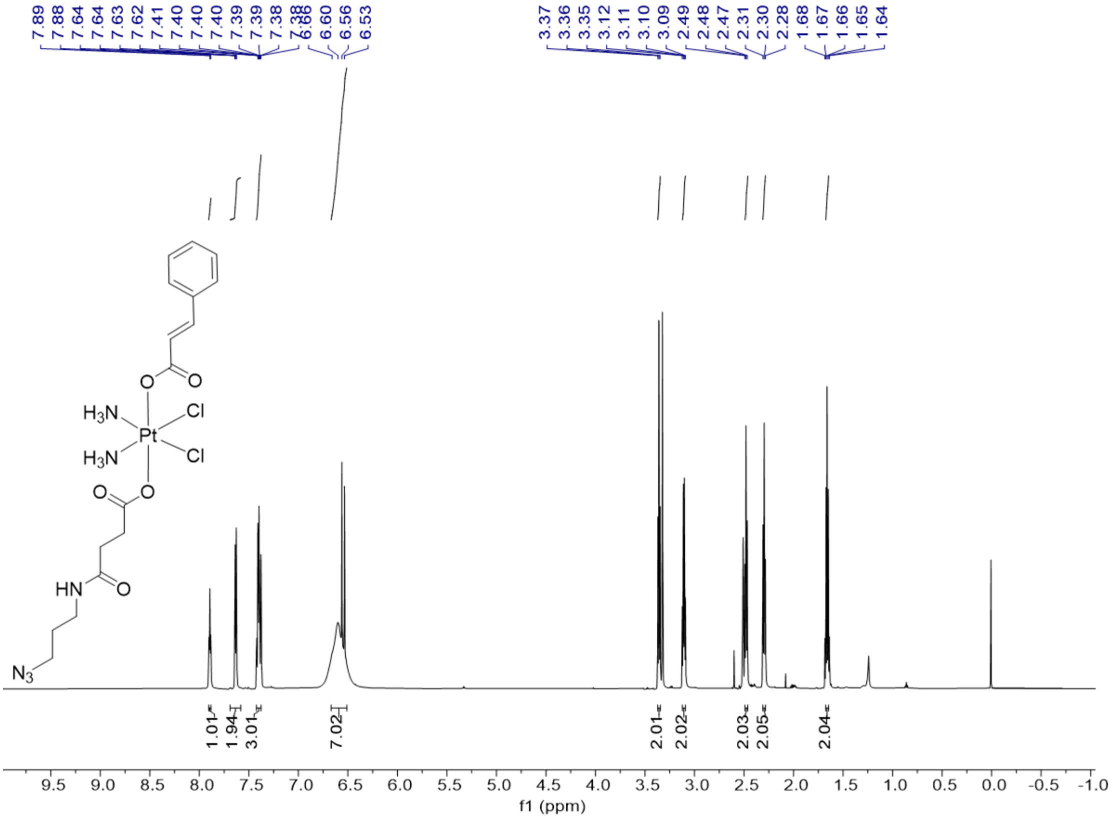

Figure S54. ^{1}H NMR spectrum of Cispt-CA-N_3 in DMSO-d_6.

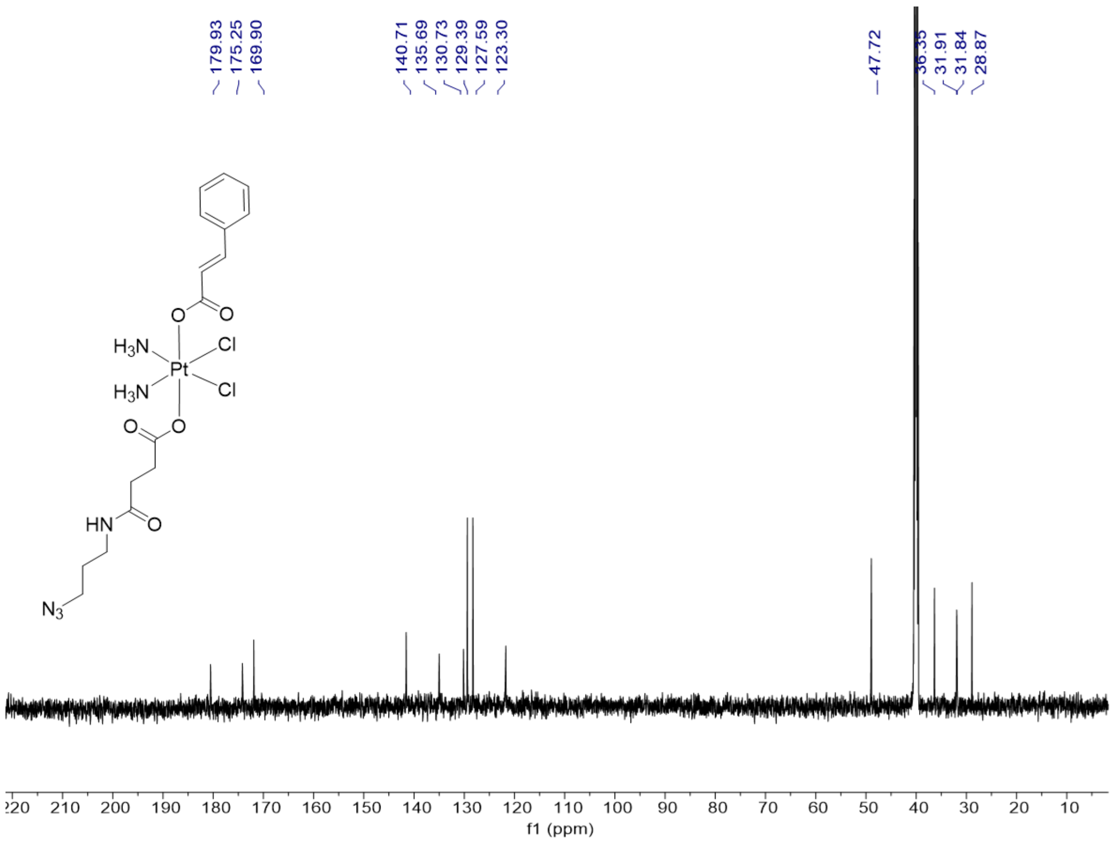

Figure S55. ^{13}C NMR spectrum of Cispt-CA-N_3 in DMSO-d_6.

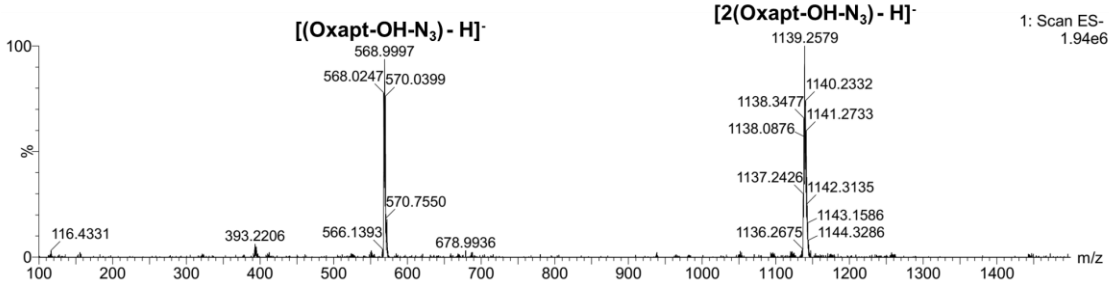

Figure S56. Mass spectrum of Oxapt-OH-N_3.

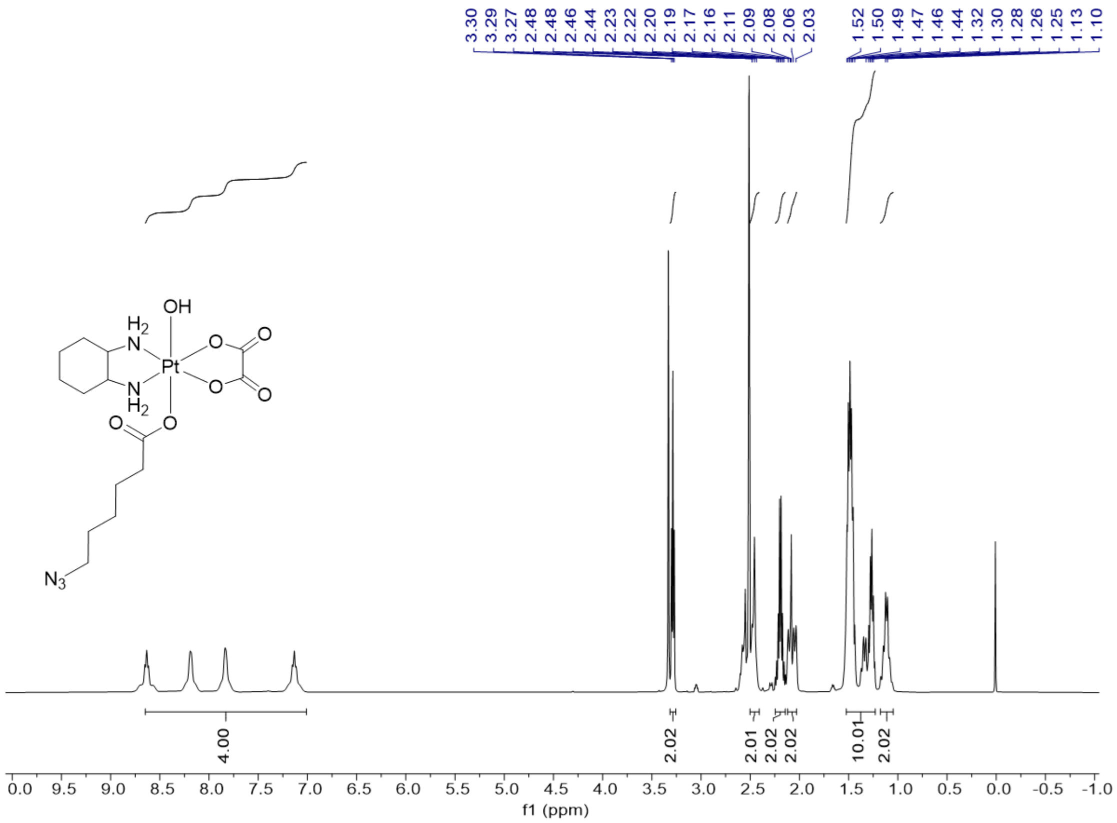

Figure S57. ^{1}H NMR spectrum of Oxapt-OH-N_3 in DMSO-d_6.

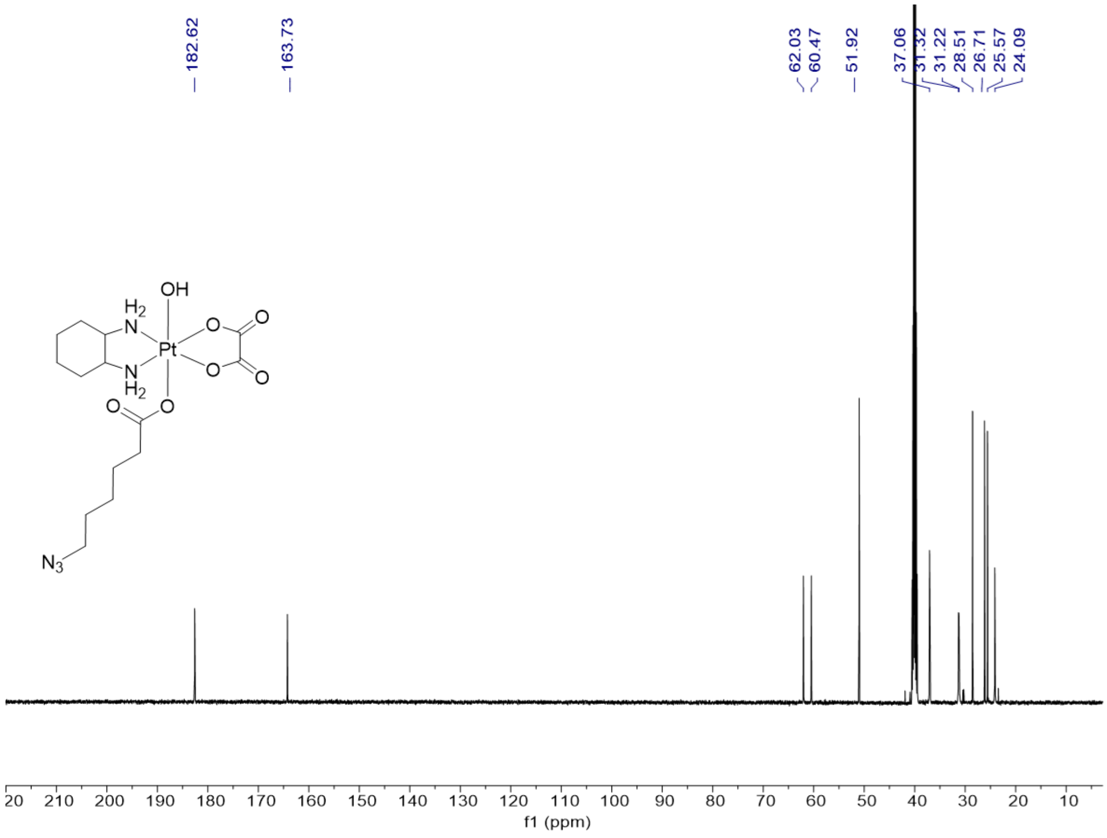

Figure S58. ^{13}C NMR spectrum of Oxapt-OH-N_3 in DMSO-d_6.

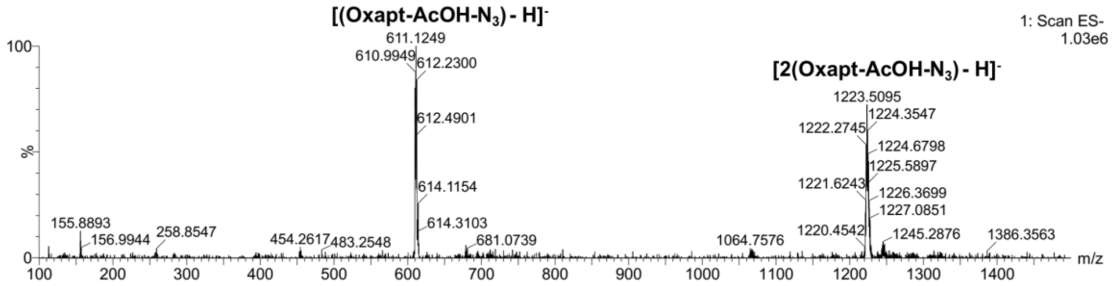

Figure S59. Mass spectrum of Oxapt-AcOH-N3.

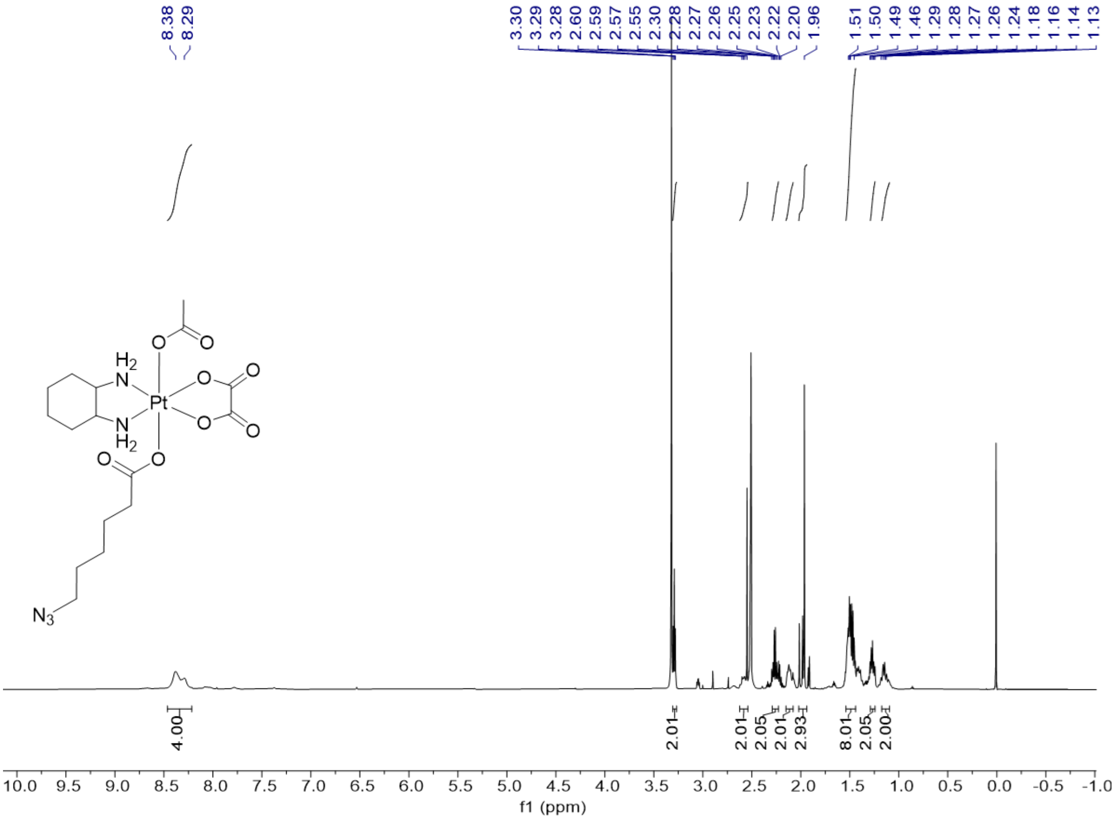

Figure S60. ^{1}H NMR spectrum of Oxapt-AcOH-N3 in DMSO-d_6.

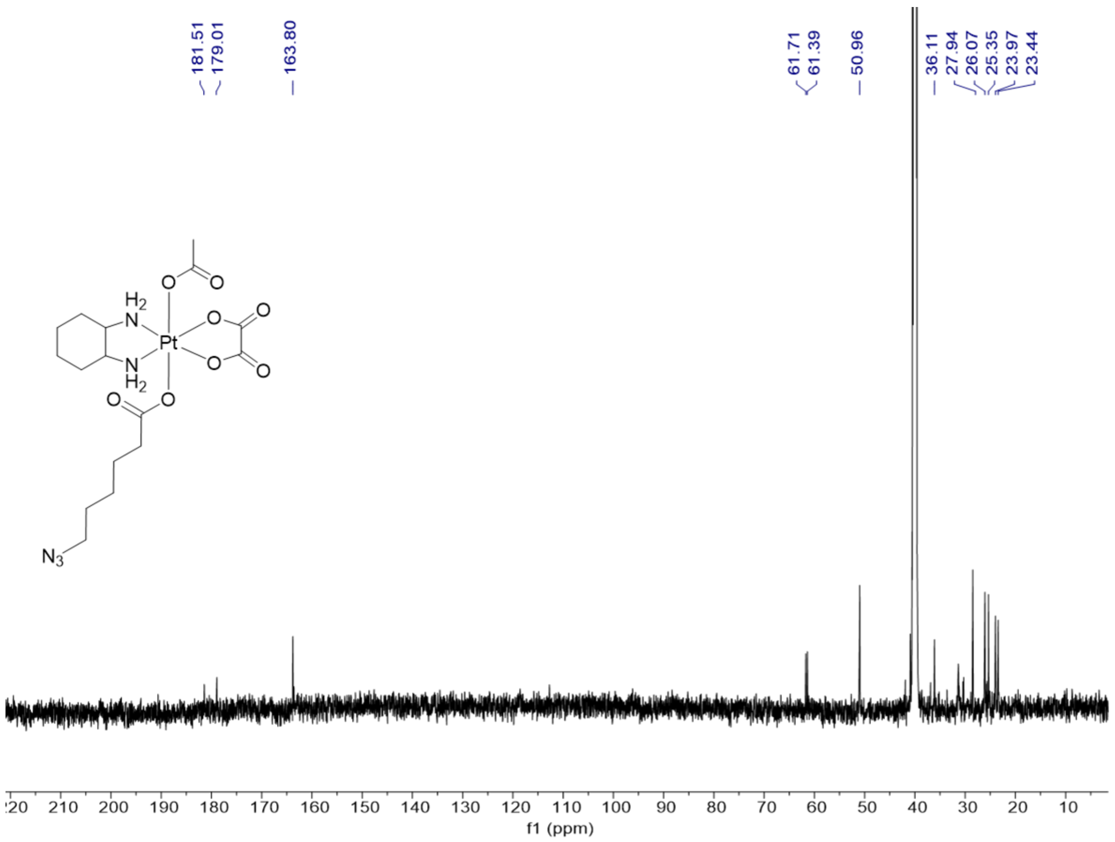

Figure S61. ^{13}C NMR spectrum of Oxapt-AcOH-N_3 in DMSO-d_6.

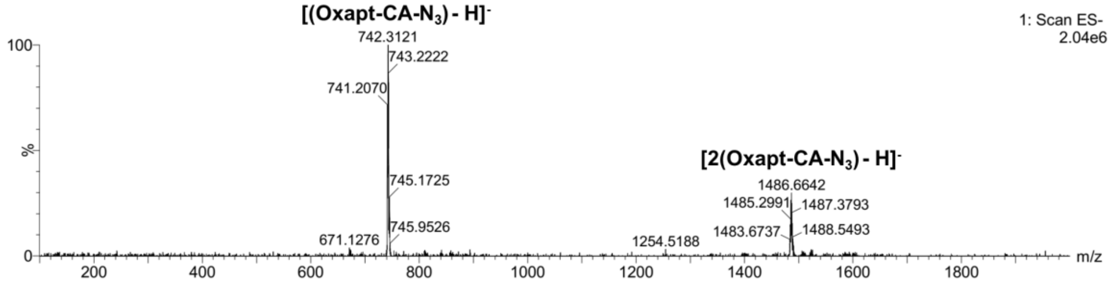

Figure S62. Mass spectrum of Oxapt-CA-N_3.

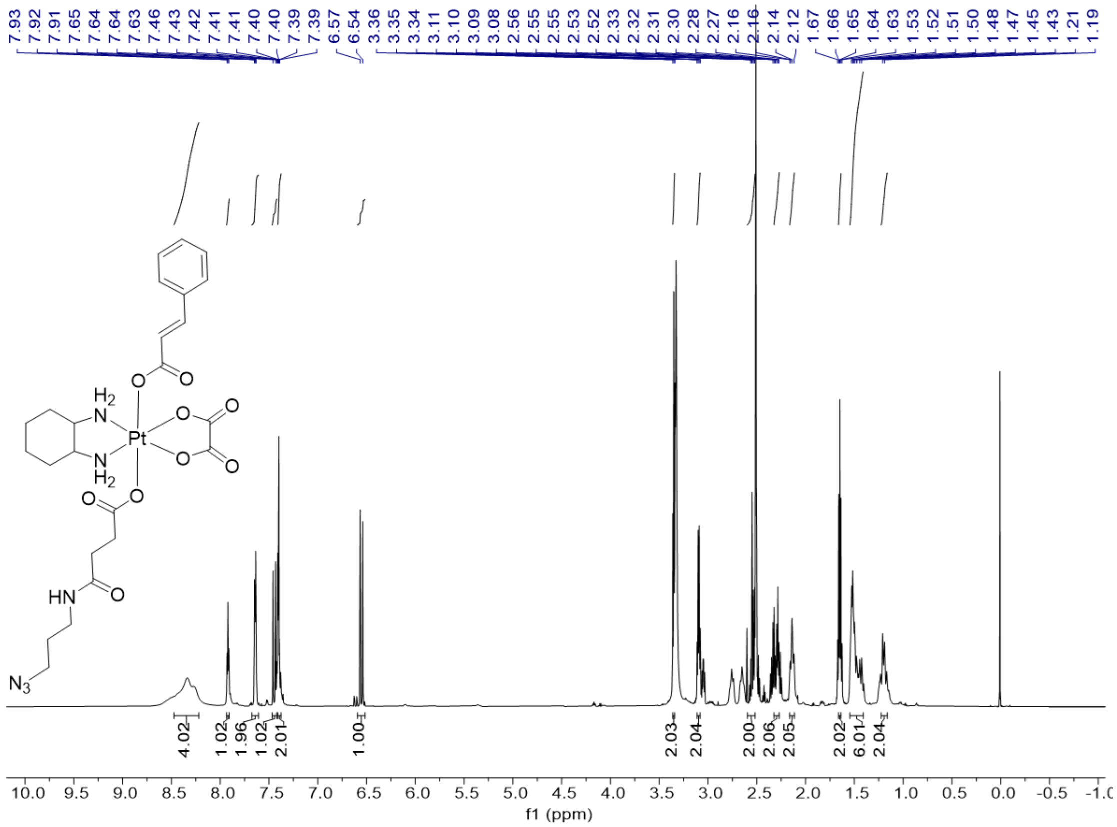

Figure S63. ^{1}H NMR spectrum of Oxapt-CA-N_3 in DMSO-d_6.

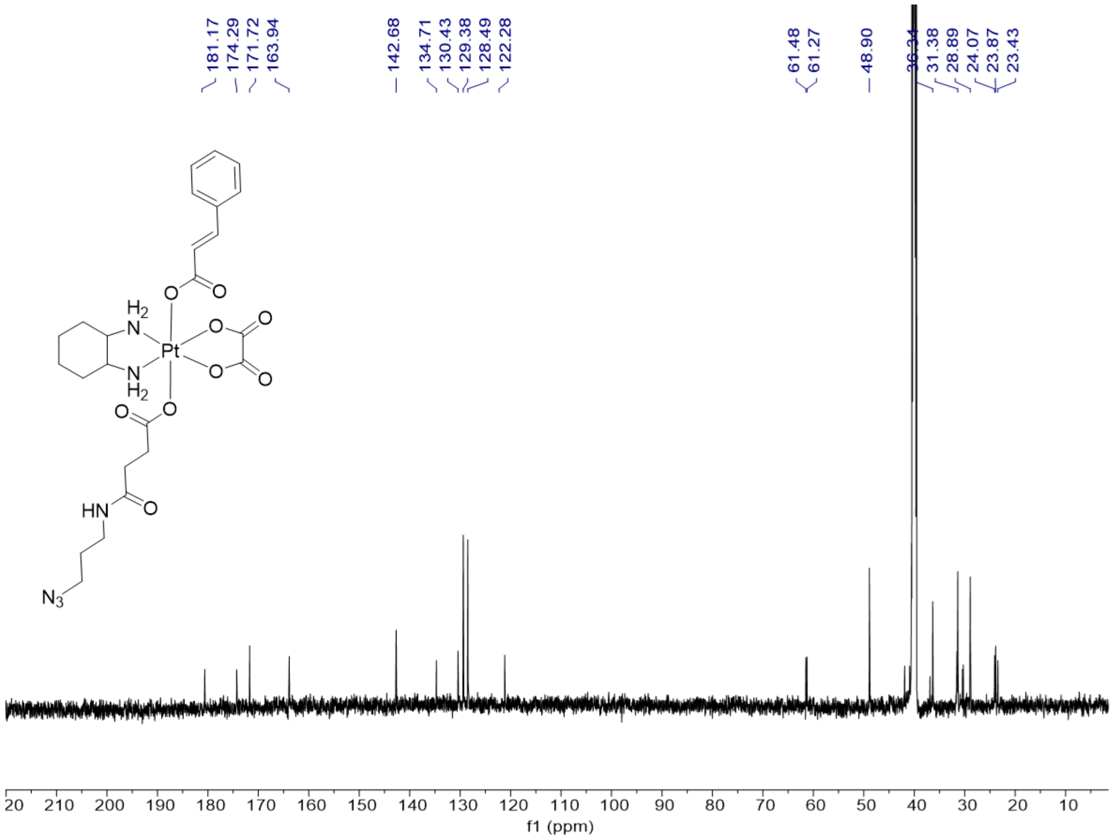

Figure S64. ^{13}C NMR spectrum of Oxapt-CA-N_3 in DMSO-d_6.

Supplementary References

1. Yin S, Xi R, Wu A *et al*. Patient-derived tumor-like cell clusters for drug testing in cancer therapy. *Sci Transl Med* 2020; **12:** eaaz1723.
2. Yin S, Yu Y, Wu N *et al*. Patient-derived tumor-like cell clusters for personalized chemo- and immunotherapies in non-small cell lung cancer. *Cell Stem Cell* 2024; **31:** 717-33.
3. Ng MSF, Kwok I, Tan L *et al*. Deterministic reprogramming of neutrophils within tumors. *Science* 2024; **383:** eadf6493.
4. Zhao Z, Zhang S, Jiang N *et al*. Patient-derived immunocompetent tumor organoids: A platform for chemotherapy evaluation in the context of T-cell recognition. *Angew Chem Int Ed* 2024; **63:** e202317613.
5. Zajac J, Novohradsky V, Markova L *et al*. Platinum (IV) derivatives with cinnamate axial ligands as potent agents against both differentiated and tumorigenic cancer stem rhabdomyosarcoma cells. *Angew Chem Int Ed* 2020; **59:** 3329-35.
6. Yang Y, Song Z, Tian T *et al*. Trimming crystallizable fragment (Fc) glycans enables the direct enzymatic transfer of biomacromolecules to antibodies as therapeutics. *Angew Chem Int Ed* 2023; **62:** e202308174.
